# Structural brain abnormalities in the common epilepsies assessed in a worldwide ENIGMA study

**DOI:** 10.1093/brain/awx341

**Published:** 2018-01-30

**Authors:** Christopher D Whelan, Andre Altmann, Juan A Botía, Neda Jahanshad, Derrek P Hibar, Julie Absil, Saud Alhusaini, Marina K M Alvim, Pia Auvinen, Emanuele Bartolini, Felipe P G Bergo, Tauana Bernardes, Karen Blackmon, Barbara Braga, Maria Eugenia Caligiuri, Anna Calvo, Sarah J Carr, Jian Chen, Shuai Chen, Andrea Cherubini, Philippe David, Martin Domin, Sonya Foley, Wendy França, Gerrit Haaker, Dmitry Isaev, Simon S Keller, Raviteja Kotikalapudi, Magdalena A Kowalczyk, Ruben Kuzniecky, Soenke Langner, Matteo Lenge, Kelly M Leyden, Min Liu, Richard Q Loi, Pascal Martin, Mario Mascalchi, Marcia E Morita, Jose C Pariente, Raul Rodríguez-Cruces, Christian Rummel, Taavi Saavalainen, Mira K Semmelroch, Mariasavina Severino, Rhys H Thomas, Manuela Tondelli, Domenico Tortora, Anna Elisabetta Vaudano, Lucy Vivash, Felix von Podewils, Jan Wagner, Bernd Weber, Yi Yao, Clarissa L Yasuda, Guohao Zhang, Nuria Bargalló, Benjamin Bender, Neda Bernasconi, Andrea Bernasconi, Boris C Bernhardt, Ingmar Blümcke, Chad Carlson, Gianpiero L Cavalleri, Fernando Cendes, Luis Concha, Norman Delanty, Chantal Depondt, Orrin Devinsky, Colin P Doherty, Niels K Focke, Antonio Gambardella, Renzo Guerrini, Khalid Hamandi, Graeme D Jackson, Reetta Kälviäinen, Peter Kochunov, Patrick Kwan, Angelo Labate, Carrie R McDonald, Stefano Meletti, Terence J O'Brien, Sebastien Ourselin, Mark P Richardson, Pasquale Striano, Thomas Thesen, Roland Wiest, Junsong Zhang, Annamaria Vezzani, Mina Ryten, Paul M Thompson, Sanjay M Sisodiya

**Affiliations:** 1 Imaging Genetics Center, Mark and Mary Stevens Neuroimaging and Informatics Institute, University of Southern California, Los Angeles, California, USA; 2 Department of Molecular and Cellular Therapeutics, Royal College of Surgeons in Ireland, Dublin, Ireland; 3 Translational Imaging Group, Centre for Medical Image Computing, University College London, London, UK; 4 Reta Lila Weston Institute and Department of Molecular Neuroscience, UCL Institute of Neurology, London WC1N 3BG, UK; 5 Department of Radiology, Hôpital Erasme, Universite Libre de Bruxelles, Brussels 1070, Belgium; 6 Department of Neurology and Neurosurgery, Montreal Neurological Institute, McGill University, Montreal, Quebec, Canada; 7 Department of Neurology, University of Campinas, Campinas, Brazil; 8 Epilepsy Center, Department of Neurology, Kuopio University, Kuopio, Finland; 9 Institute of Clinical Medicine, Neurology, University of Eastern Finland, Kuopio, Finland; 10 Pediatric Neurology Unit, Children’s Hospital A. Meyer-University of Florence, Italy; 11 IRCCS Stella Maris Foundation, Pisa, Italy; 12 Comprehensive Epilepsy Center, Department of Neurology, New York University School of Medicine, New York, USA; 13 Department of Physiology, Neuroscience and Behavioral Science, St. George’s University, Grenada, West Indies; 14 Institute of Molecular Bioimaging and Physiology of the National Research Council (IBFM-CNR), Catanzaro, Italy; 15 Magnetic Resonance Image Core Facility, IDIBAPS, Barcelona, Spain; 16 Department of Basic and Clinical Neuroscience, Institute of Psychiatry, Psychology and Neuroscience, King’s College London, UK; 17 Department of Computer Science and Engineering, The Ohio State University, USA; 18 Cognitive Science Department, Xiamen University, Xiamen, China; 19 Fujian Key Laboratory of the Brain-like Intelligent Systems, China; 20 Functional Imaging Unit, Institute of Diagnostic Radiology and Neuroradiology, University Medicine Greifswald, Greifswald, Germany; 21 Cardiff University Brain Research Imaging Centre, School of Psychology, Wales, UK; 22 Department of Neurosurgery, University Hospital, Freiburg, Germany; 23 Department of Neuropathology, University Hospital Erlangen, Germany; 24 Department of Molecular and Clinical Pharmacology, Institute of Translational Medicine, University of Liverpool, UK; 25 Department of Neurology and Epileptology, Hertie Institute for Clinical Brain Research, University of Tübingen, Tübingen, Germany; 26 Department of Diagnostic and Interventional Neuroradiology, University of Tübingen, Tübingen, Germany; 27 The Florey Institute of Neuroscience and Mental Health, Austin Campus, Melbourne, VIC, Australia; 28 Multimodal Imaging Laboratory, University of California San Diego, San Diego, California, USA; 29 Department of Psychiatry, University of California San Diego, San Diego, California, USA; 30 Neuroimaging of Epilepsy Laboratory, Montreal Neurological Institute and Hospital, Mcgill University, Montreal, Quebec, Canada; 31 Neuroradiology Unit, Children's Hospital A. Meyer, Florence, Italy; 32 “Mario Serio” Department of Experimental and Clinical Biomedical Sciences, University of Florence, Italy; 33 Instituto de Neurobiología, Universidad Nacional Autónoma de México. Querétaro, Querétaro, México; 34 Support Center for Advanced Neuroimaging (SCAN), University Institute for Diagnostic and Interventional Neuroradiology, Inselspital, University of Bern, Bern, Switzerland; 35 Central Finland Central Hospital, Medical Imaging Unit, Jyväskylä, Finland; 36 Neuroradiology Unit, Department of Head and Neck and Neurosciences, Istituto Giannina Gaslini, Genova, Italy; 37 Institute of Psychological Medicine and Clinical Neurosciences, Hadyn Ellis Building, Maindy Road, Cardiff, UK; 38 Department of Neurology, University Hospital of Wales, Cardiff, UK; 39 Department of Biomedical, Metabolic, and Neural Science, University of Modena and Reggio Emilia, NOCSE Hospital, Modena, Italy; 40 Melbourne Brain Centre, Department of Medicine, University of Melbourne, Parkville, VIC, 3052, Australia; 41 Department of Neurology, Royal Melbourne Hospital, Parkville, 3050, Australia; 42 Department of Neurology, University Medicine Greifswald, Greifswald, Germany; 43 Department of Epileptology, University Hospital Bonn, Bonn, Germany; 44 Department of Neurology, Philips University of Marburg, Marburg Germany; 45 Department of Neurocognition / Imaging, Life&Brain Research Centre, Bonn, Germany; 46 The Affiliated Chenggong Hospital of Xiamen University, Xiamen, China; 47 Department of Computer Science and Electrical Engineering, University of Maryland, Baltimore County, USA; 48 Centre de Diagnostic Per la Imatge (CDIC), Hospital Clinic, Barcelona, Spain; 49 Multimodal Imaging and Connectome Analysis Lab, Montreal Neurological Institute and Hospital, McGill University, Montreal, Quebec, Canada; 50 Medical College of Wisconsin, Department of Neurology, Milwaukee, WI, USA; 51 FutureNeuro Research Centre, RCSI, Dublin, Ireland; 52 Division of Neurology, Beaumont Hospital, Dublin 9, Ireland; 53 Department of Neurology, Hôpital Erasme, Universite Libre de Bruxelles, Brussels 1070, Belgium; 54 Neurology Department, St. James’s Hospital, Dublin 8, Ireland; 55 Department of Clinical Neurophysiology, University Medicine Göttingen, Göttingen, Germany; 56 Institute of Neurology, University “Magna Græcia”, Catanzaro, Italy; 57 Florey Department of Neuroscience and Mental Health, The University of Melbourne, Melbourne, VIC, Australia; 58 Maryland Psychiatric Research Center, Department of Psychiatry, University of Maryland School of Medicine, Maryland, USA; 59 Department of Medicine, University of Melbourne, Parkville, VIC, 3052, Australia; 60 Department of Neurology, King’s College Hospital, London, UK; 61 Pediatric Neurology and Muscular Diseases Unit, Department of Neurosciences, Rehabilitation, Ophthalmology, Genetics, Maternal and Child Health, University of Genoa, Genova, Italy; 62 Dept of Neuroscience, Mario Negri Institute for Pharmacological Research, Via G. La Masa 19, 20156 Milano, Italy; 63 Department of Medical and Molecular Genetics, King's College London, London SE1 9RT, UK; 64 Department of Clinical and Experimental Epilepsy, UCL Institute of Neurology, London, UK; 65 Chalfont Centre for Epilepsy, Bucks, UK

**Keywords:** epilepsy, MRI, thalamus, precentral gyrus

## Abstract

Progressive functional decline in the epilepsies is largely unexplained. We formed the ENIGMA-Epilepsy consortium to understand factors that influence brain measures in epilepsy, pooling data from 24 research centres in 14 countries across Europe, North and South America, Asia, and Australia. Structural brain measures were extracted from MRI brain scans across 2149 individuals with epilepsy, divided into four epilepsy subgroups including idiopathic generalized epilepsies (*n* =367), mesial temporal lobe epilepsies with hippocampal sclerosis (MTLE; left, *n* = 415; right, *n* = 339), and all other epilepsies in aggregate (*n* = 1026), and compared to 1727 matched healthy controls. We ranked brain structures in order of greatest differences between patients and controls, by meta-analysing effect sizes across 16 subcortical and 68 cortical brain regions. We also tested effects of duration of disease, age at onset, and age-by-diagnosis interactions on structural measures. We observed widespread patterns of altered subcortical volume and reduced cortical grey matter thickness. Compared to controls, all epilepsy groups showed lower volume in the right thalamus (Cohen’s *d* = −0.24 to −0.73; *P* < 1.49 × 10^−4^), and lower thickness in the precentral gyri bilaterally (*d* = −0.34 to −0.52; *P* < 4.31 × 10^−6^). Both MTLE subgroups showed profound volume reduction in the ipsilateral hippocampus (*d* = −1.73 to −1.91, *P* < 1.4 × 10^−19^), and lower thickness in extrahippocampal cortical regions, including the precentral and paracentral gyri, compared to controls (*d* = −0.36 to −0.52; *P* < 1.49 × 10^−4^). Thickness differences of the ipsilateral temporopolar, parahippocampal, entorhinal, and fusiform gyri, contralateral pars triangularis, and bilateral precuneus, superior frontal and caudal middle frontal gyri were observed in left, but not right, MTLE (*d* = −0.29 to −0.54; *P* < 1.49 × 10^−4^). Contrastingly, thickness differences of the ipsilateral pars opercularis, and contralateral transverse temporal gyrus, were observed in right, but not left, MTLE (*d* = −0.27 to −0.51; *P* < 1.49 × 10^−4^). Lower subcortical volume and cortical thickness associated with a longer duration of epilepsy in the all-epilepsies, all-other-epilepsies, and right MTLE groups (beta, *b* < −0.0018; *P* < 1.49 × 10^−4^). In the largest neuroimaging study of epilepsy to date, we provide information on the common epilepsies that could not be realistically acquired in any other way. Our study provides a robust ranking of brain measures that can be further targeted for study in genetic and neuropathological studies. This worldwide initiative identifies patterns of shared grey matter reduction across epilepsy syndromes, and distinctive abnormalities between epilepsy syndromes, which inform our understanding of epilepsy as a network disorder, and indicate that certain epilepsy syndromes involve more widespread structural compromise than previously assumed.

## Teaser

Structural MRI abnormalities are inconsistently reported in epilepsy. In the largest neuroimaging study to date, Whelan *et al.* report robust structural alterations across and within epilepsy syndromes, including shared volume loss in the thalamus, and widespread cortical thickness differences. The resulting neuroanatomical map will guide prospective studies of disease progression.

## Introduction

Epilepsy is a prevalent neurological disorder, comprising many different syndromes and conditions, affecting 0.6–1.5% of the population worldwide (Bell *et al.*, 2014). Approximately one-third of affected individuals do not respond to antiepileptic drug therapy (French, 2007). Alternative treatment options may not be appropriate (Englot *et al.*, 2011), and are not always effective (Téllez-Zenteno *et al.*, 2005; Englot *et al.*, 2011). The identification of shared biological disease pathways may help elucidate diagnostic and prognostic biomarkers and therapeutic targets, which, in turn, could help to optimize individual treatment (Pitkänen *et al.*, 2016). However, disease biology remains unexplained for most cases—especially in commonly occurring epilepsies.

Epilepsy is a network disorder typically involving widespread structural alterations beyond the putative epileptic focus (Bernhardt *et al.*, 2015; Vaughan *et al.*, 2016). Hippocampal sclerosis is a common pathological substrate of mesial temporal lobe epilepsy (MTLE), but extrahippocampal abnormalities are also frequently observed in MTLE, notably in the thalamus (Keller and Roberts, 2008; Coan *et al.*, 2014; Alvim *et al.*, 2016) and neocortex (Keller and Roberts, 2008; Bernhardt *et al.*, 2009*b*, 2010; Blanc *et al.*, 2011; Labate *et al.*, 2011; Vaughan *et al.*, 2016). Neocortical abnormalities are also reported in idiopathic generalized epilepsies (IGE) (Bernhardt *et al.*, 2009*a*), and many childhood syndromes (O’Muircheartaigh *et al.*, 2011; Vollmar *et al.*, 2011; Ronan *et al.*, 2012; Overvliet *et al.*, 2013). Thus, common epilepsies may be characterized by shared disturbances in distributed cortico-subcortical brain networks (Berg *et al.*, 2010), but the pattern, consistency and cause of these disturbances, and how they relate to functional decline (Vlooswijk *et al.*, 2010; Bernasconi, 2016; Nickels *et al.*, 2016), are largely unknown.

Currently, we lack reliable data from large cross-sectional neuroimaging, brain tissue, or biomarker studies in the common epilepsies. Brain tissue is not available from large cohorts of patients: common forms of epilepsy are often unsuitable for surgical treatment, so biopsied tissues are simply unavailable in sufficient numbers for research into disease biology. Brain-wide post-mortem studies also require extensive effort for comprehensive analysis. MRI offers detailed information on brain structure, but MRI measures from groups of individuals with and without epilepsy are not always consistent. For example, MTLE is associated with hippocampal sclerosis in up to 70% of brain MRI scans (Blümcke *et al.*, 2013). However, the effects of laterality, and the extent of extrahippocampal grey matter loss are inconsistently reported in studies of left versus right MTLE (Kemmotsu *et al.*, 2011; Liu *et al.*, 2016). Similarly, abnormalities of the basal ganglia, hippocampus, lateral ventricles, and neocortex have all been reported in IGE (Betting *et al.*, 2006), but most alterations are non-specific, and visual inspection of clinical MRI in IGE is typically normal (Woermann *et al.*, 1998). Genome-wide association studies (GWAS) have identified genetic variants associated with complex epilepsies by ‘lumping’ different epilepsy types together (International League Against Epilepsy Consortium on Complex Epilepsies, 2014), but MRI studies are typically of smaller scale, and have not widely explored whether distinct epilepsy syndromes share common structural abnormalities.

There are many sources of inconsistency in previously reported MRI findings. First, epileptic seizures and syndromes are diverse; classifications are often revised and contested (Berg *et al.*, 2010; Scheffer *et al.*, 2017). Second, most cross-sectional brain imaging studies are based on small samples (typically <50 cases), limiting the power to detect subtle group differences (Button *et al.*, 2013). Third, variability in scanning protocols, image processing, and statistical analysis may affect the sensitivity of brain measures across studies.

The Enhancing Neuro Imaging Genetics through Meta-Analysis (ENIGMA) Consortium was formed to address these issues (Bearden and Thompson, 2017). ENIGMA is a global initiative, combining large samples with coordinated image processing, and integrating genomic and MRI data across hundreds of research centres worldwide. Prior ENIGMA studies have identified genetic variants associated with variations in brain structure (Stein *et al.*, 2012; Hibar *et al.*, 2015, 2017*a*; Adams *et al.*, 2016), and have reliably characterized patterns of brain abnormalities in schizophrenia (van Erp *et al.*, 2016), major depression (Schmaal *et al.*, 2016), obsessive compulsive disorder (Boedhoe *et al.*, 2017), attention deficit hyperactivity disorder (Hoogman *et al.*, 2017), and many other brain illnesses (Thompson *et al.*, 2017). Large-scale, collaborative initiatives such as ENIGMA may improve our understanding of epilepsy, helping clinicians make more informed decisions and provide personalized treatment strategies (Ben-Menachem, 2016). Thus, we formed the Epilepsy Working Group of ENIGMA (‘ENIGMA-Epilepsy’) to apply coordinated, well-powered studies of imaging and genetic data in epilepsy.

Here, in the largest analysis of structural brain abnormalities in epilepsy to date, we ranked effect sizes for 16 subcortical and 68 cortical brain regions in 2149 individuals with epilepsy and 1727 healthy controls, using harmonized image processing, quality control, and meta-analysis. First, we grouped all epilepsies together, to determine whether biologically distinct syndromes show robust, common structural deficits. Second, we assessed a well-characterized form of epilepsy: MTLE with hippocampal sclerosis, analysing patients with left- and right-sided hippocampal sclerosis as independent groups. Third, we examined another major set of epilepsy syndromes: IGE. Finally, we studied all remaining epilepsies as a combined subgroup, to understand the relative contributions of IGE, MTLE-L, MTLE-R, and all other syndromes on shared patterns of structural compromise. We tested how age at scan, age of onset, and epilepsy duration affected brain structural measures. Based on existing neuroimaging (Gotman *et al.*, 2005; Bernhardt *et al.*, 2009*a*; Liu *et al.*, 2016), neurophysiological (Gotman *et al.*, 2005), neuropathological (Thom *et al.*, 2009), and genetic data (International League Against Epilepsy Consortium on Complex Epilepsies, 2014), we predicted that (i) biologically distinct epilepsy syndromes would exhibit shared patterns of structural abnormalities; (ii) MTLEs with left or right hippocampal sclerosis would show distinct patterns of hippocampal and extrahippocampal structural deficits; and (iii) IGEs would also display subcortical volume and cortical thickness differences, compared to healthy controls.

## Materials and methods

Each centre received approval from their local institutional review board or ethics committee. Written informed consent was provided according to local requirements (Supplementary Table 1).

### Experimental design

#### Participants

**Figure 1:**
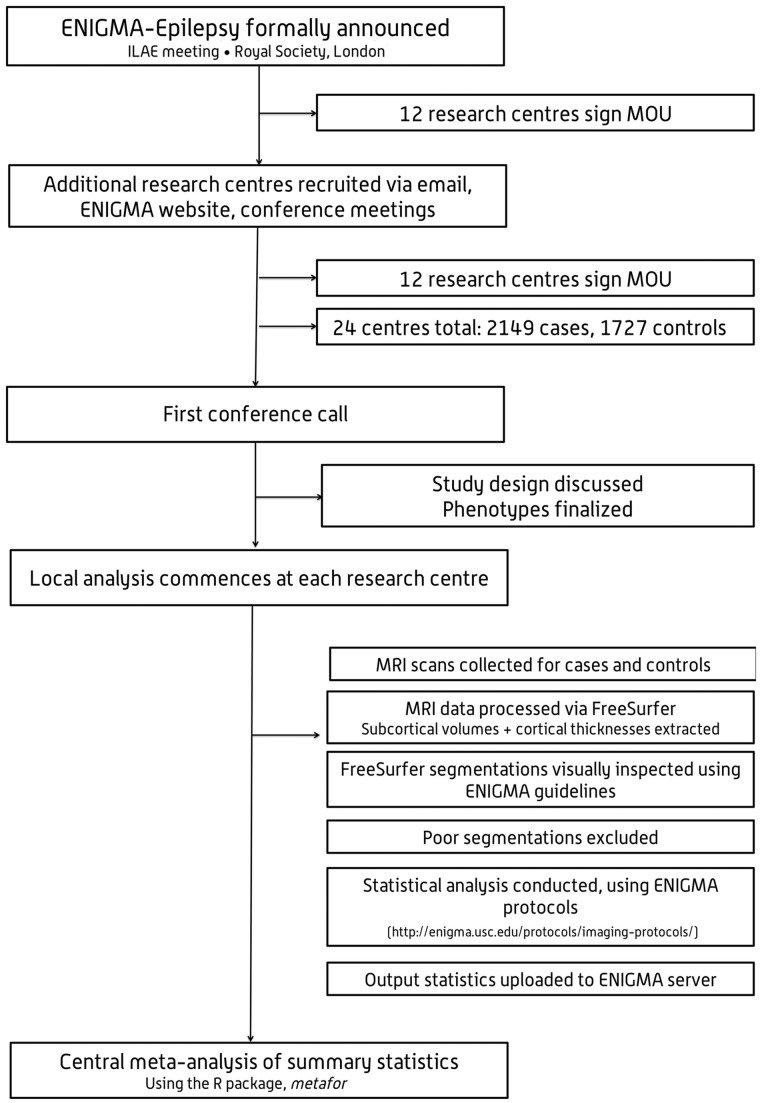
**Study flowchart.** ILAE = International League Against Epilepsy; MOU = memorandum of understanding.

Twenty-four cross-sectional samples from 14 countries were included in the study, totalling 2149 people with epilepsy and 1727 research centre-matched healthy control subjects (Fig. 1 and Table 1). The locations, dates, and periods of participant recruitment are provided in Supplementary Table 1. An epilepsy specialist assessed seizure and syndrome classifications at each centre, using International League Against Epilepsy terminology (Berg *et al.*, 2010). Participants were aged 18–55.

**Table 1:** ENIGMA - Epilepsy Working Group demographics, including age (in years), mean age at onset of epilepsy (in years), mean duration of illness (in years), sex, and case-control breakdown for participating sites

**Site name**	**Age controls (Mean ± SD)**	**Age cases (Mean ± SD)**	**Age of onset (Mean ± SD)**	**Duration of illness (Mean ± years)**	**Female controls**	**Female cases**	**Total controls**	**Total cases**	**MTLE-L cases**	**MTLE-R cases**	**IGE cases**	**‘Other’ cases**	**Total *n***
Bern	32.5 ± 9.39	30.48 ± 10.13	-	-	41	28	78	56	10	8	12	26	134
Bonn	40.11 ± 13.4	39.68 ± 13.4	16.86 ± 11.96	22.82 ± 14.18	40	60	77	108	71	37	0	0	185
BRI	34.73 ± 10.61	33.28 ± 10.59	17.9 ± 11.49	17.9 ± 12.93	49	46	112	79	10	13	18	38	191
Brussels	26.64 ± 4.34	33.79 ± 9.9	14.46 ± 10.13	19.02 ± 12.77	24	49	44	83	11	0 (4)	8	60	127
CUBRIC	28.04 ± 8.16	28.42 ± 8.06	13.56 ± 5.18	14.81 ± 9.91	34	34	48	48	0	0	44	0 (4)	96
EKUT_A	34.82 ± 11.38	33.58 ± 11.07	17.04 ± 11.09	16.84 ± 13.18	30	28	49	47	6	0	5	36	96
EKUT_B	35.33 ± 12.27	31.13 ± 10.74	17.32 ± 10.8	14.45 ± 11.14	9	18	18	24	0	0	16	8	42
EPICZ	30.48 ± 9.39	30.42 ± 10.13	-	-	59	71	116	113	19	27	0	67	229
EPIGEN_3.0	34.75 ± 9.36	36.2 ± 9.97	17.03 ± 13.7	18.93 ± 10.88	30	37	70	60	8	5	0	47	130
EPIGEN_1.5	31.7 ± 9.24	37.46 ± 10.69	14.51 ± 11.8	22.68 ± 14.28	24	35	47	52	27	25	0	0	99
Florence	35.29 ± 8.48	28 ± 7.77	12.69 ± 8.02	14.27 ± 8.06	8	12	14	31	0 (1)	0	5	25	45
Greifswald	42.26 ± 14.97	26.23 ± 7.49	28.12 ± 17.86	14.13 ± 12.81	60	21	99	39	0	0	39	0	138
IDIBAPS-HCP	33.13 ± 5.99	36.77 ± 9.52	18.07 ± 11.72	17.64 ± 10.51	29	67	52	115	17	36	0 (3)	59	167
KCL_CNS	31.68 ± 8.4	33.2 ± 8.9	13.22 ± 8.2	20.67 ± 11.23	54	50	101	96	5	0 (4)	32	55	197
KCL_CRF	28.73 ± 8.29	31.47 ± 11.33	23.13 ± 7.55	8.33 ± 9.99	16	7	26	15	0 (3)	0 (2)	0 (4)	6	41
Kuopio	25.16 ± 1.55	33.35 ± 11.21	24 ± 13.22	9.35 ± 11.23	33	135	67	240	0	9	36	195	307
MNI	30.74 ± 7.38	32.53 ± 9.92	16.48 ± 9.72	16.05 ± 11.32	21	71	46	128	45	38	0	45	174
NYU	30.1 ± 10.36	33.23 ± 9.66	16.96 ± 11.27	16.43 ± 12.7	62	93	118	159	8	11	36	104	277
RMH	39.35 ± 20.26	38.08 ± 15.91	28.23 ± 17.98	10.18 ± 12.65	12	70	28	146	22	13	25	86	174
UCSD	36.89 ± 15.1	37.67 ± 11.79	19.32 ± 14.77	18.8 ± 15.36	16	22	37	43	14	8	0	21	80
UNAM	33.2 ± 12.29	31.47 ± 11.81	16.26 ± 11.33	15.03 ± 12.53	25	24	35	36	10	10	0	16	71
UNICAMP	34.39 ± 10.45	39.98 ± 10.25	12.07 ± 9.52	27.96 ± 12.54	249	183	398	291	107	84	40	60	689
UNIMORE	28.47 ± 5.25	28.36 ± 10.26	12.58 ± 8.13	14.34 ± 10.94	20	47	34	82	0 (3)	0 (2)	40	37	116
XMU	31.54 ± 6.99	28.79 ± 9.06	17.04 ± 12.2	11.76 ± 8.78	4	20	13	58	25	15	11	7	71
**Combined**	**33.31 ± 9.91**	**34.36 ± 10.65**	**17.63 ± 11.47**	**17.42 ± 11.99**	**949**	**1228**	**1727**	**2149**	**415**	**339**	**367**	**1028**	**3876**

Also provided is the total number of MTLE cases with left hippocampal sclerosis, MTLE cases with right hippocampal sclerosis, IGE and all-other-epilepsies (‘other’) cases per site. Research centres with fewer than five participants for a given phenotype are marked as ‘0’ for that phenotype, with the original sample size noted in parentheses.

SD = standard deviation.

To test for shared and syndrome-specific structural alterations, analyses included one group combining all epilepsies (‘all-epilepsies’; *n* = 2149), and four stratified subgroups: (i) left MTLE with left hippocampal sclerosis (MTLE-L; *n* = 415); (ii) right MTLE with right hippocampal sclerosis (MTLE-R; *n* = 339); (iii) IGE (*n* = 367); and (iv) all other epilepsies (*n* = 1028). Supplementary Table 2 lists all syndromic diagnoses included in the aggregate ‘all-epilepsies’ group. For the MTLE subgroups, we included anyone with the typical electroclinical constellation (Berg *et al.*, 2010), and a neuroradiologically-confirmed diagnosis of unilateral hippocampal sclerosis on clinical MRI. Participants were included in the IGE subgroup if they presented with tonic-clonic, absence or myoclonic seizures with generalized spike-wave discharges on EEG. Participants were included in the ‘all-other-epilepsies’ subgroup if they were diagnosed with non-lesional MTLE (43.3%), occipital (1.67%), frontal (8.78%), or parietal lobe epilepsy (0.84%), focal epilepsies not otherwise specified (37.03%), or another unclassified syndrome (8.37%; Supplementary Table 2). We excluded participants with a progressive disease (e.g. Rasmussen’s encephalitis), malformations of cortical development, tumours or previous neurosurgery.

#### MRI data collection and processing

Structural T_1_-weighted MRI brain scans were collected at the 24 participating centres. Scanning details are provided in Supplementary Table 3. T_1_-weighted images from cases and controls were analysed at each site using FreeSurfer 5.3.0, for automated analysis of brain structure (Fischl, 2012). Volumetric measures were extracted for 12 subcortical grey matter regions (six left and six right, including the amygdala, caudate, nucleus accumbens, pallidum, putamen, and thalamus), the left and right hippocampi, and the left and right lateral ventricles. Cortical thickness measures were extracted for 34 left-hemispheric grey matter regions, and 34 right-hemispheric grey matter regions (68 total; Supplementary Table 4). Visual inspections of subcortical and cortical segmentations were conducted following standardized ENIGMA protocols (http://enigma.usc.edu), used in prior genetic studies of brain structure (Stein *et al.*, 2012; Hibar *et al.*, 2015, 2017*a*; Adams *et al.*, 2016), and large-scale case-control studies of neuropsychiatric illnesses (Schmaal *et al.*, 2015, 2016; Hibar *et al.*, 2016; van Erp *et al.*, 2016; Boedhoe *et al.*, 2017). Analysts were blind to participants’ diagnoses. Each analyst was instructed to execute a series of standardized bash scripts, identifying participants with volumetric or thickness measures greater or less than 1.5 times the interquartile range as outliers. Outlier data were then visually inspected, by overlaying the participant’s cortical segmentations on their whole-brain anatomical images. If the blinded local analyst judged any structure as inaccurately segmented, that structure was omitted from the analysis. The Supplementary material provides further information.

### Statistical analysis

#### Participant demographics

All research centres tested for differences in age between individuals with epilepsy and controls using an unpaired, two-tailed *t*-test in the *R* statistics package (https://www.r-project.org). Each centre also tested for sex differences between individuals with epilepsy and controls using a chi-squared test in SPSS Statistics package (IBM Corp., Version 21.0).

#### Meta-analytical group comparisons

Each research centre tested for case-versus-control differences using multiple linear regressions (via the *lm* function implemented in *R*), where a binary indicator of diagnosis (0 = healthy control, 1 = person with epilepsy) was the predictor of interest, and the volume or thickness of a specified brain region was the outcome measure. We calculated effect size estimates across all brain regions using Cohen’s *d*, adjusting for age, sex and intracranial volume (ICV). ICV is a reliable, indirect measure of head size (Hansen *et al.*, 2015), used as a covariate in other large-scale ENIGMA collaborations (Schmaal *et al.*, 2015, 2016; Hibar *et al.*, 2016; van Erp *et al.*, 2016; Boedhoe *et al.*, 2017). Cohen’s *d* effect sizes and regression beta coefficients were pooled across centres using a random-effects, restricted maximum likelihood method of meta-analysis via the *R* package, *metafor* (Viechtbauer, 2010). The Supplementary material provides additional details.

#### Meta-analytical regression with clinical variables

Each centre conducted a series of linear regressions, testing the association between subcortical volume or cortical thickness, and: (i) age at onset of epilepsy; and (ii) duration of epilepsy. All centres tested for interactions between diagnosis of epilepsy (including syndrome groups) and age at time of scan. Beta values representing the unstandardized slopes of each regression were extracted for each analysis. Sex and ICV were included as covariates in all secondary analyses.

#### Correction for multiple comparisons

We conducted four independent regressions (one case versus control regression, and three regressions with clinical variables) across 84 regions of interest, adjusting the statistical significance threshold to *P*_thresh_ < 1.49 × 10^−4^ to correct for 336 comparisons. To account for correlations between tests, we also applied a less conservative adjustment for false discovery rate (FDR), using the Benjamini and Hochberg method (Benjamini and Hochberg, 1995). For clarity, we report only *P*-values significant after stringent Bonferroni correction; FDR-adjusted *P*-values are summarized in the Supplementary material.

#### Power analyses

Across all regions of interest, we calculated the sample sizes necessary to achieve 80% power to detect case-control differences, given the observed effect sizes at each region of interest, based on two-tailed *t*-tests, using G*Power Version 3.1. For each region of interest, we also estimated *N_80_*: the total number of samples required, per group, to achieve 80% power to detect group differences using a *t*-test at the threshold of *P* < 0.05 (two-tailed).

## Results

### Participant demographics

The sample size-weighted mean age across all epilepsy samples was 34.4 (range: 26.2–40) years, and the weighted mean age of healthy controls was 33.3 (range: 25.2–42.3) years. The weighted mean age at onset of epilepsy and duration of epilepsy were 17.6 (range: 12.1–28.2) years and 17.4 (range: 8.3–28) years, respectively. Females comprised 57% of the total epilepsy sample (range: 34–75% by individual sample), and 53% of the controls (range: 31–71% by individual sample). Case-control differences in age were observed at 8 of 24 research centres, and case-control differences in sex were observed at 2 of 24 research centres (Supplementary Table 5); hence, age and sex were included as covariates in all group comparisons.

### Volumetric findings

**Figure 2:**
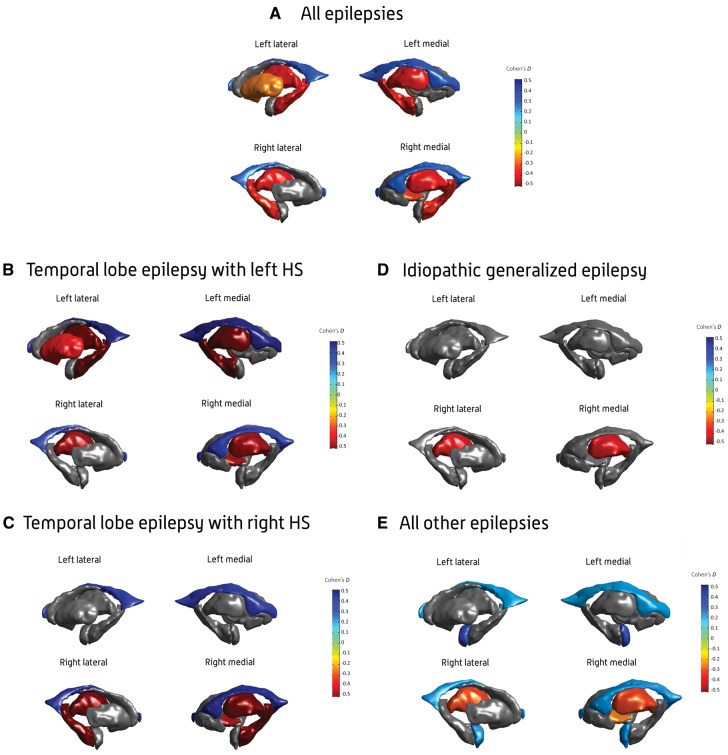
**Subcortical volume findings.** Cohen’s *d* effect size estimates for case-control differences in subcortical volume, across the (**A**) all-epilepsies, (**B**) mesial temporal lobe epilepsies with left hippocampal sclerosis (HS; MTLE-L), (**C**) mesial temporal lobe epilepsies with right hippocampal sclerosis (MTLE-R), (**D**) idiopathic generalized epilepsies (IGE), and (**E**) all-other-epilepsies groups. Cohen’s *d* effect sizes were extracted using multiple linear regressions, and pooled across research centres using random-effects meta-analysis. Subcortical structures with *P*-values < 1.49 × 10^−4^ are shown in heatmap colours; strength of heat map is determined by the size of the Cohen’s *d* (*d* < 0 = blue, *d* > 0 = yellow/red). Image generated using MATLAB, with annotations added using Adobe Photoshop. An interactive version of this figure is available online, via ‘ENIGMA-Viewer’: http://enigma-viewer.org/ENIGMA_epilepsy_subcortical.html. See Supplementary material for guidelines on how to use the interactive visualization.

Compared to controls, the aggregate all-epilepsies group exhibited lower volumes in the left (*d* = −0.36; *P = *1.31 × 10^−6^) and right thalamus (*d* = −0.37; *P = *7.67 × 10^−14^), left (*d* = −0.35; *P = *3.04 × 10^−7^) and right hippocampus (*d* = −0.34; *P = *6.63 × 10^−10^), and the right pallidum (*d* = −0.32; *P = *8.32 × 10^−9^). Conversely, the left (*d* = 0.29; *P = *2.14 × 10^−12^) and right (*d* = 0.27; *P = *3.73 × 10^−15^) lateral ventricles were enlarged across all epilepsies when compared to controls (Table 2 and Fig. 2A). A supplementary analysis of all-epilepsies, excluding individuals with hippocampal sclerosis or other lesions, revealed similar patterns of volume loss in the right thalamus and pallidum, and bilaterally enlarged ventricles; however, volume differences were not observed in the hippocampus (Supplementary Table 6).

**Table 2:** Effect size differences between epilepsy cases and healthy controls (Cohen’s *d*) for the mean volume of subcortical structures, controlling for age, sex and intracranial volume

Structure	Phenotype	Cohen’s *d*	SE	Z score	95% CI	*P-*value	*I*^2^	*N*_80_	Number of controls	Number of cases
Amygdala (LH)	All-other-epilepsies	0.327	0.065	5.024	0.199–0.455	5.05 x 10^−7^	45.470	148	1448	998
Amygdala (RH)	All-other-epilepsies	0.218	0.057	3.799	0.106–0.33	1.46 x 10^−4^	31.256	335	1422	989
Hippocampus (LH)	MTLE-L	−1.728	0.191	−9.056	−2.102 to −1.354	1.35 x 10^−19^	85.532	7	1412	410
	All epilepsies	−0.353	0.069	−5.121	−0.488 to −0.217	3.04 x 10^−7^	71.845	127	1707	2125
Hippocampus (RH)	MTLE-R	−1.906	0.15	−12.694	−2.2 to −1.611	6.36 x 10^−37^	72.476	6	1286	336
	All epilepsies	−0.336	0.054	−6.175	−0.443 to −0.229	6.63 x 10^−10^	54.801	141	1719	2129
Lateral ventricle (LH)	MTLE-L	0.465	0.089	5.203	0.289–0.640	1.96 x 10^−7^	43.124	74	1417	414
	MTLE-R	0.39	0.081	4.808	0.231–0.549	1.52 x 10^−6^	26.750	105	1291	338
	All epilepsies	0.288	0.041	7.025	0.207–0.368	2.14 x 10^−12^	23.338	191	1722	2135
	All-other-epilepsies	0.198	0.045	4.373	0.109–0.287	1.23 x 10^−5^	0.218	402	1452	996
Lateral ventricle (RH)	MTLE-R	0.444	0.065	6.867	0.317−0.57	6.57 x 10^−12^	0.003	81	1292	338
	MTLE-L	0.363	0.093	3.917	0.1814−0.544	8.95 x 10^−5^	47.227	121	1418	414
	All epilepsies	0.268	0.034	7.864	0.2−0.334	3.73 x 10^−15^	0	220	1722	2137
	All-other-epilepsies	0.212	0.046	4.581	0.122−0.303	4.62 x 10^−6^	3.528	350	1453	996
Pallidum (RH)	MTLE-L	−0.452	0.09	−5.009	−0.628 to −0.275	5.48 x 10^−7^	43.985	78	1406	414
	MTLE-R	−0.451	0.089	−5.071	−0.624 to −0.276	3.96 x 10^−7^	36.432	79	1278	332
	All epilepsies	−0.316	0.055	−5.762	−0.424 to −0.208	8.32 x 10^−9^	55.575	159	1710	2112
	All-other-epilepsies	−0.235	0.060	−3.942	−0.352 to −0.118	8.07 x 10^−5^	36.141	286	1440	976
Putamen (LH)	MTLE-L	−0.385	0.079	−4.878	−0.539 to −0.23	1.07 x 10^−6^	28.474	107	1352	410
Thalamus (LH)	MTLE-L	−0.843	0.126	−6.693	−1.089 to −0.595	2.19 x 10^−11^	70.462	24	1384	408
	All epilepsies	−0.358	0.074	−4.839	−0.503 to −0.213	1.31 x 10^−6^	75.649	124	1687	2104
Thalamus (RH)	MTLE-R	−0.727	0.103	−7.066	−0.928 to −0.525	1.60 x 10^−12^	51.499	31	1285	335
	MTLE-L	−0.462	0.117	−3.941	−0.691 to −0.232	8.12 x 10^−5^	67.376	75	1412	414
	IGE	−0.403	0.087	−4.633	−0.574 to −0.233	3.60 x 10^−6^	39.715	98	1210	363
	All epilepsies	−0.368	0.049	−7.476	−0.464 to −0.271	7.67 x 10^−14^	44.822	117	1716	2137
	All-other-epilepsies	−0.305	0.047	−6.502	−0.397 to −0.213	7.92 x 10^−11^	4.985	170	1446	998

CI = confidence interval; LH = left hemisphere; RH = right hemisphere; SE = standard error; *I*^2 ^= heterogeneity index; *N*_80_ = number of subjects required in each group to yield 80% power to detect significant group differences (*P* < 0.05, two-tailed). Uncorrected *P*-values are reported. Subcortical structures that failed to survive Bonferroni correction (*P* < 1.49 x 10^−4^) are not reported (see ‘Materials and methods’ section for statistical threshold determination). See Supplementary material for a full list of volume differences with adjustment for false discovery rate (FDR).

The MTLE-L subgroup showed lower volumes in the left hippocampus (*d* = −1.73; *P = *1.35 × 10^−19^), left (*d* = *P = *2.19 × 10^−11^) and right thalamus (*d* = −0.46; *P* = 8.12 × 10^−5^), left putamen (*d* = −0.39; *P = *1.07 × 10^−6^), and right pallidum (*d* = −0.45; *P = *5.48 × 10^−7^). As in the overall group comparison, we observed larger left (*d* = 0.47; *P = *1.96 × 10^−7^) and right lateral ventricles (*d* = 0.36; *P = *8.95 × 10^−5^) in MTLE-L patients relative to controls (Table 2 and Fig. 2B).

The MTLE-R subgroup showed lower volumes across a number of regions in the right hemisphere only, including the hippocampus (*d* = −1.91; *P = *6.36 × 10^−37^), thalamus (*d* = −0.73; *P = *1.6 × 10^−12^), and pallidum (*d* = −0.45; *P = *3.96 × 10^−7^), together with increased volumes of the left (*d* = 0.39; *P = *1.52 × 10^−6^) and right lateral ventricles (*d* = 0.44; *P = *6.57 × 10^−12^) compared to controls (Table 2 and Fig. 2C).

The IGE subgroup showed lower volumes in the right thalamus (*d* = −0.4; *P = *3.6 × 10^−6^) compared to controls (Table 2 and Fig. 2D).

The all-other-epilepsies subgroup showed lower volumes in the right thalamus (*d* = −0.31; *P = *7.9 × 10^−11^) and the right pallidum (*d* = −0.24; *P = *8.1 × 10^−5^) compared to controls. The all-other-epilepsies subgroup also showed significant enlargements of the left (*d* = 0.33; *P = *5.1 × 10^−7^) and right amygdala (*d* = 0.22; *P = *1.46 × 10^−4^), and the left (*d* = 0.2; *P = *1.2 × 10^−5^) and right lateral ventricles (*d* = 0.21; *P = *4.62 × 10^−6^) compared to controls (Table 2 and Fig. 2E).

All volume differences can be visualized using the interactive ENIGMA-Viewer tool (Zhang *et al.*, 2017), at http://enigma-viewer.org/ENIGMA_epilepsy_subcortical.html (Supplementary material). Volume differences significant after FDR adjustment can also be visualized at http://enigma-viewer.org/ENIGMA_epilepsy_subcortical_fdr.html (Supplementary Tables 26–30).

### Cortical thickness findings

**Figure 3:**
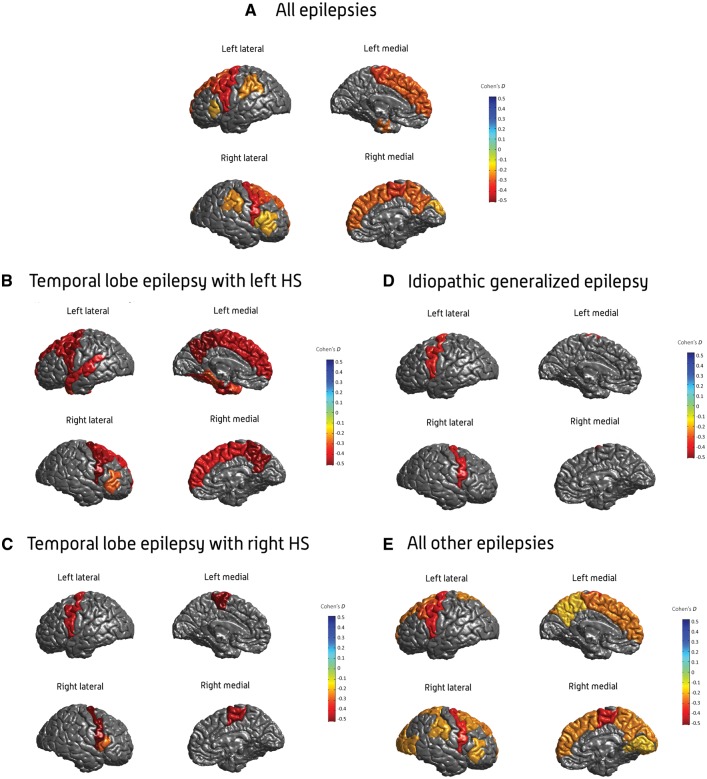
**Cortical thickness findings.** Cohen’s *d* effect size estimates for case-control differences in cortical thickness, across the (**A**) all-epilepsies, (**B**) mesial temporal lobe epilepsies with left hippocampal sclerosis (MTLE-L), (**C**) mesial temporal lobe epilepsies with right hippocampal sclerosis (MTLE-R), (**D**) idiopathic generalized epilepsies (IGE), and (**E**) all-other-epilepsies groups. Cohen’s *d* effect sizes were extracted using multiple linear regressions, and pooled across research centres using random-effects meta-analysis. Cortical structures with *P*-values < 1.49 × 10^−4^ are shown in heatmap colours; strength of heat map is determined by the size of the Cohen’s *d* (*d* < 0 = blue, *d* > 0 = yellow/red). Image generated using MATLAB with annotations added using Adobe Photoshop. An interactive version of this figure is available online, via ‘ENIGMA-Viewer’: http://enigma-viewer.org/ENIGMA_epilepsy_cortical.html. See Supplementary material for guidelines on how to use the interactive visualization. HS = hippocampal sclerosis.

The all-epilepsies group showed reduced thickness of cortical grey matter across seven regions bilaterally, including the left (*d* = −0.38; *P = *1.82 × 10^−18^) and right precentral gyri (*d* = −0.4; *P = *8.85 × 10^−20^), left (*d* = −0.32; *P = *2.11 × 10^−15^) and right caudal middle frontal gyri (*d* = −0.31; *P = *2.09 × 10^−9^), left (*d* = −0.31; *P = *2.05 × 10^−6^) and right paracentral gyri (*d* = −0.32; *P = *2.19 × 10^−9^), left (*d* = −0.19; *P = *1.29 × 10^−4^) and right pars triangularis (*d* = −0.2; *P = *4.25 × 10^−8^), left (*d* = −0.28; *P = *1.51 × 10^−7^) and right superior frontal gyri (*d* = −0.27; *P* = 4.49 × 10^−6^), left (*d* = −0.19; *P = *1.05 × 10^−5^) and right transverse temporal gyri (*d* = −0.18; *P* = 2.81 × 10^−5^), and left (*d* = −0.23; *P = *9.87 × 10^−5^) and right supramarginal gyri (*d* = −0.22; *P = *5.24 × 10^−5^). The all-epilepsies group also showed unilaterally thinner right cuneus (*d* = −0.2; *P = *9.68 × 10^−8^), right pars opercularis (*d* = −0.18; *P = *6.48 × 10^−7^), right precuneus (*d* = −0.28; *P = *2.7 × 10^−5^), and left entorhinal gyrus (*d* = −0.26; *P = *2.04 × 10^−5^), compared to healthy controls (Table 3 and Fig. 3A). Supplementary analysis in a non-lesional epilepsy subgroup revealed a similar pattern of cortical thickness differences compared to controls, suggesting that the changes observed in our main analysis were not driven by the inclusion of patients with hippocampal sclerosis or other common lesions (Supplementary Table 7).

**Table 3:** Effect size differences between epilepsy cases and healthy controls (Cohen’s *d*) for the mean thickness of cortical structures, controlling for age, sex and intracranial volume

Structure	Phenotype	Cohen’s *d*	SE	Z score	95% CI	*P-*value	*I*^2^	*N*_80_	Number of controls	Number of cases
Caudal middle frontal gyrus (LH)	MTLE-L	−0.403	0.07	−5.789	−0.538 to −0.2663	7.07 x 10^−9^	13.807	98	1344	412
	All epilepsies	−0.319	0.04	−7.935	−0.397 to −0.24	2.11 x 10^−15^	17.112	156	1650	2061
	All other epilepsies	−0.291	0.045	−6.425	−0.38 to −0.202	1.32 x 10^−10^	0	197	1447	1000
Caudal middle frontal gyrus (RH)	MTLE-L	−0.441	0.087	−5.089	−0.611 to −0.271	3.61 x 10^−7^	39.444	82	1348	412
	All epilepsies	−0.307	0.051	−5.991	−0.407 to −0.206	2.09 x 10^−9^	46.443	168	1653	2059
	All other epilepsies	−0.212	0.045	−4.699	−0.301 to −0.124	2.62 x 10^−6^	0	350	1451	998
Cuneus (RH)	All other epilepsies	−0.234	0.045	−5.186	−0.323 to −0.146	2.15 x 10^−7^	0	288	1449	996
	All epilepsies	−0.204	0.038	−5.333	−0.279 to −0.129	9.68 x10^−8^	11.423	379	1651	2057
Entorhinal gyrus (LH)	MTLE-L	−0.445	0.072	−6.158	−0.5865 to −0.303	7.35 x 10^−10^	0	81	1102	303
	All epilepsies	−0.264	0.062	−4.261	−0.385 to −0.142	2.04 x 10^−5^	56.648	227	1402	1724
Fusiform gyrus (LH)	MTLE-L	−0.359	0.069	−5.183	−0.494 to −0.223	2.19 x 10^−7^	13.465	123	1339	412
Lateral occipital gyrus (RH)	All other epilepsies	−0.211	0.045	−4.659	−0.299 to −0.122	3.18 x 10^−6^	2.50 x 10^−3^	354	1450	997
Lingual gyrus (RH)	All other epilepsies	−0.180	0.045	−3.972	−0.268 to −0.091	7.12 x 10^−5^	1.25 x 10^−2^	491	1450	996
Paracentral gyrus (LH)	MTLE-R	−0.505	0.102	−4.944	−0.705 to −0.305	7.67 x 10^−7^	52.283	63	1292	338
	MTLE-L	−0.426	0.099	−4.313	−0.62 to −0.232	1.61 x 10^−5^	53.165	88	1344	412
	All epilepsies	−0.311	0.065	−4.748	−0.439 to −0.182	2.05 x 10^−6^	67.476	164	1650	2061
	All other epilepsies	−0.257	0.045	−5.680	−0.346 to −0.168	1.34 x 10^−8^	0	239	1447	1000
Paracentral gyrus (RH)	MTLE-R	−0.421	0.064	−6.538	−0.548 to −0.295	6.24 x 10^−11^	0.407	90	1296	338
	MTLE-L	−0.378	0.075	−5.021	−0.526 to −0.231	5.14 x 10^−7^	23.536	111	1348	412
	All other epilepsies	−0.351	0.045	−7.733	−0.44 to −0.262	1.05 x 10^−14^	3.43 x 10^−3^	129	1451	998
	All epilepsies	−0.315	0.053	−5.983	−0.418 to −0.212	2.19 x 10^−9^	49.261	160	1654	2059
Parahippocampal gyrus (LH)	MTLE-L	−0.3	0.073	−4.11	−0.444 to −0.1572	3.95 x 10^−5^	19.366	176	1335	410
Pars opercularis (RH)	MTLE-R	−0.271	0.071	−3.8	−0.411 to −0.131	1.45 x 10^−4^	12.105	215	1295	338
	All epilepsies	−0.177	0.036	−4.976	−0.247 to −0.107	6.48 x 10^−7^	2.624	503	1652	2059
Pars triangularis (LH)	All epilepsies	−0.192	0.05	−3.828	−0.2897 to −0.094	1.29 x 10^−4^	44.414	427	1650	2060
Pars triangularis (RH)	MTLE-L	−0.285	0.06	−4.738	−0.403 to −0.167	2.16 x 10^−6^	0	195	1346	412
	All epilepsies	−0.199	0.036	−5.48	−0.27 to −0.128	4.25 x 10^−8^	4.66	398	1652	2058
	All other epilepsies	−0.210	0.045	−4.650	−0.299 to −0.122	3.32 x 10^−6^	2.58 x 10^−3^	357	1449	998
Precentral gyrus (LH)	MTLE-L	−0.466	0.081	−5.755	−0.625 to −0.307	8.64 x 10^−9^	31.602	74	1339	412
	MTLE-R	−0.415	0.09	−4.596	−0.592 to −0.238	4.31 x 10^−6^	40.044	93	1287	338
	All epilepsies	−0.384	0.044	−8.768	−0.469 to −0.298	1.82 x 10^−18^	27.649	108	1645	2058
	All other epilepsies	−0.375	0.046	−8.237	−0.464 to −0.286	1.76 x 10^−16^	5.59 x 10^−3^	113	1442	997
	IGE	−0.342	0.071	−4.78	−0.482 to −0.201	1.75 x 10^−6^	0.003	136	1043	297
Precentral gyrus (RH)	MTLE-R	−0.52	0.086	−6.073	−0.687 to −0.352	1.25 x 10^−9^	33.288	60	1293	337
	MTLE-L	−0.492	0.078	−6.335	−0.6436 to −0.339	2.37 x 10^−10^	26.33	66	1345	412
	All epilepsies	−0.399	0.044	−9.102	−0.485 to −0.313	8.85 x 10^−20^	27.929	100	1649	2054
	IGE	−0.39	0.072	−5.442	−0.531 to −0.25	5.27 x 10^−8^	0.005	105	1044	295
	All other epilepsies	−0.348	0.045	−7.672	−0.437 to −0.259	1.70 x 10^−14^	0	131	1448	996
Precuneus (LH)	MTLE-L	−0.536	0.135	−3.965	−0.801 to −0.271	7.35 x 10^−5^	75.18	56	1343	412
	All other epilepsies	−0.178	0.047	−3.819	−0.27 to −0.087	1.34 x 10^−4^	4.474	497	1446	998
Precuneus (RH)	MTLE-L	−0.473	0.104	−4.558	−0.676 to −0.27	5.16 x 10^−6^	57.498	72	1348	412
	All epilepsies	−0.275	0.066	−4.197	−0.404 to −0.147	2.70 x 10^−5^	67.608	209	1654	2055
	All other epilepsies	−0.238	0.053	−4.471	−0.343 to −0.134	7.78 x 10^−6^	22.378	279	1451	994
Superior frontal gyrus (LH)	MTLE-L	−0.411	0.06	−6.804	−0.529 to −0.292	1.02 x 10^−11^	0	94	1343	412
	All epilepsies	−0.283	0.054	−5.251	−0.389 to −0.177	1.51 x 10^−7^	51.773	197	1649	2059
	All other epilepsies	−0.243	0.059	−4.138	−0.358 to −0.128	3.51 x 10^−5^	34.545	267	1446	999
Superior frontal gyrus (RH)	MTLE-L	−0.365	0.06	−6.051	−0.483 to −0.246	1.44 x 10^−9^	0	119	1345	412
	All epilepsies	−0.269	0.059	−4.588	−0.385 to −0.154	4.49 x 10^−6^	59.483	218	1650	2058
	All other epilepsies	−0.235	0.052	−4.489	−0.337 to −0.132	7.15 x 10^−6^	20.049	286	1448	997
Superior parietal gyrus (LH)	All other epilepsies	−0.224	0.045	−4.954	−0.313 to −0.136	7.27 x 10^−7^	0.001	314	1444	996
Superior parietal gyrus (RH)	All other epilepsies	−0.220	0.045	−4.864	−0.309 to −0.131	1.15 x 10^−6^	0.002	326	1450	997
Supramarginal gyrus (LH)	All epilepsies	−0.232	0.06	−3.894	−0.348 to −0.115	9.87 x 10^−5^	59.391	293	1606	1965
Supramarginal gyrus (RH)	All epilepsies	−0.223	0.055	−4.045	−0.331 to −0.115	5.24 x 10^−5^	52.895	317	1597	1971
	All other epilepsies	−0.206	0.047	−4.418	−0.297 to −0.115	9.95 x 10^−6^	0	371	1395	961
Temporal pole (LH)	MTLE-L	−0.315	0.068	−4.649	−0.447 to −0.182	3.33 x 10^−6^	10.901	160	1341	410
Transverse temporal gyrus (LH)	MTLE-R	−0.312	0.073	−4.249	−0.456 to −0.168	2.15 x 10^−5^	15.614	163	1289	338
	All epilepsies	−0.192	0.044	−4.406	−0.278 to −0.107	1.05 x 10^−5^	28.178	427	1647	2061
Transverse temporal gyrus (RH)	All epilepsies	−0.182	0.044	−4.188	−0.267 to −0.097	2.81 x 10^−5^	27.918	475	1654	2059
	All other epilepsies	−0.18	0.045	−3.982	−0.269 to −0.091	6.84 x 10^−5^	0.012	486	1451	998

CI = confidence interval; LH = left hemisphere; RH = right hemisphere; SE = standard error; *I*^2 ^= heterogeneity index; *N*_80_ = number of subjects required in each group to yield 80% power to detect significant group differences (*P* < 0.05, two-tailed). Uncorrected *P*-values are reported. Cortical regions that failed to survive Bonferroni correction (*P* < 1.49 x 10^−4^) are not reported (see ‘Materials and methods’ section for statistical threshold determination). See Supplementary material for a full list of cortical differences with adjustment for false discovery rate (FDR).

The MTLE-L and MTLE-R subgroups showed distinct patterns of cortical thickness reductions when compared to healthy controls (Table 3, Fig. 3B and C). In MTLE-R, lower cortical thickness was reported across four motor regions, including the left (*d* = −0.51; *P = *7.67 × 10^−7^) and right paracentral gyri (*d* = −0.42; *P = *6.24 × 10^−11^), and the left (*d* = −0.42; *P = *4.31 × 10^−6^) and right precentral gyri (*d* = −0.52; *P = *1.25 × 10^−9^). The MTLE-R subgroup also showed thickness changes in the left transverse temporal gyrus (*d* = −0.31; *P = *2.15 × 10^−5^), and right pars opercularis (*d* = −0.27; *P = *1.45 × 10^−4^) (Table 3 and Fig. 3C). By contrast, in MTLE-L, lower thickness was observed across six regions of the motor cortex, including the left (*d* = −0.43; *P = *1.61 × 10^−5^) and right paracentral gyri (*d* = −0.38; *P = *5.14 × 10^−7^), left (*d* = −0.47; *P = *8.64 × 10^−9^) and right precentral gyri (*d* = −0.49; *P = *2.37 × 10^−10^), and left (*d* = −0.54; *P = *7.35 × 10^−5^) and right precuneus (*d* = −0.47; *P = *5.16 × 10^−6^). The MTLE-L group also showed thickness changes across five regions of the frontal cortex, including the left (*d* = −0.41; *P = *1.02 × 10^−11^) and right superior frontal gyri (*d* = −0.37; *P = *1.44 × 10^−9^), left (*d* = −0.4; *P = *7.07 × 10^−9^) and right caudal middle frontal gyri (*d* = −0.44; *P = *3.61 × 10^−7^), and the right *pars triangularis* (*d* = −0.29; *P = *2.16 × 10^−6^). In MTLE-L, thickness alterations were also observed in four regions of the temporal cortex, including the left temporopolar cortex (*d* = −0.32; *P = *3.33 × 10^−6^), left parahippocampal gyrus (*d* = −0.3; *P = *3.95 × 10^−5^), left entorhinal gyrus (*d* = −0.45; *P = *7.35 × 10^−10^), and left fusiform gyrus (*d* = −0.36; *P = *2.19 × 10^−7^) (Table 3 and Fig. 3B).

The IGE subgroup showed reduced thickness in the left (*d* = −0.34; *P = *1.75 × 10^−6^) and right precentral gyri (*d* = −0.39; *P = *5.27 × 10^−8^), when compared to healthy controls (Table 3 and Fig. 3D).

The all-other-epilepsies subgroup showed lower thickness across six cortical regions bilaterally, including the left (*d* = −0.38; *P = *1.76 × 10^−16^) and right precentral gyri (*d* = −0.35; *P = *1.7 × 10^−14^), left (*d* = −0.26; *P = *1.34 × 10^−8^) and right paracentral gyri (*d* = −0.35; *P = *1.1 × 10^−14^), left (*d* = −0.29; *P = *1.32 × 10^−10^) and right caudal middle frontal gyri (*d* = −0.21; *P = *2.62 × 10^−6^), left (*d* = −0.22; *P = *7.27 × 10^−7^) and right superior parietal gyri (*d* = −0.22; *P = *1.15 × 10^−6^), left (*d* = −0.24; *P = *3.51 × 10^−5^) and right superior frontal gyri (*d* = −0.23; *P = *7.15 × 10^−6^), and the left (*d* = −0.18; *P = *1.34 × 10^−4^) and right precuneus (*d* = −0.24; *P = *7.78 × 10^−6^) compared to controls. The all-other-epilepsies group also showed unilaterally reduced thickness in six right hemispheric regions, including the cuneus (*d* = −0.23; *P = *2.15 × 10^−7^), lateral occipital gyrus (*d* = −0.21; *P = *3.18 × 10^−6^), pars triangularis (*d* = −0.21; *P = *3.32 × 10^−6^), supramarginal gyrus (*d* = −0.21; *P = *9.95 × 10^−6^), transverse temporal gyrus (*d* = −0.18; *P = *6.84 × 10^−5^), and lingual gyrus (*d* = −0.18; *P = *7.12 × 10^−5^), compared to controls (Table 3 and Fig. 3E).

An interactive 3D visualization of these results is available via the ENIGMA-Viewer tool (Zhang *et al.*, 2017), at http://enigma-viewer.org/ENIGMA_epilepsy_cortical.html (Supplementary material). Cortical thickness differences significant after FDR adjustment can also be visualized at http://enigma-viewer.org/ENIGMA_epilepsy_cortical_fdr.html (Supplementary Tables 31–35).

### Duration of illness, age at onset, and age-by-diagnosis effects on brain abnormalities

A secondary analysis identified significant associations between duration of epilepsy and several affected brain regions in the all-epilepsies, MTLE-R, and all-other-epilepsies groups. In the all-epilepsies group, duration of epilepsy negatively associated with volume measures in the left hippocampus (*b* = −8.32; *P* = 8.16 × 10^−13^), left (*b* = −13.58; *P* = 3.52 × 10^−15^), and right thalamus (*b* = −12.25; *P* = 1.58 × 10^−13^), and right pallidum (*b* = −2.67; *P* = 1.78 × 10^−7^), in addition to bilateral thickness measures in the left (*b* = −0.003; *P* = 2.99 × 10^−11^) and right pars triangularis (*b* = −0.002; *P* = 4.24 × 10^−9^), left (*b* = −0.003; *P* = 1.61 × 10^−15^) and right caudal middle frontal gyri (*b* = −0.003; *P* = 1.65 × 10^−17^), left (*b* = −0.003; *P* = 1.77 × 10^−13^) and right supramarginal gyri (*b* = −0.003; *P* = 2.58 × 10^−19^), left (*b* = −0.003; *P* = 5.84 × 10^− 12^) and right precentral gyri (*b* = −0.003; *P* = 2.54 × 10^−24^), left (*b* = −0.004; *P* = 1.94 × 10^−12^) and right superior frontal gyri (*b* = −0.003; *P* = 4.65 × 10^−11^), left (*b* = −0.004; *P* = 1.05 × 10^−10^) and right transverse temporal gyri (*b* = −0.003; *P* = 8.24 × 10^−10^), and left (*b* = −0.002; *P* = 5.22 × 10^−6^) and right paracentral gyri (*b* = −0.002; *P* = 5.63 × 10^−6^). Duration of epilepsy also negatively associated with unilateral thickness measures in the right precuneus (*b* = −0.003; *P* = 6.03 × 10^−21^), right pars opercularis (*b* = −0.003; *P* = 5.59 × 10^−13^), and right cuneus (*b* = −0.002; *P* = 1.1 × 10^−9^; Supplementary Table 8). In the MTLE-R subgroup, duration of epilepsy negatively associated with volume measures in the right hippocampus (*b* = −22.42; *P* = 1.1 × 10^−7^), and the right thalamus (*b* = −18.11; *P* = 1.84 × 10^−5^), and thickness measures in the left transverse temporal gyrus (*b* = −0.007; *P* = 8.39 × 10^−5^; Supplementary Table 8). In the all-other-epilepsies subgroup, duration of epilepsy negatively associated with bilateral thickness measures in the left (*b* = −0.003; *P* = 3.39 × 10^−7^) and right caudal middle frontal gyri (*b* = −0.003; *P* = 6.91 × 10^−8^), left (*b* = −0.003; *P* = 1.36 × 10^−9^) and right superior frontal gyri (*b* = −0.003; *P* = 3.16 × 10^−7^), and the left (*b* = −0.003; *P* = 3.17 × 10^−5^) and right precuneus (*b* = −0.003; *P* = 5.01 × 10^−9^), in addition to unilateral thickness measures in the right precentral gyrus (*b* = −0.004; *P* = 1.16 × 10^−12^), right cuneus (*b* = −0.003; *P* = 8.57 × 10^−8^), right pars triangularis (*b* = −0.003; *P* = 5.16 × 10^−7^), and right supramarginal gyrus (*b* = −0.003; *P* = 2.24 × 10^−7^). Duration of epilepsy also showed a positive association with the size of the left lateral ventricle in the all-other-epilepsies group (*b* = 13.6; *P* = 1.17 × 10^−5^).

In the all-epilepsies group, age at onset of epilepsy negatively associated with thickness measures in the left (*b* = −0.003; *P* = 2.66 × 10^−15^) and right superior frontal gyri (*b* = −0.003; *P* = 9.77 × 10^−10^), left (*b* = −0.003; *P* = 2.78 × 10^−9^) and right pars triangularis (*b* = −0.003; *P* = 6.51 × 10^−7^), right pars opercularis (*b* = −0.003; *P* = 5.4 × 10^−14^), left transverse temporal gyrus (*b* = −0.003; *P* = 1.03 × 10^−8^), and right cuneus (*b* = −0.001; *P* = 4.9 × 10^−6^). In the all-other-epilepsies subgroup, age at onset negatively correlated with thickness measures in the left (*b* = −0.003; *P* = 3.21 × 10^−8^) and right superior frontal gyri (*b* = −0.002; *P* = 1.18 × 10^−4^), left (*b* = −0.002; *P* = 8.42 × 10^−6^) and right precuneus (*b* = −0.002; *P* = 7.23 × 10^−5^), right pars triangularis (*b* = −0.003; *P* = 2.53 × 10^−5^), and right supramarginal gyrus (*b* = −0.002; *P* = 2.38 × 10^−6^). Age at onset also positively associated with the size of the right lateral ventricle in the all-other-epilepsies subgroup (*b* = 57.73; *P* = 1.62 × 10^−7^).

Age at onset negatively associated with other regional volumetric and thickness measures in the all-epilepsies, IGE, MTLE-L, MTLE-R, and all-other-epilepsies groups, but these associated areas showed no significant structural differences in the primary case-control analysis (Table 1 and Supplementary Table 8).

There were no interaction effects between age and syndromic diagnosis in the all-epilepsies, MTLE-L, MTLE-R, IGE, or all-other-epilepsies groups.

### Power analyses for detection of case-control differences

In our sample of 2149 individuals with epilepsy and 1727 healthy controls, we had 80% power to detect Cohen’s *d* effect sizes as small as *d* = 0.091 at the standard alpha level of *P* < 0.05 (two-tailed), and 80% power to detect Cohen’s *d* effect sizes as small as *d* = 0.149 at the study’s stringent Bonferroni-corrected threshold of *P* < 1.49 × 10^−4^.

*N_80_*, the number of cases and controls required to achieve 80% power to detect group differences using a two-tailed *t*-test at *P* < 0.05, ranged from *N*_80_ = 6, to detect group effects in the right hippocampus in our MTLE-R group, to *N*_80_ = 503, to detect group effects in the right pars opercularis in our ‘all epilepsies’ group (Tables 2 and 3).

## Discussion

In the largest coordinated neuroimaging study of epilepsy to date, we identified a series of quantitative imaging signatures—some shared across common epilepsy syndromes, and others characteristic of selected, specific epilepsy syndromes. Our sample of 2149 individuals with epilepsy and 1727 controls provided 80% power to detect differences as small as *d* = 0.091 (*P* < 0.05, two-tailed), allowing us to identify subtle, consistent brain abnormalities that are typically undetectable on visual inspection, or overlooked using smaller case-control designs. This international collaboration addresses prior inconsistencies in the field of epilepsy neuroimaging, providing a robust, *in vivo* map of structural aberrations, upon which future studies of disease mechanisms may expand.

In the first of five cross-sectional MRI analyses, we investigated a diverse aggregation of epilepsy syndromes, putative causes, and durations of disease. This all-epilepsies group exhibited shared, diffuse brain structural differences across several regions including the thalamus, pallidum, precentral, paracentral, and superior frontal cortices. With the exception of hippocampal volume and entorhinal thickness differences (Supplementary material), these structural alterations were not driven by any specific syndrome or dataset (Supplementary Figs 3 and 7). Our findings suggest a common neuroanatomical signature of epilepsy across a wide spectrum of disease types, complementing recent evidence for shared genetic susceptibility to a wide spectrum of epilepsies (International League Against Epilepsy Consortium on Complex Epilepsies, 2014). Some structural and genetic pathways may be shared across syndromes, despite the heterogeneity of epilepsy and seizure types. This shared MRI signature underpins the contemporary shift towards the study of epilepsies as network phenomena (Caciagli *et al.*, 2014).

In MTLE, as expected, we observed hippocampal volume abnormalities ipsilateral to the patient’s side of seizure onset. Neither MTLE-L nor MTLE-R showed significant contralateral hippocampal volume reductions, confirming that sporadic, unilateral MTLE is not routinely underpinned by bilateral hippocampal damage (Blümcke *et al.*, 2013). Both MTLE groups showed extrahippocampal abnormalities in the ipsilateral thalamus and pallidum, with widespread reductions in cortical thickness, supporting a growing body of literature indicating that MTLE, as an example of a specific disease constellation in the epilepsies, is also a network disease, extending beyond the mesial temporal regions (Keller *et al.*, 2014; de Campos *et al.*, 2016). Disruption of this network, notably in the thalamus (Keller *et al.*, 2015; He *et al.*, 2017) and thalamo-temporal white matter tracts (Keller *et al.*, 2015, 2017), may be associated with postoperative seizure outcome in MTLE.

Patients with left and right MTLE showed distinct patterns of structural abnormalities when compared to controls, resolving conflicting findings from smaller studies, some reporting an equal distribution of structural differences (Liu *et al.*, 2016), and others indicating more diffuse abnormalities, either in left MTLE (Keller *et al.*, 2002, 2012; Bonilha *et al.*, 2007; Kemmotsu *et al.*, 2011; de Campos *et al.*, 2016) or in right MTLE (Pail *et al.*, 2009). The structural differences observed in the present study may reflect a younger age at onset of epilepsy in left MTLE, which occurred, on average, 1.2 years earlier than those with right MTLE (Supplementary Table 20). Independent, large-scale studies of MTLE patients have confirmed a significantly earlier age at onset in left, compared to right, MTLE (Blümcke *et al.*, 2017). Duration-related effects were also observed in right, but not left, MTLE, pointing to possible biological distinctions between the two.

In IGE, a clinically and biologically distinct group of epilepsies typically associated with ‘normal’ MRI on clinical inspection (Woermann *et al.*, 1998), we identified reduced volume of the right thalamus, and thinner precentral gyri in both hemispheres, supporting prior reports of structural (Bernhardt *et al.*, 2009*a*), electroencephalographic, and functional (Gotman *et al.*, 2005) abnormalities in IGE. These IGE cases were considered typical by reviewing neurologists, suggesting that this common type of epilepsy is also associated with quantifiable structural brain abnormalities.

The precentral gyri, site of the primary motor cortex, showed bilateral structural deficits across all epilepsy groups (all-epilepsies, IGE, MTLE-L, MTLE-R, and all-other-epilepsies), without detectable inter-cohort or between-disease heterogeneity (Supplementary Figs 3–12). Atrophy of the motor cortex has been linked to seizure frequency and duration of epilepsy in MTLE (Coan *et al.*, 2014); here, we observed a negative correlation between precentral (and postcentral) grey matter thickness and duration of epilepsy in the aggregate all-epilepsies group.

The right thalamus also showed evidence of structural compromise across all epilepsy cohorts, re-emphasizing the importance of the thalamus as a major hub in the epilepsy network (He *et al.*, 2017; Jobst and Cascino, 2017). Loss of feed-forward inhibition between the thalamus and its neocortical connections may be epileptogenic (Paz and Huguenard, 2015), and thalamocortical abnormalities have previously been reported in IGE (Gotman *et al.*, 2005; Bernhardt *et al.*, 2009*a*; O’Muircheartaigh *et al.*, 2012) and MTLE (Mueller *et al.*, 2010; Bernhardt *et al.*, 2012). These findings support prior ‘system epilepsies’ hypotheses of pathophysiology (Avanzini *et al.*, 2012), suggesting that a broad range of common epilepsies share vulnerability within a thalamocortical structural pathway involved in, and likely affected by, seizures (Liu *et al.*, 2003; Bernhardt *et al.*, 2013). Given this study’s cross-sectional design, we cannot determine if these are causative changes, consequences of recurrent seizures, prolonged drug treatment, or a combination of factors. The epilepsies, as a broad group, may involve progressive structural change (Caciagli *et al.*, 2017), indicating the need for large-scale longitudinal studies.

A heterogeneous subgroup of individuals without confirmed diagnoses of IGE or MTLE with hippocampal sclerosis showed similar patterns of structural alterations to those observed in the aggregate all-epilepsies cohort. The findings included enlarged ventricles, smaller right pallidum and right thalamus, and reduced thickness across the motor and frontal cortices. Hippocampal abnormalities were not observed in this subgroup, suggesting that the patterns of reduced hippocampal grey matter observed in the aggregate group were driven by the inclusion of MTLEs with hippocampal sclerosis. Unlike the IGE, MTLE, and aggregate epilepsy cohorts, this subgroup also showed bilateral enlargement of the amygdala—a phenomenon previously reported in non-lesional localization-related epilepsies (Reyes *et al.*, 2017) and non-lesional MTLEs (Takaya *et al.*, 2012; Coan *et al.*, 2013). Non-lesional MTLEs formed a large proportion of this ‘all-other-epilepsies’ cohort (43.3%; 445 individuals), but the subgroup included many other focal and unclassified syndromes, potentially obscuring specific biological interpretations. Future, sufficiently powered studies will stratify this cohort into finer-grained subtypes to delineate syndrome-specific effects.

Despite its international scale, our study has limitations. All results were derived from cross-sectional data: we cannot distinguish between historical acute damage and progressive abnormalities. We cannot disentangle the relative contributions of environmental and treatment-related factors, including antiepileptic medications, seizure types and frequencies, disease severity, language dominance, and other initial precipitating factors. On average, duration of epilepsy was at least 10 years; longitudinal investigations of new-onset and paediatric epilepsies will provide a more comprehensive understanding. Despite using standardized image processing protocols, quality control, and statistical techniques, some brain measures showed a wide distribution of effect sizes across research centres, which may reflect sample heterogeneity and differences in scanning protocols (Supplementary material).

We observed modest thickness differences across the majority of cortical regions; Cohen’s *d* effect sizes ranged from small to moderate (*d* = 0.2–0.5), with some very small effects (*d* < 0.2) noted in the right pars opercularis, bilateral pars triangularis, and bilateral transverse temporal gyri of the aggregate all-epilepsies group. Other large-scale ENIGMA studies have reported similarly modest (albeit less widespread) cortical abnormalities in psychiatric illnesses including major depression (Schmaal *et al.*, 2016) and bipolar disorder (Hibar *et al.*, 2017*b*). Although epilepsy is characterized by an enduring predisposition to generate abnormal excessive or synchronous neuronal activity in the brain (Fisher *et al.*, 2014), our findings indicate that common epilepsies are associated with widespread, but relatively subtle, structural alterations of the neocortex. Replication in independent MRI cohorts, complemented by advanced imaging modalities and large-scale gene expression datasets, will help elucidate how these cortical abnormalities relate to underlying disease processes.

Overall, in the largest neuroimaging analysis of epilepsy to date, we demonstrate a pattern of robust brain structural abnormalities within and between syndromes. Specific functional interpretations cannot be inferred from grey matter differences, but lower volume and thickness measures may reflect tissue loss, supporting recent observations that the common epilepsies cannot always be considered benign (Gaitatzis *et al.*, 2004; Bell *et al.*, 2016; Devinsky *et al.*, 2017). The study provides a macroscopic neuroanatomical map upon which neuropathological work, animal models, and further gene expression studies, can expand. Our consortium plans to investigate more specific neuroanatomical traits and epilepsy phenotypes, explore sophisticated shape and sulcal measures, and eventually conduct genome-wide association analysis of brain measures, to improve our understanding and treatment of the epilepsies.

## Web resources

All image processing, quality assurance, and statistical analysis protocols for this study can be downloaded from the ENIGMA website, at: http://enigma.usc.edu/ongoing/enigma-epilepsy/enigma-epilepsy-protocols/.

## Supplementary Material

Supplementary Tables and Figures

## Abbreviations

ENIGMA: Enhancing Neuro Imaging Genetics through Meta-Analysis
IGE: idiopathic generalized epilepsy
MTLE-L/R: mesial temporal lobe epilepsy with left/right hippocampal sclerosis
