# Supplementary material for: Structural brain abnormalities in the common epilepsies assessed in a worldwide ENIGMA study
Source: Brain. 2018 Jan 30;141(2):391–408. doi: 10.1093/brain/awx341 (PMC5837616; doi:10.1093/brain/awx341)
Supplement: Supplementary Tables and Figures [file brain-2017-01077-file006_awx341.pdf]

Supplementary Information

Structural brain abnormalities in the common epilepsies assessed in 3,876 individuals worldwide

Supplementary Note 1. Additional details of recruitment, standardized image inspection and quality control.

Supplementary Note 2. Description of the coordinated ENIGMA meta-analysis framework.

Supplementary Note 3. Supplementary analysis of 'all-non-lesional' epilepsies versus controls

Supplementary Note 4. Analysis of data acquired at 3T field strength only.

Supplementary Note 5. ENIGMA results viewer: Instructions for use

Supplementary Note 6. Additional supplementary materials.

Supplementary Table 1. Locations, dates and periods of participant recruitment, including research ethics committee (REC) details, listed by site.

Supplementary Table 2. Breakdown of syndromic diagnoses in the 'all-epilepsies' aggregate phenotype group.

Supplementary Table 3. Image acquisition and processing details by site.

Supplementary Table 4. Full list of cortical regions of interest, as extracted using FreeSurfer v5.3.0.

Supplementary Table 5. Results of t-tests for age differences and chi-squared tests for sex differences at each research centre.

Supplementary Table 6. Effect sizes for volume differences (Cohen's *d*) between healthy controls and a subgroup of the 'all-epilepsies' phenotype, without evidence of hippocampal sclerosis or other lesions on MRI, controlling for age, sex, and intracranial volume.

Supplementary Table 7. Effect sizes for cortical thickness differences (Cohen's *d*) between healthy controls and a subgroup of the 'all-epilepsies' phenotype, without evidence of hippocampal sclerosis or other lesions on MRI, controlling for age, sex, and intracranial volume.

Supplementary Table 8. Beta coefficients, standard errors and p-values for the meta-regression of duration of illness in epilepsy against subcortical volume and cortical thickness, controlling for sex and intracranial volume.

Supplementary Table 9. Beta coefficients, standard errors and p-values for the meta-regression of age at onset of epilepsy against subcortical volume and cortical thickness, controlling for sex and intracranial volume.

Supplementary Table 10. Effect sizes for volume differences (Cohen's *d*) between healthy controls and the 'all-epilepsies' phenotype; 3T field strength only.

Supplementary Table 11. Effect sizes for volume differences (Cohen's *d*) between healthy controls and the 'MTLE-L' phenotype; 3T field strength only.

Supplementary Table 12. Effect sizes for volume differences (Cohen's *d*) between healthy controls and the 'MTLE-R' phenotype; 3T field strength only.

Supplementary Table 13. Effect sizes for volume differences (Cohen's *d*) between healthy controls and the 'IGE' phenotype; 3T field strength only.

Supplementary Table 14. Effect sizes for volume differences (Cohen's *d*) between healthy controls and the 'all-other-epilepsies' phenotype; 3T field strength only.

Supplementary Table 15. Effect sizes for cortical thickness differences (Cohen's *d*) between healthy controls and the 'all-epilepsies' phenotype; 3T field strength only.

Supplementary Table 16. Effect sizes for cortical thickness differences (Cohen's *d*) between healthy controls and the 'MTLE-L' phenotype; 3T field strength only.

Supplementary Table 17. Effect sizes for cortical thickness differences (Cohen's *d*) between healthy controls and the 'MTLE-R' phenotype; 3T field strength only.

Supplementary Table 18. Effect sizes for cortical thickness differences (Cohen's *d*) between healthy controls and the 'IGE' phenotype; 3T field strength only.

Supplementary Table 19. Effect sizes for cortical thickness differences (Cohen's *d*) between healthy controls and the 'all-other-epilepsies' phenotype; 3T field strength only.

Supplementary Table 20. Average ages at onset of epilepsy per research centre.

Supplementary Table 21. Full meta-analytical results of the age-by-diagnosis interaction test, controlling for sex and ICV| 'All epilepsies' phenotype

Supplementary Table 22. Full meta-analytical results of the age-by-diagnosis interaction test, controlling for sex and ICV| 'MTLE-L' phenotype

Supplementary Table 23. Full meta-analytical results of the age-by-diagnosis interaction test, controlling for sex and ICV| 'MTLE-R' phenotype

Supplementary Table 24. Full meta-analytical results of the age-by-diagnosis interaction test, controlling for sex and ICV| 'IGE' phenotype

Supplementary Table 25. Full meta-analytical results of the age-by-diagnosis interaction test, controlling for sex and ICV| 'All other epilepsies' phenotype

Supplementary Table 26. Full list of case-versus-control effect sizes for volume differences, including non-significant findings | 'All epilepsies' phenotype

Supplementary Table 27. Full list of case-versus-control effect sizes for volume differences, including non-significant findings | 'MTLE-L' phenotype

Supplementary Table 28. Full list of case-versus-control effect sizes for volume differences, including non-significant findings | 'MTLE-R' phenotype

Supplementary Table 29. Full list of case-versus-control effect sizes for volume differences, including non-significant findings | 'IGE' phenotype

Supplementary Table 30. Full list of case-versus-control effect sizes for volume differences, including non-significant findings | 'All other epilepsies' phenotype

Supplementary Table 31. Full list of case-versus-control effect sizes for cortical thickness differences, including non-significant findings | 'All epilepsies' phenotype

Supplementary Table 32. Full list of case-versus-control effect sizes for cortical thickness differences, including non-significant findings | 'MTLE-L' phenotype

Supplementary Table 33. Full list of case-versus-control effect sizes for cortical thickness differences, including non-significant findings | 'MTLE-R' phenotype

Supplementary Table 34. Full list of case-versus-control effect sizes for cortical thickness differences, including non-significant findings | 'IGE' phenotype

Supplementary Table 35. Full list of case-versus-control effect sizes for cortical thickness differences, including non-significant findings | 'All other epilepsies' phenotype

Supplementary Figure 1. ENIGMA-Epilepsy worldwide map.

Supplementary Figure 2. Illustration of the coordinated ENIGMA meta-analysis framework.

Supplementary Figures 3-12. Forest plots (included in a separate document; see Supplementary Note 6)

Supplementary Note 1: Additional details of recruitment, standardized image inspection, and quality control

Data were collected at 21 unique research institutions worldwide. Scans were acquired at two separate research centres in Dublin, Ireland (see: EPIGEN_1.5T and EPIGEN_3.0T), Tübingen, Germany (see: EKUT_A and EKUT_B), and King's College London (see: KCL_CNS and KCL_CRF). We set an arbitrary minimum sample size of n=5 for all epilepsy groups. Research centers containing epilepsy groups with less than 5 participants were omitted from that analysis. For example, the Brussels center, which consisted of N=4 individuals with MTLE-R, was omitted from all MTLE-R regressions. Similarly, the IDIBAPS group, which contained N=3 IGE cases, was not included in any IGE regressions. In Table 1, sites with less than 5 participants are marked as '0' for that phenotype, with the original sample size noted in brackets '()'. Age at onset and duration of illness information were not available for the **Bern** and **EPICZ** sites.

We did not harmonize image acquisition parameters across research centres, as the vast majority of T1-weighted MRI scans were collected before this study commenced (see Table 1). However, all other aspects of the study, from image processing, to quality inspection, to statistical analysis, were conducted in a coordinated manner across centres, using standardized, well-published protocols, freely available at http://enigma.usc.edu. Analysts at each research centre were blinded to the participant's diagnosis. All analysts conducted image processing using the latest version (v5.3.0) of FreeSurfer – an automated suite of brain MRI reconstruction tools (Fischl et al., 2012). Analysts examined each image individually for improper segmentation. An online ENIGMA support group provided neuroanatomical expertise in cases of uncertain segmentation. Quality assurance guidelines and videos were also made available on the ENIGMA website. Further, each analyst was instructed to execute an automated ENIGMA script, which provided a list of cortical thickness values that deviated from their centre's mean at > 3 deviations. Any segmentation identified as an outlier via this script was re-assessed via visual inspection. Structures that the analyst judged as poorly segmented were omitted from the analysis (the full brain scan was not typically omitted, unless segmentation errors were markedly widespread). Structures identified as statistical outliers, which the analyst judged as correctly segmented, were kept in the analysis. Histogram plots were generated for each cortical thickness measure: these showed an approximately normal distribution of brain measures, at each research centre. Histogram plots for each research centre are available to download and view at http://enigma.ini.usc.edu/ongoing/enigma-epilepsy/enigma-epilepsy-gm/.

Supplementary Note 2: Description of the coordinated ENIGMA meta-analysis framework

All statistical analyses were conducted separately at each research centre, using ENIGMA's standardized statistical analysis scripts (see Supplementary Figure 2). These open-source scripts connect the user to an online Google Spreadsheet listing a series of R commands for linear regression and partial correlation. The main study PI can manipulate this list of commands to include new tests, without the need to re-configure scripts to each user. The user simply specifies their research centre ID (e.g. "EPIGEN"), and the paths to their local data and script directories (e.g. "/users/christopherwhelan/ENIGMA-Epilepsy_data/" and "/users/christopherwhelan/ENIGMA-Epilepsy_scripts/"), executes the script in a bash terminal, and receives a collection of output files containing Cohen's *d*

effect size estimates (for case-control linear regressions), *beta* coefficients (for partial correlations), standard errors, p-values, and several other statistical measures. No MRI data or participant information are exchanged between centres, and all output statistics are completely anonymised.

All statistical output files are then uploaded to a central ENIGMA server, and pooled using an inverse variance-weighted random-effects meta-analysis, via the R package, *metafor* (version 1.9-8)[1]. We chose to meta-analyse data using a random-effects model to adjust for the large number of case-control cohorts included in the study (N=24, from 21 research centres), with heterogeneous ethnicities, diagnoses, seizure types, and age ranges. We employed the restricted maximum likelihood method (REML)[2] in all random-effects meta-analyses.

1. Viechtbauer W (2010) Conducting Meta-Analyses in R with the metafor Package. J Stat Softw 36(3):1-48.

2. Harville DA (1977) Maximum Likelihood Approaches to Variance Component Estimation and to Related Problems. J Am Stat Assoc 72(358):320-338.

Supplementary Note 3: Supplementary analysis of non-lesional epilepsies versus controls

To test for a possible confounding effect of known hippocampal sclerosis in the aggregated all-epilepsies phenotype group, we conducted a supplementary case-control regression using a smaller sub-sample of the all-epilepsies aggregate ('all non-lesional epilepsies'). This 'all-non-lesional epilepsies' subgroup overlapped with the aggregated group of syndromic diagnoses listed in Supplementary Table 2; however, patients were excluded from the non-lesional group if they presented with any evidence of abnormalities on clinical MRI, including focal cortical dysplasia, tumours, hippocampal sclerosis or other lesions. The aggregated total number of cases in this group was N=939, and the total number of controls was 1,379.

Each research centre tested for case-versus-control differences using the same coordinated statistical analysis protocols detailed in the Methods. Sex, age and ICV were again included as covariates. *P*-values were compared to an adjusted significance threshold of $P_{thresh}<1.49\text{x}10^{-4}$, as in the main analysis.

Compared to healthy controls, the all-non-lesional epilepsies group revealed patterns of subcortical volume reduction in the right thalamus and right pallidum, in addition to enlargements of the right lateral ventricle (with nominally enlarged left lateral ventricle), largely supporting findings from the aggregate all-epilepsies analysis (see Results). Contrasting with the all-epilepsies analysis, the all-non-lesional epilepsies group did not exhibit volume differences in the hippocampus. The all-non-lesional epilepsies group also showed enlargements of the left amygdala, a finding that was not observed in the full all-epilepsies analysis (see Supplementary Table 6 for a full list of *p*-values and effect sizes).

The all-epilepsies group and its smaller, all-non-lesional epilepsies subgroup showed similar patterns of cortical thinning compared to controls, with both epilepsy groups showing lower thickness estimates in the precentral gyrus (bilaterally), paracentral gyrus (bilaterally), caudal middle frontal gyrus (bilaterally), superior frontal gyrus (bilaterally), right cuneus, precuneus (bilaterally in all-epilepsies; right hemisphere only in non-lesional epilepsies), *pars triangularis* (bilaterally in all-epilepsies; right hemisphere only in non-lesional epilepsies), supramarginal gyrus (bilaterally in all-epilepsies; right hemisphere only in non-lesional epilepsies) and transverse temporal gyrus (bilaterally in all-epilepsies; right hemisphere only in non-lesional epilepsies) compared to controls. In the non-lesional subgroup, additional cortical thinning was detected in the right lingual gyrus, the superior parietal cortex bilaterally, and the right lateral occipital cortex. Contrasting with the main all-epilepsies group, the 'all-non-lesional epilepsies' subgroup did not show significant thickness differences in the left entorhinal gyrus (see Supplementary Table 7 for a full list of *p*-values and effect sizes).

Supplementary Note 4: Analysis of data acquired at 3T field strength only

In our main case-control meta-analysis, we tested for volume and thickness differences across 24 research centres, using data extracted at both 1.5T (2 centers; a combined total of 78 people with epilepsy and 68 healthy controls) and 3T field strengths (22 centers; a combined total of 2,071 people with epilepsy and 1,659 healthy controls; see Supplementary Table 3 for a full list of field strengths at each centre).

To test for a possible confounding effect of combined magnetic field strengths, we conducted a second meta-analysis, using data extracted at 3T only. Statistical methods were identical to those detailed in the Methods.

Meta-analytical results obtained using the full sample (1.5T and 3T) and the 3T sample were highly comparable, with similar patterns of cortical thinning reported across all four subgroups and the aggregate 'all-epilepsies' group (see Tables 2-3 and Supplementary Tables 10-19). Differences between the '3T-only' analysis and the full analysis included the following:

(i) In the full analysis of 'all-epilepsies' versus controls, volume differences were reported in the left putamen. These differences were nominally significant in the 3T sample, after Bonferroni correction for multiple comparisons,
(ii) In the full analysis of MTLE versus controls, volume differences were reported in the right thalamus. These differences did not survive Bonferroni correction in the 3T-only analysis,
(iii) In the 3T analysis of individuals with IGE versus controls, volume differences were reported in the right pallidum. These differences did not survive Bonferroni correction in the full analysis,
(iv) In the full 'all other epilepsies' analysis, significant enlargement of the right amygdala was reported. This enlargement did not survive Bonferroni correction in the 3T-only analysis,

(v) In the 3T only analysis of 'MTLE-L' versus controls, thickness differences were reported in the right supramarginal gyrus and right rostral middle frontal gyrus; these differences did not survive Bonferroni correction in the full analysis,

(vi) In the 3T only analysis of 'MTLE-R' versus controls, thickness differences were reported in the left supramarginal, right caudal middle frontal, and right transverse temporal gyri; these differences did not survive Bonferroni correction in the full analysis,

(vii) In the full analysis of 'MTLE-R' versus controls, thickness differences were reported in the right pars opercularis; these differences did not survive Bonferroni correction in the 3T only analysis (see Supplementary Tables 10-19), and

(viii) In the 3T-only analysis of 'all other epilepsies', thickness differences were reported in the right rostral middle frontal gyrus; these did not survive Bonferroni correction for multiple comparisons in the full analysis.

All results that were reported as significant in the full analysis, but did not survive Bonferroni correction in the 3T analysis, fell only slightly below the Bonferroni significance threshold in the 3T analysis ($6x10^{-4} < p < 1.49x10^{-4}$). Thus, the marginally different findings observed at 3T field strength can likely be attributed to the lower sample size of the 3T meta-analysis, compared with the full (3T + 1.5T) meta-analysis.

Supplementary Note 5. ENIGMA results viewer: Instructions for use

All regional effect sizes were visualized in a web-based ENIGMA-Viewer (http://enigma-viewer.org). The interactive viewer can be loaded in a webGL-based browser, e.g., Google Chrome (google.com/chrome). The viewer contains a three-dimensional (3D) spatial view and two-dimensional (2D) multiple bar chart views. The bar chart view displays the tables of regional effect sizes. The bars are color coded using a cold-warm color scale to indicate the effect size values. The shared ROI bars across neighboring charts are connected through curves between each bar, which allows users to quickly identify shared ROIs being analyzed. Clicking an ROI in the bar chart view will toggle its 3D representation in the 3D view coded with the same color, allowing users to investigate the spatial relationship among ROIs and their effect sizes. Moving one's mouse over a bar or a 3D region will display the full ROI name and other statistics, such as *p* value, on the tooltip.

Users can access the online user guide (http://enigma-viewer.org/UserGuide.pdf) for further information on the interactive ENIGMA results viewer.

Supplementary Note 6. Additional supplementary materials

To preserve space, additional supplementary materials (including Forest plots, and the full, FDR-corrected results for the secondary regressions of age at onset and duration of illness) are provided via the ENIGMA-Epilepsy website, at: http://enigma.ini.usc.edu/ongoing/enigma-epilepsy/enigma-epilepsy-gm/

Supplementary Table 1. Locations/periods of participant recruitment, and details of research ethics committee approval, at each centre.

Research centre	Location of recruitment	Period of recruitment	Instrument(s) for diagnosis of epilepsy	Research ethics committee (REC) number, if available
**Bern**	Inselspital Universitätsspital **Bern**, Switzerland	2009 – 2015	Review of seizure semiology; ictal/inter-ictal EEG, video-telemetry recordings (where available) and routine MRI	Signed consent forms were acquired to use images for research; no REC number is available
**Bonn**	University Hospital **Bonn**, Germany	July 2006 - December 2011	Review of seizure semiology; ictal/inter-ictal EEG, video-telemetry recordings (where available) and routine MRI	Signed consent forms were acquired to use images for research; no REC number is available
**BRI**	Brain Research Institute (**BRI**), Austin Hospital, Melbourne, Australia	Nov 2007 - Jul 2015	Review of seizure semiology; ictal/inter-ictal EEG, video-telemetry recordings (where available) and routine MRI	H2008/03296, H2012/04475
**Brussels**	Hospital Erasme, Universite ibre de Bruxelles, **Brussels**, Belgium	Aug 2005 - Sept 2015	Review of seizure semiology; ictal/inter-ictal EEG, video-telemetry recordings (where available) and routine MRI	Consent forms were acquired to use images for research; no REC number is available
**CUBRIC**	Cardiff University Brain Research Imaging Centre (**CUBRIC**), Cardiff, United Kingdom	Jun 2009 - May 2015	Review of seizure semiology; ictal/inter-ictal EEG, video-telemetry recordings (where available) and routine MRI	Southmead Research Ethics Committee, Bristol, UK, Rec Ref : 08/H0102/12
**EKUT_A**	Scanning site "A", Eberhard Karls Universität Tübingen (**EKUT_A**), Germany	Feb 2011 – Jan 2016	Review of seizure semiology; ictal/inter-ictal EEG, video-telemetry recordings (where available) and routine MRI	255/2015A
**EKUT_B**	Scanning site "B", Eberhard Karls Universität Tübingen (**EKUT_B**), Germany	Feb 2012 – Nov 2014	Review of seizure semiology; ictal/inter-ictal EEG, video-telemetry recordings (where available) and routine MRI	646/2011BO1
**EPICZ**	Epilepsy Centre Cantazaro (**EPICZ**), Italy	June 2010 - March 2015	Seizure semiology; interictal and ictal video-EEG monitoring; MRI	2010/03/31
**EPIGEN_3.0**	Epilepsy Genetics (**EPIGEN**) Dublin, St. James's Hospital, Dublin, Ireland	September 2010 – September 2014	Review of seizure semiology; ictal/inter-ictal EEG, video-telemetry recordings (where available) and routine MRI	2011/10/01
**EPIGEN_1.5**	Epilepsy Genetics (**EPIGEN**) Dublin, Beaumont Hospital, Dublin, Ireland	September 2008 – September 2012	Review of seizure semiology; ictal/inter-ictal EEG, video-telemetry recordings (where available) and routine MRI	11/97
**Florence**	University of **Florence**, Italy	June 2008 - September 2015	Review of seizure semiology; ictal/inter-ictal EEG, video-telemetry recordings (where available) and routine MRI	2013/5/6
**Greifswald**	Department of Neurology, University Medicine **Greifswald**, Germany	June 2010 - June 2015	Review of seizure semiology; ictal/inter-ictal EEG, video-telemetry recordings (where available) and routine MRI	Signed consent forms were acquired to use images for research; no REC number is available
**IDIBAPS-HCB**	Institut D'Investigacions Biomèdiques August Pi I Sunyer research center, Hospital Clínic Barcelona (**IDIBAPS-HCB**), Cataluña, Spain	January-2007- July 2015	Review of seizure semiology; ictal/inter-ictal EEG, video-telemetry recordings (where available) and routine MRI	Consent forms were acquired to use images for research; no REC number is available
**KCL_CNS**	King's College London Centre for Neuroimaging Sciences (**KCL_CNS**), London, United Kingdom	February 2007 - September 2012	Review of seizure semiology; ictal/inter-ictal EEG, video-telemetry recordings (where available) and routine MRI	08/H0808/157
**KCL_CRF**	King's College London Clinical Research Facility (**KCL_CRF**), London,	July 2013 - May 2015	Review of seizure semiology; ictal/inter-ictal EEG, video-telemetry recordings (where available) and routine MRI	12/LO/2006, 14/LO/0193

	United Kingdom			
**Kuopio**	**Kuopio** University Hospital, Finland	April 2010- August 2015	Review of seizure semiology; ictal/inter-ictal EEG, video-telemetry recordings (where available) and routine MRI	128/13.02.00/2015
**MNI**	Montreal Neurological Institute (**MNI**), McGill University, Montreal, Canada	August 2008 - April 2015	Review of seizure semiology; ictal/inter-ictal EEG, video-telemetry recordings, MRI	NEU-08-001; BERA 2002/1
**NYU**	**New York University**, New York, USA	November 2006-June 2015	Seizure semiology; interictal and ictal video-EEG monitoring; MRI; PET	s12-02038; 11224
**RMH**	Royal Melbourne Hospital (**RMH**), Melbourne, Australia	Jan 2008 -Dec 2013	Review of seizure semiology; ictal/inter-ictal EEG, video-telemetry recordings (where available) and routine MRI, PET (where available)	QA2012044
**UCSD**	University California San Diego (**UCSD**), California, USA	June 2010-September 2015	Seizure semiology; video-EEG telemetry (scalp and FOs; occasional depths) and routine MRI	120297
**UNAM**	Universidad Nacional Autónoma de México (**UNAM**), Campus Juriquilla, Querétaro, México	April 2013-July 2015	Seizure semiology; interictal scalp EEG; routine MRI	019.H-RM
**UNICAMP**	Universidade Estadual de Campinas (**UNICAMP**), Brazil	October 2010 - January 2015	Review of seizure semiology; ictal/inter-ictal EEG, video-telemetry recordings (where available) and routine MRI	CEP1158/2009
**UNIMORE**	Università degli Studi di Modena (**UNIMORE**), Modena, Italy	July 2010-June 2015	Seizure semiology; ictal/inter-ictal EEG, video-telemetry recordings and routine MRI	n. 80/10, n. 155/14
**XMU**	Xiamen University (**XMU**), Xiamen, China	November 2012-December 2015	Seizure semeiology; interictal and ictal video-EEG monitoring; MRI; PET	Signed consent forms were acquired to use images for research; no REC number is available

Supplementary Table 2. Breakdown of syndromic diagnoses in the ‘all-epilepsies’ aggregate phenotype group.

**Syndromic diagnosis**	**%**
**A. Generalised epilepsies**	**17.1**
Idiopathic / genetic generalised epilepsy, not otherwise specified	10.8
Absence epilepsy	2.4
Juvenile myoclonic epilepsy	3.9
**B. Focal – temporal lobe epilepsies**	**55.8**
MTLE-HS-L (mesial temporal lobe epilepsy, MTLE, with left hippocampal sclerosis, HS)	19.3
MTLE-HS-R (MTLE with right HS)	15.8
MTLE without HS, left-sided seizure onset	6.5
MTLE without HS, right-sided seizure onset	4.4
MTLE without HS, bilateral seizure onset	1.0
MTLE without HS, seizure onset side not established	8.8
**C. Focal – occipital, frontal, or parietal lobe epilepsies, or unspecified focal epilepsies**	**23.1**
Occipital lobe epilepsy	0.8
Frontal lobe epilepsy	4.2
Parietal lobe epilepsy	0.4
Unspecified focal epilepsy (Diagnosis of TLE, FLE, OLE or PLE is unclear)	17.7
**D. Unclassified or 'other' epilepsies**	**4.0**

Legend: (A) 19% of individuals in the aggregated ‘all-epilepsies’ phenotype group were diagnosed with a **generalised epilepsy syndrome**. This generalised group consisted of idiopathic generalised epilepsies (IGE) not otherwise specified (13% of the total all-epilepsies sample), childhood absence epilepsies (CAEs, 2%), and juvenile myoclonic epilepsies (JMEs, 4%). **(B) 55%** of individuals in the ‘all-epilepsies’ group had a syndromic diagnosis of **mesial temporal lobe epilepsy (MTLE)**. This MTLE sub-group consisted of MTLEs with left hippocampal sclerosis (HS, 19% of the total sample), MTLEs with right HS (16%), MTLEs without HS and without a specified side of seizure onset (8.8%), MTLEs without HS with left-sided onset (7%), MTLEs without HS with right-sided onset (4%), and MTLEs without HS with bilateral seizure onsets (1%). **(C) 23%** of individuals in the ‘all-epilepsies’ group had a diagnosis of **focal epilepsy other than MTLE**. This included individuals with unspecified forms of focal epilepsy (18% of the total sample), individuals with a diagnosis of frontal lobe epilepsy (FLE, 4%), individuals with a diagnosis of occipital lobe epilepsy (OLE, 0.8%) and individuals with a diagnosis of parietal lobe epilepsy (PLE, 0.4%). **(D) 2.2%** of the ‘all-epilepsies’ group had an unclassified or ‘other’ diagnosis; this included epilepsies whose syndromic diagnoses could not be classified at the time of analysis (3.5% of the total sample), cases of uncertain diagnosis (four cases of tentative IGE versus JME, one case of IGE versus FLE, and one case of FLE versus MTLE), and other syndromes including childhood absence epilepsy with focal cryptogenic epilepsy (one case), unclassified IGE with focal cryptogenic epilepsy of unknown focus (one case), left HS with focal idiopathic epilepsy of unknown focus (one case), left HS with a frontal posttraumatic lesion (one case), and cryptogenic Lennox-Gastaut syndrome with startle epilepsy (one case).

Supplementary Table 3. Image acquisition and processing details by research centre.

Centre	Sequence	Field Strength	Acquisition Direction	# of Slices	Spacing between slices	Voxel Size (mm³)	TI	TE	TR	Flip Angle	Relevant Citation
**Bern**	3D T1-weighted MP-Rage	3 T Siemens Trio	Sagittal	176	0mm	1x1x1mm	900ms	2.01ms	1500ms	9	Held, Fellner et al. (1995). Three-dimensional MP-RAGE – an alternative to conventional three-dimensional FLASH sequences for the diagnosis of viscerocranial tumours? *Br. J. Radiol.* 68: 1316-1324.
**Bonn**	3D T1-weighted magnetization prepared rapid gradient echo (MPRAGE)	3T Trio, Siemens	Coronal	160	0mm	1x1x1mm	650	3.97	1300	10	Keller, S.S. et al. (2015). Morphometric MRI Alterations and Postoperative Seizure Control in Refractory Temporal Lobe Epilepsy. *Human Brain Mapping*, 36:1637-1647.
**BRI**	3D T1-weighted magnetization prepared rapid gradient echo (MPRAGE)	3T TrioTim Siemens	Sagittal	192	0mm	0.9mm, iso	900	2.6	1900	9	Tsai M-H, Pardoe HR, Perchyonok Y, Fitt GJ, Scheffer IE, Jackson GD, Berkovic SF (2013). Etiology of hippocampal sclerosis: evidence for a predisposing familial morphologic anomaly. *Neurology* 81(2),144-9.
**Brussels**	sense 3DT1TFE<br>s3DT1TFE	1.5T Siemens<br>3.0T Siemens	Sagittal<br>Sagittal	130<br>160	0mm<br>0mm	0.87x1.25x1.2mm<br>0.88x1.2x1mm	880<br>1040	4.2<br>4.6	8.8<br>9.8	8<br>8	
**CUBRIC**	3D fast spoiled gradient-recalled (FSPGR)	3T GE HDx	Axial	176	0mm	1x1x1mm	450	3	7.9	20	
**EKUT_A**	3D T1-weighted magnetization prepared rapid gradient echo (MPRAGE)	3T Siemens Skyra	Sagittal	192	0mm	0,9 iso	900	2,32	2300	8	
**EKUT_B**	3D T1-weighted magnetization prepared rapid gradient echo (MPRAGE)	3T Siemens TrioTim	Sagittal	176-192	0mm	1 iso	1100	3,03	2300	8	
**EPICZ**	3D IR-prepared FSPGR T1-weighted sequence	3 T Discovery MR750 GE	Sagittal	184	0mm	1x1x1mm	650ms	3.7ms	9.2ms	12	Labate, Cherubini, et al. (2015) White matter abnormalities differentiate severe from benign temporal lobe epilepsy. *Epilepsia*. 2015 Jul;56(7):1109-16
**EPIGEN_3.0**	3D T1-weighted turbo field echo sequence	3 T Philips Achieva	Sagittal	160	0mm	1x1x1mm	-	3.9ms	8.5ms	8	Alhusaini, Whelan, et al. (2016). Temporal cortex morphology in mesial temporal lobe epilepsy patients and their asymptomatic siblings. *Cerebral Cortex*, 26(3): 1234-1241.
**EPIGEN_1.5**	3D T1-weighted spoiled gradient recalled sequence	1.5T GE Signa	Sagittal	124	0mm	-	450ms	4.2ms	10.1ms	20	Alhusaini, Scanlon, et al. (2013). Heritability of subcortical volumetric traits in mesial temporal lobe epilepsy. *PLoS One*, 8(4): e61880.
**Florence**	3D T1-weighted turbo field echo sequence	3 T Philips Achieva	Sagittal	191	1mm	1x1x1mm	-	3.7ms	8.1ms	8	
	3D T1-weighted turbo field echo sequence	1.5 T Philips	Sagittal	175	1mm	1x1x1mm	-	4.6ms	25ms	30	
**Greifswald**	3D T1-weighted magnetization prepared rapid gradient echo	3T Verio Siemens	Sagittal	174	0mm	0.9	900	2.58	1900	9	

	(MPRAGE)										
**IDIBAPS-HCB**	3D T1-weighted magnetization prepared rapid gradient echo (MPRAGE)	3T Trio, Siemens	Coronal	192	0mm	0.9	900	3.05	2000	9	
**KCL_CNS**	3D T1-weighted magnetization prepared rapid gradient echo (MPRAGE)	3T Signa HDx, GE	Coronal	196	0mm	1.1	450	2.84	7.18	20	
**KCL_CRF**	3D T1-weighted magnetization prepared rapid gradient echo (MPRAGE)	3T MR750, GE	Sagittal	196	0mm	1.05x1.05x1.2	400	3.016	7.312	11	
**Kuopio**	3D T1-weighted turbo field echo sequence	3 T Philips Achieva	Sagittal	190	0mm	1x1x1mm	-	3.7ms	8.2ms	8	
**MNI**	3D T1-weighted magnetization prepared rapid gradient echo (MPRAGE)	3T Trio, Siemens	Sagittal	176	0	1x1x1mm	900	2.98	2300	9	Hong SJ, Bernhardt BC, Schrader DS, Bernasconi N1, Bernasconi A. Whole-brain MRI phenotyping in dysplasia-related frontal lobe epilepsy; Neurology 2016; doi 10.1212/WNL.0000000000002374
**NYU**	3D T1-weighted magnetization prepared rapid gradient echo (MPRAGE)	3T Allegra, Siemens	Sagittal	128	1.3 mm	1x1x1.3mm	1.1 ms	3.25ms	2530 ms	7	Reyes A, et al. Resting stats fMRI distinguishes temporal lobe epilepsy subtypes. *Epilepsia*. 2016 doi: 10.1111/epi.13456.
**RMH**	3D T1-weighted magnetization prepared rapid gradient echo (MPRAGE)	3T Trio, Siemens	Coronal	192	0mm	0.9	900	2.21	1900	9	
**UCSD**	3D T1-weighted IR-FSPGR sequence	3 T Discovery MR750 GE	Sagittal	172	0mm	1x1x1mm	600ms	3.16ms	8.08	8	Kemmotsu, Kucukboyaci et al. (2014). Frontolimbic brain networks predict depressive symptoms in tempporal lobe epilepsy. *Epilepsy Research*, 108(9), 1554-1563.
**UNAM**	3D T1-weighted turbo field echo sequence	3 T Philips Achieva	Sagittal	186	0 mm	1x1x1mm		3.69 ms	8.06 ms	8	
**UNICAMP**	3D T1-weighted turbo field echo sequence	3 T Philips Achieva	Sagittal	180	0mm	1x1x1mm	-	3.2	7	8	de Campos BM, Coan AC, Lin Yasuda C, Casseb RF, Cendes F. Large-scale brain networks are distinctly affected in right and left mesial temporal lobe epilepsy *Hum Brain Mapp*. 2016 May 2.
**UNIMORE**	3D T1-weighted turbo field echo sequence	3 T Philips Achieva	Sagittal	170	0 mm	1x1x1mm	-	4.6 ms	9.9 ms	8	Vaudano, Ruggieri, Tondelli, et al (2014). The visual system in eyelid myoclonia with absences. *Ann Neurol*. 2014 Sep;76(3):412-27
**XMU**	3D IR-prepared FSPGR T1-weighted sequence	3 T GE	Axial	156	1.2mm	1.2x1.2x1.2mm	450ms	3.2ms	8.2ms	12	

Supplementary Table 4. Full list of cortical regions of interest, as extracted using FreeSurfer v5.3.0, with abbreviations.

Structure	Hemisphere	FreeSurfer abbreviation	Hemisphere	FreeSurfer abbreviation
**Banks of the superior temporal sulcus**	Left	L_bankssts	Right	R_bankssts
**Caudal anterior cingulate**	Left	L_caudalanteriorcingulate	Right	R_caudalanteriorcingulate
**Caudal middle frontal gyrus**	Left	L_caudalmiddlefrontal	Right	R_caudalmiddlefrontal
**Cuneus**	Left	L_cuneus	Right	R_cuneus
**Entorhinal gyrus**	Left	L_entorhinal	Right	R_entorhinal
**Fusiform gyrus**	Left	L_fusiform	Right	R_fusiform
**Inferior parietal gyrus**	Left	L_inferiorparietal	Right	R_inferiorparietal
**Inferior temporal gyrus**	Left	L_inferiortemporal	Right	R_inferiortemporal
**Isthmus cingulate**	Left	L_isthmuscingulate	Right	R_isthmuscingulate
**Lateral occipital gyrus**	Left	L_lateraloccipital	Right	R_lateraloccipital
**Lateral orbitofrontal gyrus**	Left	L_lateralorbitofrontal	Right	R_lateralorbitofrontal
**Lingual gyrus**	Left	L_lingual	Right	R_lingual
**Medial orbitofrontal gyrus**	Left	L_medialorbitofrontal	Right	R_medialorbitofrontal
**Middle temporal gyrus**	Left	L_middletemporal	Right	R_middletemporal
**Parahippocampal gyrus**	Left	L_parahippocampal	Right	R_parahippocampal
**Paracentral gyrus**	Left	L_paracentral	Right	R_paracentral
Pars opercularis	Left	L_parsopercularis	Right	R_parsopercularis
Pars orbitalis	Left	L_parsorbitalis	Right	R_parsorbitalis
Pars triangularis	Left	L_parstriangularis	Right	R_parstriangularis
**Pericalcarine gyrus**	Left	L_pericalcarine	Right	R_pericalcarine
**Postcentral gyrus**	Left	L_postcentral	Right	R_postcentral
**Posterior cingulate gyrus**	Left	L_posteriorcingulate	Right	R_posteriorcingulate
**Precentral gyrus**	Left	L_precentral	Right	R_precentral
**Precuneus**	Left	L_precuneus	Right	R_precuneus
**Rostral anterior cingulate**	Left	L_rostralanteriorcingulate	Right	R_rostralanteriorcingulate
**Rostral middle frontal gyrus**	Left	L_rostralmiddlefrontal	Right	R_rostralmiddlefrontal
**Superior frontal gyrus**	Left	L_superiorfrontal	Right	R_superiorfrontal
**Superior parietal gyrus**	Left	L_superiorparietal	Right	R_superiorparietal
**Superior temporal gyrus**	Left	L_superiortemporal	Right	R_superiortemporal
**Supramarginal gyrus**	Left	L_supramarginal	Right	R_supramarginal
**Frontal pole**	Left	L_frontalpole	Right	R_frontalpole
**Temporal pole**	Left	L_temporalpole	Right	R_temporalpole
**Transverse temporla gyrus**	Left	L_transversetemporal	Right	R_transversetemporal
**Insula**	Left	L_insula	Right	R_insula

Supplementary Table 5. Results of t-test for age differences, and chi-squared test for sex differences at each research centre.

	T-test for age differences				***Chi squared test for sex differences***	
Research centre	Difference of means	Standard error	T-statistic	*P*-value	**Pearson x(1) chi-square value**	***P*-value**
Bern	-2.0179	1.7209	1.1725	0.2434	**0.086**	**0.77**
Bonn	-0.4410	1.9861	-0.2220	0.8246	**0.969**	**0.325**
BRI	-1.4505	1.5569	-0.9316	0.3529	**3.844**	**0.049**
Brussels	7.1588	1.2681	5.6453	**1.10×10^{-7}**	**2.643**	**0.104**
CUBRIC	0.3750	1.6558	0.2265	0.8213	**0.000**	**1**
EKUT_A	-1.2419	2.2919	-0.5418	0.5892	**0.275**	**0.6**
EKUT_B	-4.2083	3.6302	-1.1593	0.2545	**1.418**	**0.234**
EPICZ	-0.6238	1.3679	-0.4560	0.6488	**3.342**	**0.068**
EPIGEN_3.0	1.4550	1.6612	0.8759	0.3828	**3.149**	**0.076**
EPIGEN_1.5	5.4940	2.0075	2.7367	**0.0074**	**4.576**	**0.032**
Florence	-7.2857	2.6622	-2.7368	**0.0117**	**1.327**	**0.204**
Greifswald	16.0241	2.5122	6.3786	**8.04×10^{-8}**	**0.527**	**0.468**
IDIBAPS-HCP	3.6393	1.2159	2.9930	**0.0032**	**0.091**	**0.763**
KCL_CNS	1.5147	1.2347	1.2268	0.2214	**0.052**	**0.82**
KCL_CRF	2.7359	3.3470	0.8174	0.4222	**1.139**	**0.286**
Kuopio	8.1876	0.7482	10.9429	**2.75×10^{-23}**	**1.035**	**0.309**
MNI	1.9223	1.3958	1.3772	0.1714	**1.214**	**0.271**
NYU	3.1310	1.2236	2.5588	**0.0111**	**0.972**	**0.324**
RMH	-0.7326	4.1484	-0.1766	0.8609	**0.508**	**0.476**
UCSD	0.7825	3.0656	0.2553	0.7993	**0.500**	**0.479**
UNAM	-1.7278	2.8626	-0.6036	0.5481	**0.188**	**0.664**
UNICAMP	5.5959	0.7979	7.0137	**5.98×10^{-12}**	**0.000**	**0.995**
UNIMORE	-0.1047	1.4477	-0.0723	0.9425	**0.022**	**0.881**
XMU	-2.7454	2.2762	-1.2061	0.2405	**0.515**	**0.473**

Supplementary Table 6. Effect sizes for volume differences (Cohen's *d*) between healthy controls and a subgroup of the 'all-epilepsies' phenotype, without evidence of hippocampal sclerosis or other lesions on MRI, controlling for age, sex, and intracranial volume.

Structure	Cohen's *d*	SE	Z score	95% CI	*P* value	FDR-adjusted *P* value	I^2	Number of controls	Number of cases
**Thalamus (RH)**	**-0.29**	**0.05**	**-5.88**	**-0.39 - -0.19**	**$3.98x10^{-9}$**	**6.69X10-8**	**6.28**	**1367**	**938**
Pallidum (RH)	-0.22	0.06	-3.42	-0.35 - -0.09	$6.33x10^{-4}$	2.13X10-3	40.52	1361	916
Pallidum (LH)	-0.19	0.10	-1.98	-0.38 - -0.002	$4.82x10^{-2}$	**8.43X10-2**	71.94	1260	843
Putamen (LH)	-0.17	0.09	-1.89	-0.34 - 0.006	$5.85x10^{-2}$	**9.83X10-2**	67.19	1287	895
Thalamus (LH)	-0.16	0.08	-2.09	-0.31 - -0.010	$3.64x10^{-2}$	**6.79X10-2**	57.99	1338	933
Putamen (RH)	-0.14	0.09	-1.58	-0.32 - 0.034	$1.14x10^{-1}$	1.65X10-1	69.77	1341	933
Caudate (LH)	-0.07	0.06	-1.11	-0.19- 0.0537	$2.68x10^{-1}$	3.31X10-1	38.24	1374	935
Hippocampus (RH)	-0.05	0.05	-1.00	-0.15 - 0.05	$3.18x10^{-1}$	3.87X10-1	14.84	1369	931
Hippocampus (LH)	-0.02	0.06	-0.32	-0.14 - 0.1	$7.49x10^{-1}$	8.17X10-1	34.01	1358	928
Accumbens (LH)	0.00	0.11	0.01	-0.2 - 0.2	$9.90x10^{-1}$	9.90X10-1	77.27	1358	936
Caudate (RH)	0.01	0.07	0.16	-0.12 - 0.14	$8.74x10^{-1}$	9.18X10-1	45.19	1375	937
Accumbens (RH)	0.06	0.12	0.48	-0.17 - 0.28	$6.34x10^{-1}$	7.50X10-1	81.55	1352	937
Lateral ventricle (LH)	0.19	0.05	4.02	0.01 - 0.29	$5.70x10^{-5}$	2.52X10-4	1.68	1373	936
**Lateral ventricle (RH)***	**0.22**	**0.05**	**4.33**	**0.12 - 0.32**	**$1.48x10^{-5}$**	**8.29X10-5**	**8.72**	**1374**	**936**
Amygdala (RH)*	0.24	0.06	3.75	0.11 - 0.37	$1.78x10^{-4}$	6.80X10-4	38.90	1343	929
**Amygdala (LH)***	**0.33**	**0.07**	**4.62**	**0.19 - 0.47**	**$3.85x10^{-6}$**	**2.94X10-5**	**51.24**	**1367**	**938**

Abbreviations: RH, right hemisphere, LH, left hemisphere, SE, standard error; CI, confidence interval; I^2, heterogeneity index. Uncorrected *p*-values are reported. Brain regions are ranked according to effect size (negative to positive). *P*-values were compared to an adjusted alpha level of $p<1.49x10^{-4}$. Significant brain regions are **highlighted in red with an asterisk (*)**.

Supplementary Table 7. Effect sizes for cortical thickness differences (Cohen's *d*) between healthy controls and a subgroup of the 'all-epilepsies' phenotype, without evidence of hippocampal sclerosis or other lesions on MRI, controlling for age, sex, and intracranial volume.

Structure	Cohen's *d*	SE	Z score	95% CI	*P* value	FDR-adjusted *P* value	I^2	Number of controls	Number of cases
**L_precentral**	**-0.37**	**0.05**	**-7.79**	-0.46 - -0.28	**6.47x10-15**	**5.43X10-13**	**0.00**	**1370**	**936**
**R_paracentral**	**-0.35**	**0.05**	**-7.41**	-0.44 - -0.26	**1.27x10-13**	**5.33X10-12**	**0.00**	**1379**	**937**
**R_precentral**	**-0.34**	**0.05**	**-7.21**	-0.43 - -0.25	**5.48x10-13**	**1.53X10-11**	**0.00**	**1374**	**935**
**L_caudalmiddlefrontal**	**-0.29**	**0.05**	**-6.13**	-0.38 - -0.2	**8.65x10-10**	**1.82X10-8**	**0.00**	**1375**	**939**
**L_paracentral**	**-0.26**	**0.05**	**-5.42**	-0.35 - -0.16	**5.85x10-8**	**8.19X10-7**	**0.00**	**1375**	**939**
**R_superiorfrontal**	**-0.24**	**0.06**	**-4.38**	-0.35 - -0.14	**1.19x10-5**	**7.38X10-5**	**23.10**	**1375**	**936**
**L_superiorfrontal**	**-0.24**	**0.06**	**-3.97**	-0.36 - -0.12	**7.34x10-5**	**3.08X10-4**	**34.93**	**1374**	**938**
**R_precuneus**	**-0.23**	**0.06**	**-4.08**	-0.34 - -0.12	**4.46x10-5**	**2.34X10-4**	**25.71**	**1379**	**933**
**R_caudalmiddlefrontal**	**-0.23**	**0.05**	**-4.88**	-0.32 - -0.14	**1.04x10-6**	**1.25X10-5**	**0.00**	**1378**	**937**
**R_parstriangularis**	**-0.23**	**0.05**	**-4.37**	-0.33 - -0.13	**1.23x10-5**	**7.38X10-5**	**13.47**	**1377**	**937**
**R_cuneus**	**-0.22**	**0.05**	**-4.73**	-0.31 - -0.13	**2.29x10-6**	**2.40X10-5**	**0.00**	**1377**	**935**
L_transversetemporal	-0.22	0.06	-3.59	-0.34 - -0.1	3.33x10-4	**1.22X10-3**	35.24	1372	939
**L_superiorparietal**	**-0.22**	**0.05**	**-4.69**	-0.31 - -0.13	**2.68x10-6**	**2.50X10-5**	**0.01**	**1374**	**936**
**R_lateraloccipital**	**-0.22**	**0.05**	**-4.66**	-0.31 - -0.13	**3.22x10-6**	**2.70X10-5**	**0.00**	**1378**	**936**
R_entorhinal	-0.22	0.06	-3.39	-0.34 - -0.09	6.90x10-4	**2.15X10-3**	28.82	1110	840
**R_superiorparietal**	**-0.22**	**0.05**	**-4.40**	-0.31 - -0.12	**1.09x10-5**	**7.38X10-5**	**5.32**	**1378**	**936**
R_lateralorbitofrontal	-0.21	0.07	-3.16	-0.34 - -0.08	1.55x10-3	**4.34X10-3**	43.69	1376	935
R_rostralmiddlefrontal	-0.20	0.06	-3.40	-0.32 - -0.09	6.70x10-4	**2.15X10-3**	32.06	1379	936
L_precuneus	-0.20	0.05	-3.57	-0.3 - -0.09	3.55x10-4	**1.24X10-3**	20.91	1374	937
**R_supramarginal**	**-0.20**	**0.05**	**-4.03**	**-0.29 - -0.1**	**5.59x10-5**	**2.52X10-4**	**0.00**	**1322**	**900**

**R_lingual**	**-0.19**	**0.05**	**-4.05**	**-0.28 - -0.1**	**5.09x10-5**	**2.52X10-4**	**0.00**	**1378**	**935**
**R_transversetemporal**	**-0.19**	**0.05**	**-3.95**	**-0.28 - -0.09**	**7.71x10-5**	**3.08X10-4**	**0.01**	**1379**	**937**
R_parsorbitalis	-0.18	0.06	-3.21	-0.29 - -0.07	1.34x10-3	**3.88X10-3**	25.94	1379	935
R_pericalcarine	-0.17	0.05	-3.27	-0.27 - -0.07	1.09x10-3	**3.27X10-3**	11.71	1379	936
L_lateraloccipital	-0.15	0.05	-3.06	-0.25 - -0.06	2.18x10-3	**5.91X10-3**	8.76	1375	939
L_pericalcarine	-0.15	0.06	-2.51	-0.27 - -0.03	1.22x10-2	**2.77X10-2**	31.78	1375	938
R_superiortemporal	-0.15	0.07	-2.25	-0.28 - -0.02	2.44x10-2	**4.79X10-2**	39.82	1282	898
L_cuneus	-0.15	0.05	-2.81	-0.25 - -0.04	4.88x10-3	**1.21X10-2**	15.45	1373	939
R_temporalpole	-0.15	0.05	-2.95	-0.24 - -0.05	3.19x10-3	**8.32X10-3**	6.72	1375	934
L_entorhinal	-0.14	0.09	-1.60	-0.32 - 0.03	1.09x10-1	1.61X10-1	63.11	1132	852
L_parstriangularis	-0.14	0.05	-2.94	-0.23 - -0.05	3.27x10-3	**8.32X10-3**	0.01	1375	939
L_fusiform	-0.13	0.06	-2.26	-0.24 - -0.02	2.41x10-2	**4.79X10-2**	27.43	1372	938
R_fusiform	-0.13	0.07	-1.88	-0.26 - 0.01	5.98x10-2	9.85X10-2	45.10	1379	936
L_supramarginal	-0.12	0.06	-2.08	-0.24 - -0.01	3.78x10-2	6.90X10-2	27.53	1331	900
L_parsorbitalis	-0.12	0.05	-2.60	-0.21 - -0.03	9.39x10-3	**2.25X10-2**	0.00	1375	938
L_lingual	-0.12	0.05	-2.56	-0.21 - -0.03	1.05x10-2	**2.45X10-2**	0.00	1374	939
L_temporalpole	-0.12	0.07	-1.65	-0.26 - 0.02	9.86x10-2	1.51X10-1	52.42	1369	936
R_middletemporal	-0.12	0.05	-2.46	-0.21 - -0.02	1.37x10-2	**3.03X10-2**	0.01	1368	932
L_rostralmiddlefrontal	-0.12	0.07	-1.76	-0.24 - 0.01	7.82x10-2	1.22X10-1	42.84	1374	938
R_parsopercularis	-0.12	0.05	-2.29	-0.21 - -0.02	2.20x10-2	4.74X10-2	9.54	1377	937
L_parsopercularis	-0.11	0.05	-2.25	-0.21 - -0.01	2.45x10-2	4.79X10-2	10.79	1373	938
R_medialorbitofrontal	-0.11	0.06	-1.84	-0.22 - 0.01	6.62x10-2	1.07X10-1	28.75	1361	912
R_frontalpole	-0.11	0.05	-2.26	-0.2 - -0.01	2.39x10-2	**4.79X10-2**	0.00	1379	936
L_posteriorcingulate	-0.11	0.07	-1.57	-0.24 - 0.03	1.17x10-1	1.65X10-1	45.74	1375	938
L_superiortemporal	-0.10	0.06	-1.63	-0.23 - 0.02	1.04x10-1	1.56X10-1	36.71	1262	894
R_inferiorparietal	-0.10	0.05	-2.04	-0.2 - 0	4.11x10-2	7.35X10-2	10.41	1368	928
R_parahippocampal	-0.10	0.05	-2.10	-0.19 - -0.01	3.61x10-2	6.79X10-2	0.00	1375	935
L_frontalpole	-0.10	0.05	-1.97	-0.2 - 0	4.92x10-2	8.43X10-2	8.99	1373	938
L_isthmuscingulate	-0.09	0.06	-1.52	-0.22 - 0.03	1.28x10-1	1.73X10-1	36.55	1374	937

R_bankssts	-0.08	0.06	-1.41	-0.2 - 0.03	1.57x10-1	2.03X10-1	31.10	1349	922
L_inferiorparietal	-0.08	0.05	-1.80	-0.18 - 0.01	7.24x10-2	1.15X10-1	0.00	1364	934
L_lateralorbitofrontal	-0.08	0.07	-1.15	-0.22 - 0.06	2.48x10-1	3.11X10-1	48.77	1370	938
R_inferiortemporal	-0.08	0.05	-1.56	-0.18 - 0.02	1.18x10-1	1.65X10-1	9.50	1377	935
R_insula	-0.07	0.05	-1.54	-0.17 - 0.02	1.24x10-1	1.71X10-1	0.01	1340	929
L_postcentral	-0.07	0.05	-1.50	-0.16 - 0.02	1.32x10-1	1.76X10-1	1.57	1372	936
L_middletemporal	-0.07	0.05	-1.47	-0.17 - 0.02	1.42x10-1	1.86X10-1	0.01	1276	901
R_posteriorcingulate	-0.06	0.07	-0.96	-0.2 - 0.07	3.36x10-1	4.03X10-1	44.56	1379	937
L_parahippocampal	-0.06	0.05	-1.27	-0.15 - 0.03	2.05x10-1	2.61X10-1	0.00	1362	934
L_rostralanteriorcingulate	-0.03	0.07	-0.40	-0.18 - 0.12	6.88x10-1	7.92X10-1	54.98	1370	933
L_inferiortemporal	-0.02	0.06	-0.32	-0.14 - 0.1	7.46x10-1	8.17X10-1	32.90	1374	934
L_medialorbitofrontal	-0.02	0.05	-0.37	-0.11 - 0.08	7.14x10-1	8.04X10-1	2.06	1357	914
R_postcentral	-0.02	0.05	-0.36	-0.11 - 0.08	7.18x10-1	8.04X10-1	0.02	1373	935
R_caudalanteriorcingulate	-0.01	0.05	-0.27	-0.12 - 0.09	7.87x10-1	8.48X10-1	16.26	1377	936
R_isthmuscingulate	-0.01	0.06	-0.16	-0.12 - 0.1	8.71x10-1	9.18X10-1	27.31	1376	936
L_insula	0.00	0.05	-0.06	-0.11 - 0.1	9.55x10-1	9.67X10-1	18.38	1335	924
L_bankssts	0.00	0.07	0.07	-0.13 - 0.14	9.46x10-1	9.67X10-1	40.59	1314	882
R_rostralanteriorcingulate	0.01	0.07	0.09	-0.14 - 0.15	9.30x10-1	9.64X10-1	53.44	1377	935
L_caudalanteriorcingulate	0.02	0.05	0.46	-0.08 - 0.13	6.47x10-1	7.55X10-1	13.57	1370	926

Abbreviations: RH, right hemisphere, LH, left hemisphere, SE, standard error; CI, confidence interval; I^2, heterogeneity index. Abbreviations for all cortical brain regions are provided in Supplementary Table 4. Uncorrected *p*-values are reported. Brain regions are ranked according to effect size (negative to positive). *P*-values were compared to a Bonferroni-adjusted alpha level of $p<1.49x10^{-4}$. Brain regions exhibiting statistically significant case-control differences are **highlighted in red with an asterisk (*)**. FDR-significant p-values are **highlighted in bold**.

Supplementary Table 8. Beta coefficients, standard errors and *P*-values for the meta-regression of duration of epilepsy against subcortical volume and cortical thickness, controlling for sex and intracranial volume.

Structure	Beta	SE	Z value	P value	CI (lower)	CI (upper)	I^2
All epilepsies							
Right putamen	-13.014	1.178	-11.044	$2.34x10^{-28}$	-15.323	-10.704	0.000
Left caudate	-7.062	0.853	-8.275	$1.29x10^{-16}$	-8.734	-5.389	0.000
Right caudate	-7.168	0.885	-8.104	$5.33x10^{-16}$	-8.902	-5.435	0.000
Left thalamus*	-13.580	1.725	-7.871	$3.52x10^{-15}$	-16.961	-10.198	9.655
Left pallidum	-3.216	0.428	-7.512	$5.83x10^{-14}$	-4.055	-2.377	0.000
Right thalamus*	-12.252	1.660	-7.381	$1.58x10^{-13}$	-15.506	-8.998	27.547
Left putamen	-11.414	1.586	-7.196	$6.21x10^{-13}$	-14.523	-8.305	23.077
Left hippocampus*	-8.320	1.162	-7.159	$8.16x10^{-13}$	-10.598	-6.042	1.755
Left accumbens	-1.471	0.239	-6.150	$7.73x10^{-10}$	-1.939	-1.002	0.000
Right accumbens	-1.277	0.209	-6.094	$1.10x10^{-9}$	-1.687	-0.866	0.000
Right pallidum*	-2.668	0.511	-5.221	$1.78x10^{-7}$	-3.669	-1.666	39.771
Left Lateral Ventricle	60.563	12.035	5.032	$4.85x10^{-7}$	36.974	84.151	46.456
Right Lateral Ventricle	58.496	12.034	4.861	$1.17x10^{-6}$	34.910	82.082	55.051
R_precentral*	-0.003	0.000	-10.176	$2.54x10^{-24}$	-0.004	-0.002	0.000
R_precuneus*	-0.003	0.000	-9.389	$6.03x10^{-21}$	-0.003	-0.002	1.755
R_supramarginal*	-0.003	0.000	-8.985	$2.58x10^{-19}$	-0.004	-0.002	4.848
R_caudalmiddlefrontal*	-0.003	0.000	-8.516	$1.65x10^{-17}$	-0.003	-0.002	2.340
L_precuneus	-0.002	0.000	-8.320	$8.80x10^{-17}$	-0.003	-0.002	0.601
L_parsopercularis	-0.003	0.000	-8.252	$1.56x10^{-16}$	-0.004	-0.002	11.329
L_caudalmiddlefrontal*	-0.003	0.000	-7.968	$1.61x10^{-15}$	-0.003	-0.002	0.000
R_lingual	-0.002	0.000	-7.760	$8.51x10^{-15}$	-0.003	-0.002	5.800
R_inferiorparietal	-0.002	0.000	-7.509	$5.96x10^{-14}$	-0.003	-0.002	6.007
L_supramarginal*	-0.003	0.000	-7.365	$1.77x10^{-13}$	-0.003	-0.002	14.422
L_superiorparietal	-0.002	0.000	-7.210	$5.58x10^{-13}$	-0.002	-0.001	0.000
R_parsopercularis*	-0.003	0.000	-7.210	$5.59x10^{-13}$	-0.003	-0.002	12.838
L_superiortemporal	-0.003	0.000	-7.135	$9.71x10^{-13}$	-0.004	-0.002	16.273
R_superiorparietal	-0.002	0.000	-7.055	$1.72x10^{-12}$	-0.002	-0.001	2.916
L_superiorfrontal*	-0.004	0.001	-7.039	$1.94x10^{-12}$	-0.005	-0.003	47.219
L_pericalcarine	-0.002	0.000	-6.919	$4.54x10^{-12}$	-0.003	-0.001	5.164
L_precentral*	-0.003	0.000	-6.884	$5.84x10^{-12}$	-0.004	-0.002	40.608

L_rostralmiddlefrontal	-0.002	0.000	-6.705	$2.02x10^{-11}$	-0.003	-0.002	15.822
L_cuneus	-0.002	0.000	-6.674	$2.49x10^{-11}$	-0.003	-0.001	0.139
L_parstriangularis*	-0.003	0.000	-6.647	$2.99x10^{-11}$	-0.004	-0.002	22.266
R_superiorfrontal*	-0.003	0.000	-6.582	$4.65x10^{-11}$	-0.004	-0.002	37.613
R_superiortemporal	-0.003	0.000	-6.528	$6.67x10^{-11}$	-0.004	-0.002	15.295
L_transversetemporal*	-0.004	0.001	-6.460	$1.05x10^{-10}$	-0.005	-0.002	18.067
L_lingual	-0.002	0.000	-6.457	$1.07x10^{-10}$	-0.003	-0.001	14.475
R_middletemporal	-0.003	0.000	-6.382	$1.75x10^{-10}$	-0.004	-0.002	23.260
R_bankssts	-0.003	0.000	-6.246	$4.21x10^{-10}$	-0.004	-0.002	15.316
L_bankssts	-0.002	0.000	-6.244	$4.27x10^{-10}$	-0.003	-0.002	4.710
L_inferiorparietal	-0.002	0.000	-6.166	$7.00x10^{-10}$	-0.003	-0.001	3.520
R_transversetemporal*	-0.003	0.000	-6.140	$8.24x10^{-10}$	-0.004	-0.002	0.000
R_postcentral	-0.002	0.000	-6.122	$9.24x10^{-10}$	-0.002	-0.001	3.391
R_pericalcarine	-0.002	0.000	-6.095	$1.09x10^{-9}$	-0.002	-0.001	9.756
R_cuneus*	-0.002	0.000	-6.094	$1.10x10^{-9}$	-0.002	-0.001	3.769
R_parstriangularis*	-0.002	0.000	-5.874	$4.24x10^{-9}$	-0.003	-0.002	19.719
L_posteriorcingulate	-0.002	0.000	-5.841	$5.18x10^{-9}$	-0.003	-0.001	3.374
L_postcentral	-0.002	0.000	-5.572	$2.52x10^{-8}$	-0.002	-0.001	17.783
L_parsorbitalis	-0.003	0.001	-5.514	$3.50x10^{-8}$	-0.004	-0.002	18.571
L_middletemporal	-0.002	0.000	-5.447	$5.13x10^{-8}$	-0.003	-0.001	18.361
R_lateraloccipital	-0.002	0.000	-5.433	$5.54x10^{-8}$	-0.002	-0.001	0.000
R_posteriorcingulate	-0.002	0.000	-5.266	$1.40x10^{-7}$	-0.002	-0.001	0.000
R_insula	-0.002	0.000	-4.840	$1.30x10^{-6}$	-0.003	-0.001	24.620
L_lateraloccipital	-0.001	0.000	-4.810	$1.51x10^{-6}$	-0.002	-0.001	5.257
R_isthmuscingulate	-0.002	0.000	-4.741	$2.12x10^{-6}$	-0.003	-0.001	0.000
R_fusiform	-0.002	0.000	-4.668	$3.04x10^{-6}$	-0.003	-0.001	31.915
R_rostralmiddlefrontal	-0.002	0.000	-4.612	$4.00x10^{-6}$	-0.003	-0.001	49.726
L_fusiform	-0.002	0.000	-4.595	$4.34x10^{-6}$	-0.003	-0.001	20.788
L_paracentral*	-0.002	0.001	-4.556	$5.22x10^{-6}$	-0.004	-0.001	55.813
R_lateralorbitofrontal	-0.002	0.001	-4.551	$5.34x10^{-6}$	-0.004	-0.001	50.450
R_paracentral*	-0.002	0.001	-4.540	$5.63x10^{-6}$	-0.003	-0.001	53.655
L_isthmuscingulate	-0.002	0.000	-4.315	$1.60x10^{-5}$	-0.003	-0.001	16.341
L_insula	-0.002	0.000	-4.238	$2.26x10^{-5}$	-0.003	-0.001	22.709
R_inferiortemporal	-0.002	0.000	-3.966	$7.30x10^{-5}$	-0.002	-0.001	16.206
MTLE-R							
Right hippocampus	-22.42	4.223	-5.310	$1.1x10^{-7}$	-30.700	-14.146	43.664
Right putamen	-13.02	2.990	-4.355	$1.33x10^{-5}$	-18.882	-7.160	0.000

Right thalamus	-18.11	4.228	-4.284	$1.84x10^{-5}$	-26.398	-9.825	28.650
L_transversetemporal	-0.007	0.002	-3.93	$8.39x10^{-5}$	-0.010	-0.003	39.64
R_caudalmiddlefrontal	-0.003	0.001	-3.80	$1.45x10^{-4}$	-0.005	-0.002	1.088
IGE							
Right putamen	-15.452	3.200	-4.829	$1.37x10^{-6}$	-21.724	-9.180	0.000
Left pallidum	-4.768	1.058	-4.508	$6.54x10^{-6}$	-6.841	-2.695	0.000
Left putamen	-15.386	3.638	-4.229	$2.35x10^{-5}$	-22.516	-8.255	0.000
Right caudate	-11.690	2.787	-4.194	$2.74x10^{-5}$	-17.153	-6.227	29.313
Right lateral ventricle	76.791	18.328	4.190	$2.79x10^{-5}$	40.868	112.714	0.000
Left *pars opercularis*	-0.005	0.001	-3.842	$1.22x10^{-4}$	-0.008	-0.003	21.714
All other epilepsies							
Left lateral ventricle*	59.6	13.599	4.38	**1.17x10-5**	32.94	86.26	34.86
R_precentral*	-0.0036	0.0005	-7.110	**1.2X10-12**	-0.00459	-0.00261	0.00
L_superiortemporal	-0.0042	0.0006	-6.588	**4.5X10-11**	-0.00542	-0.00293	0.00
L_supramarginal	-0.0034	0.0006	-6.158	**7.4X10-10**	-0.00449	-0.00232	0.43
L_superiorfrontal*	-0.0034	0.0006	-6.060	**1.4x10-9**	-0.00450	-0.00230	0.00
L_bankssts	-0.0041	0.0007	-5.976	**2.3x10-9**	-0.00539	-0.00273	7.47
R_precuneus*	-0.0029	0.0005	-5.847	**5.0x10-9**	-0.00393	-0.00196	0.00
L_cuneus	-0.0027	0.0005	-5.528	**3.2x10-8**	-0.00369	-0.00176	0.00
R_pericalcarine	-0.0024	0.0004	-5.487	**4.1x10-8**	-0.00328	-0.00155	0.00
R_caudalmiddlefrontal*	-0.0030	0.0006	-5.394	**6.9x10-8**	-0.00413	-0.00193	0.00
R_superiortemporal	-0.0039	0.0007	-5.370	**7.9x10-8**	-0.00529	-0.00246	13.04
R_cuneus*	-0.0025	0.0005	-5.355	**8.6x10-8**	-0.00348	-0.00161	0.00
R_supramarginal*	-0.0032	0.0006	-5.178	**2.2x10-7**	-0.00440	-0.00198	13.50
R_superiorfrontal*	-0.0028	0.0006	-5.113	**3.2x10-7**	-0.00391	-0.00174	0.00
L_caudalmiddlefrontal*	-0.0028	0.0005	-5.101	**3.4x10-7**	-0.00385	-0.00171	0.00
R_parstriangularis*	-0.0032	0.0006	-5.020	**5.2x10-7**	-0.00446	-0.00195	7.00
R_parsorbitalis	-0.0040	0.0008	-4.781	**1.7x10-6**	-0.00565	-0.00236	6.64
L_lingual	-0.0023	0.0005	-4.712	**2.5x10-6**	-0.00326	-0.00134	3.09
L_parsopercularis	-0.0028	0.0006	-4.617	**3.9x10-6**	-0.00396	-0.00160	6.50
R_parsopercularis	-0.0029	0.0006	-4.539	**5.7x10-6**	-0.00411	-0.00163	5.75
L_pericalcarine	-0.0024	0.0005	-4.535	**5.8x10-6**	-0.00346	-0.00137	23.05
L_rostralmiddlefrontal	-0.0023	0.0005	-4.514	**6.4x10-6**	-0.00333	-0.00131	6.62
R_inferiorparietal	-0.0023	0.0005	-4.476	**7.6x10-6**	-0.00335	-0.00131	6.40
R_middletemporal	-0.0033	0.0008	-4.360	**1.3x10-5**	-0.00480	-0.00182	25.97

L_parsorbitalis	-0.0041	0.0009	-4.326	**1.5x10-5**	-0.00591	-0.00222	20.36
R_insula	-0.0027	0.0006	-4.244	**2.2x10-5**	-0.00390	-0.00144	2.94
L_rostralanteriorcingulate	-0.0034	0.0008	-4.173	**3.0x10-5**	-0.00494	-0.00178	5.64
L_inferiorparietal	-0.0026	0.0006	-4.162	**3.1x10-5**	-0.00378	-0.00136	22.34
L_precuneus*	-0.0027	0.0006	-4.161	**3.2x10-5**	-0.00392	-0.00141	25.74
R_isthmuscingulate	-0.0031	0.0008	-4.008	**6.1x10-5**	-0.00454	-0.00156	4.58
R_medialorbitofrontal	-0.0024	0.0006	-3.994	**6.5x10-5**	-0.00356	-0.00122	4.64
R_postcentral	-0.0021	0.0005	-3.946	**8.0x10-5**	-0.00314	-0.00106	18.44
L_parstriangularis	-0.0026	0.0007	-3.936	**8.3x10-5**	-0.00386	-0.00129	3.31
R_bankssts	-0.0028	0.0007	-3.852	**1.2x10-4**	-0.00421	-0.00137	9.47
R_lateralorbitofrontal	-0.0029	0.0007	-3.822	**1.3x10-4**	-0.00432	-0.00139	30.56

Abbreviations: Beta, correlation beta; CI, confidence interval; I^2, heterogeneity index; MTLE-R, temporal lobe epilepsy with right mesial temporal sclerosis; IGE, idiopathic generalised epilepsy. Uncorrected p-values are reported. Correlations for subcortical regions that failed to survive Bonferroni correction ($p<1.49x10^{-4}$ for subcortical regions; $p<1.49x10^{-4}$ for cortical regions) are not reported (see Methods for statistical threshold determination). See Supplementary Table 3 for a list of cortical abbreviations. Regions highlighted in yellow and marked with an asterisk (*) also showed evidence of subcortical volume loss / cortical thinning in the main case-control analysis.

Supplementary Table 9. Beta coefficients, standard errors and *P*-values for the meta-regression of age at onset of epilepsy against subcortical volume and cortical thickness, controlling for sex and intracranial volume.

Structure	Beta	SE	Z value	P value	CI (lower)	CI (upper)	I^2
All epilepsies							
Right accumbens	-1.84	0.27	-6.68	$2.32x10^{-11}$	-2.38	-1.30	23.36
Right putamen	-9.13	1.61	-5.66	$1.48x10^{-8}$	-12.29	-5.97	19.37
Left caudate	-5.12	0.94	-5.47	$4.56x10^{-8}$	-6.96	-3.29	0.00
Left accumbens	-1.76	0.34	-5.24	$1.65x10^{-7}$	-2.42	-1.10	29.05
Right caudate	-4.91	0.99	-4.96	$7.08x10^{-7}$	-6.85	-2.97	0.00
Left putamen	-8.80	1.90	-4.64	$3.44x10^{-6}$	-12.51	-5.08	23.74
L_rostralanteriorcingulate	-0.0044	0.0005	-9.21	$3.17x10^{-20}$	-0.01	0.00	0.00
L_superiorfrontal*	-0.0030	0.0004	-7.91	$2.66x10^{-15}$	0.00	0.00	7.30
R_medialorbitofrontal	-0.0029	0.0004	-7.81	$5.86x10^{-15}$	0.00	0.00	0.00
R_parsopercularis*	-0.0032	0.0004	-7.52	$5.40x10^{-14}$	0.00	0.00	20.48
L_parsopercularis	-0.0027	0.0004	-7.42	$1.20x10^{-13}$	0.00	0.00	7.39
R_lateralorbitofrontal	-0.0027	0.0004	-6.44	$1.16x10^{-10}$	0.00	0.00	14.65
L_frontalpole	-0.0039	0.0006	-6.28	$3.35x10^{-10}$	-0.01	0.00	0.00
R_rostralanteriorcingulate	-0.0038	0.0006	-6.15	$7.67x10^{-10}$	-0.01	0.00	21.68
R_superiorfrontal*	-0.0027	0.0004	-6.11	$9.77x10^{-10}$	0.00	0.00	28.65
L_medialorbitofrontal	-0.0030	0.0005	-6.10	$1.06x10^{-9}$	0.00	0.00	34.08
R_frontalpole	-0.0038	0.0006	-6.02	$1.71x10^{-9}$	-0.01	0.00	0.00
L_parstriangularis*	-0.0028	0.0005	-5.94	$2.78x10^{-9}$	0.00	0.00	26.73
R_rostralmiddlefrontal	-0.0021	0.0003	-5.90	$3.56x10^{-9}$	0.00	0.00	19.15
L_transversetemporal*	-0.0028	0.0005	-5.73	$1.03x10^{-8}$	0.00	0.00	0.00
R_parsorbitalis	-0.0028	0.0005	-5.64	$1.71x10^{-8}$	0.00	0.00	0.00
L_insula	-0.0030	0.0006	-5.37	$8.09x10^{-8}$	0.00	0.00	46.00
R_middletemporal	-0.0021	0.0004	-5.20	$1.98x10^{-7}$	0.00	0.00	9.96
R_lingual	-0.0015	0.0003	-5.15	$2.65x10^{-7}$	0.00	0.00	0.00

L_caudalanteriorcingulate	-0.0029	0.0006	-5.08	3.78×10^{-7}	0.00	0.00	0.00
R_parstriangularis*	-0.0030	0.0006	-4.98	6.51×10^{-7}	0.00	0.00	54.97
L_lateralorbitofrontal	-0.0027	0.0005	-4.94	7.69×10^{-7}	0.00	0.00	42.80
L_isthmuscingulate	-0.0031	0.0006	-4.88	1.08×10^{-6}	0.00	0.00	33.06
L_lingual	-0.0016	0.0003	-4.83	1.34×10^{-6}	0.00	0.00	9.12
L_parsorbitalis	-0.0030	0.0006	-4.77	1.81×10^{-6}	0.00	0.00	26.43
R_inferiortemporal	-0.0021	0.0005	-4.71	2.48×10^{-6}	0.00	0.00	22.02
R_cuneus*	-0.0014	0.0003	-4.57	4.90×10^{-6}	0.00	0.00	0.00
R_inferiorparietal	-0.0019	0.0004	-4.56	5.19×10^{-6}	0.00	0.00	32.22
L_postcentral	-0.0015	0.0004	-4.16	3.13×10^{-5}	0.00	0.00	24.06
L_middletemporal	-0.0023	0.0006	-4.08	4.50×10^{-5}	0.00	0.00	44.78
R_fusiform	-0.0018	0.0004	-4.02	5.75×10^{-5}	0.00	0.00	30.22
L_cuneus	-0.0012	0.0003	-3.88	1.03×10^{-4}	0.00	0.00	0.00
L_inferiorparietal	-0.0023	0.0006	-3.86	1.15×10^{-4}	0.00	0.00	61.76
MTLE-L							
L_insula	-0.0044	0.0010	-4.19	2.77×10^{-5}	-0.01	0.00	15.94
L_pericalcarine	-0.0034	0.0009	-3.81	1.39×10^{-4}	-0.01	0.00	20.67
MTLE-R							
L_medialorbitofrontal	-0.0044	0.0009	-5.16	4.47×10^{-7}	-0.01	0.00	0.00
IGE							
Right amygdala	-6.12	1.61	-3.81	1.37×10^{-4}	-9.27	-2.98	0.00
L_rostralanteriorcingulate	-0.01	0.00	-3.87	1.09×10^{-4}	-0.01	0.00	3.37
All other epilepsies							
Right accumbens	-2.212	0.414	-5.341	9.24x10-8	-3.023	-1.400	21.163
Left accumbens	-2.171	0.407	-5.339	9.37x10-8	-2.968	-1.374	4.901
Right lateral ventricle*	57.730	11.021	5.238	1.62x10-7	36.129	79.331	0.000
Right putamen	-9.899	2.190	-4.520	6.18x10-6	-14.191	-5.607	16.795
L_medialorbitofrontal	-0.0037	0.00050	-7.478	7.54X10-14	-0.0047	-0.0028	0.00
L_parsopercularis	-0.0032	0.00049	-6.536	6.31X10-11	-0.0042	-0.0023	0.00
R_posteriorcingulate	-0.0033	0.00050	-6.505	7.76X10-11	-0.0042	-0.0023	0.00

L_superiorfrontal*	-0.0028	0.00052	-5.529	3.21x10-8	-0.0039	-0.0018	3.11
L_insula	-0.0029	0.00054	-5.404	6.51x10-8	-0.0039	-0.0018	6.54
L_isthmuscingulate	-0.0035	0.00065	-5.363	8.17x10-8	-0.0048	-0.0022	0.68
L_posteriorcingulate	-0.0033	0.00061	-5.361	8.28x10-8	-0.0045	-0.0021	16.11
R_isthmuscingulate	-0.0034	0.00064	-5.264	1.41x10-7	-0.0047	-0.0021	0.00
L_middletemporal	-0.0029	0.00058	-4.971	6.65x10-7	-0.0040	-0.0017	4.86
R_rostralanteriorcingulate	-0.0032	0.00067	-4.794	1.63x10-6	-0.0045	-0.0019	0.00
L_lateralorbitofrontal	-0.0030	0.00062	-4.781	1.75x10-6	-0.0042	-0.0018	20.48
R_supramarginal*	-0.0023	0.00048	-4.718	2.38x10-6	-0.0032	-0.0013	0.00
L_precuneus*	-0.0021	0.00046	-4.454	8.42x10-6	-0.0030	-0.0012	0.00
L_supramarginal	-0.0022	0.00049	-4.452	8.51x10-6	-0.0032	-0.0012	0.00
R_inferiorparietal	-0.0019	0.00044	-4.307	1.65x10-5	-0.0027	-0.0010	0.00
R_parstriangularis*	-0.0028	0.00065	-4.212	2.53x10-5	-0.0040	-0.0015	19.51
L_inferiorparietal	-0.0019	0.00046	-4.188	2.82x10-5	-0.0028	-0.0010	0.00
R_bankssts	-0.0028	0.00068	-4.136	3.53x10-5	-0.0041	-0.0015	9.70
L_frontalpole	-0.0037	0.00092	-4.054	5.03x10-5	-0.0056	-0.0019	1.19
L_parstriangularis	-0.0029	0.00073	-4.013	6.00x10-5	-0.0044	-0.0015	24.61
L_rostralmiddlefrontal	-0.0025	0.00062	-3.976	7.02x10-5	-0.0037	-0.0013	38.06
R_precuneus*	-0.0018	0.00047	-3.969	7.23x10-5	-0.0028	-0.0009	0.00
L_rostralanteriorcingulate	-0.0033	0.00084	-3.956	7.62x10-5	-0.0049	-0.0017	17.94
R_superiorfrontal*	-0.0025	0.00065	-3.850	1.18x10-4	-0.0038	-0.0012	28.07

Abbreviations: Beta, correlation beta; CI, confidence interval; I^2, heterogeneity index; MTLE-R, temporal lobe epilepsy with right mesial temporal sclerosis; IGE, idiopathic generalised epilepsy. See Supplementary Table 3 for a list of cortical abbreviations. Uncorrected p-values are reported. Correlations for subcortical volumes that failed to survive Bonferroni correction (p<1.49x10^{-4}) or cortical thicknesses that failed to survive Bonferroni correction (p<1.49x10^{-4}) are not reported (see Methods for statistical threshold determination). Regions highlighted in yellow and marked with an asterisk (*) also showed evidence of subcortical volume loss / cortical thinning in the main case-control analysis.

Supplementary Table 10. Effect sizes for subcortical volume differences (Cohen's *d*) between healthy controls and the 'all-epilepsies' phenotype; 3T field strength only.

Structure	Cohen's *d* (3T only)	Cohen's *d* (full sample)	SE	Z score	95% CI	*P* value (3T only)	*P* value (full sample)	I^2	Number of controls	Number of cases
**Thalamus (RH)**	**-0.36**	**-0.37**	**0.05**	**0.05**	**-0.46 - -0.26**	**3.85x10-12**	**7.67x10-14**	**46.86**	**1650**	**2070**
**Thalamus (LH)**	**-0.34**	**-0.36**	**0.07**	**0.07**	**-0.49 - -0.2**	**3.05x10-6**	**1.31x10-6**	**74.13**	**1621**	**2037**
**Hippocampus (LH)**	**-0.34**	**-0.35**	**0.07**	**0.07**	**-0.48 - -0.2**	**1.01x10-6**	**3.04x10-7**	**70.84**	**1641**	**2058**
**Hippocampus (RH)**	**-0.32**	**-0.34**	**0.05**	**0.05**	**-0.42 - -0.22**	**7.37x10-10**	**6.63x10-10**	**48.33**	**1653**	**2062**
**Pallidum (RH)**	**-0.31**	**-0.32**	**0.06**	**0.06**	**-0.42 - -0.2**	**5.60x10-8**	**8.32x10-9**	**57.36**	**1644**	**2045**
**Putamen (LH)**	**-0.25**	**-0.25**	**0.07**	**0.07**	**-0.37 - -0.12**	**1.71x10-4**	**8.30x10-5**	**66.34**	**1570**	**2008**
Putamen (RH)	-0.23	-0.24	0.08	0.08	-0.39 - -0.08	3.03x10-3	1.53x10-3	77.86	1624	2060
Pallidum (LH)	-0.18	-0.17	0.08	0.08	-0.35 - -0.02	3.13x10-2	3.53x10-2	80.21	1543	1924
Caudate (LH)	-0.12	-0.12	0.05	0.05	-0.22 - -0.02	2.17x10-2	1.56x10-2	49.84	1658	2068
Accumbens (RH)	-0.08	-0.06	0.10	0.10	-0.26 - 0.11	4.35x10-1	4.94x10-1	85.36	1636	2070
Caudate (RH)	-0.07	-0.08	0.06	0.06	-0.18 - 0.04	2.29x10-1	1.36x10-1	56.84	1659	2070
Accumbens (LH)	-0.02	-0.02	0.08	0.08	-0.17 - 0.13	7.71x10-1	8.33x10-1	76.81	1641	2069
Amygdala (LH)	0.10	0.10	0.07	0.07	-0.04 - 0.25	1.73x10-1	1.47x10-1	74.91	1651	2057
Amygdala (RH)	0.12	0.11	0.06	0.06	-0.01 - 0.24	6.94x10-2	6.76x10-2	65.27	1627	2041
**Right lateral ventricle**	**0.26**	0.27	**0.03**	**0.03**	**0.19 - 0.33**	**9.25x10-14**	**3.73x10-15**	**0.00**	**1656**	**2071**
**Left lateral ventricle**	**0.28**	0.29	**0.04**	**6.62**	**0.2 - 0.36**	**3.61x10-11**	**2.14x10-12**	**24.31**	**1656**	**2069**

Abbreviations: Beta, correlation beta; CI, confidence interval; I^2, heterogeneity index. All *p*-values are reported, uncorrected. For ease of comparison, Cohen's *d* estimates and *p*-values extracted using 3T data only are provided alongside Cohen's d estimates and *p*-values extracted from the full sample, combining 1.5T and 3T datasets. *P*-values that passed the Bonferroni-adjusted alpha level (p<1.49x10^{-4}) are highlighted in bold red (see Methods for statistical threshold determination). Brain regions reported as significant in the full sample, but not in the 3T sample, are highlighted in yellow.

Supplementary Table 11. Effect sizes for subcortical volume differences (Cohen's *d*) between healthy controls and the 'MTLE-L' phenotype; 3T field strength only.

Structure	Cohen's *d* (3T only)	Cohen's *d* (full sample)	SE	Z score	95% CI	*P* value (3T only)	*P* value (full sample)	I^2	Number of controls	Number of cases
**Hippocampus (LH)**	**-1.75**	**-1.73**	**0.21**	**-8.49**	**-2.15 - -1.34**	**2.10x10-17**	**1.35x10-19**	**86.41**	**1346**	**382**
**Thalamus (LH)**	**-0.82**	**-0.84**	**0.13**	**-6.14**	**-1.09 - -0.56**	**8.07x10-10**	**2.19x10-11**	**71.57**	**1318**	**380**
**Pallidum (RH)**	**-0.48**	**-0.45**	**0.09**	**-5.12**	**-0.66 - -0.3**	**3.07x10-7**	**5.48x10-7**	**42.81**	**1340**	**386**
**Thalamus (RH)**	**-0.45**	**-0.46**	**0.12**	**-3.67**	**-0.68 - -0.21**	**2.40x10-4**	**8.12x10-5**	**66.86**	**1346**	**386**
**Putamen (LH)**	**-0.40**	**-0.39**	**0.09**	**-4.56**	**-0.58 - -0.23**	**5.12x10-6**	**1.07x10-6**	**36.26**	**1286**	**382**
**Pallidum (LH)**	-0.31	-0.27	0.15	-2.05	-0.61 - -0.01	4.02x10-2	7.25x10-2	79.26	1266	370
**Putamen (RH)**	-0.30	-0.28	0.12	-2.43	-0.53 - -0.06	1.50x10-2	1.13x10-2	66.63	1320	384
**Amygdala (LH)**	-0.23	-0.22	0.11	-2.14	-0.43 - -0.02	3.25x10-2	2.03x10-2	55.48	1347	384
**Caudate (LH)**	-0.21	-0.18	0.13	-1.69	-0.46 - 0.03	9.10x10-2	1.22x10-1	69.11	1352	386
**Hippocampus (RH)**	-0.19	-0.20	0.15	-1.26	-0.48 - 0.1	2.06x10-1	1.59x10-1	79.00	1351	386
**Accumbens (RH)**	-0.15	-0.12	0.17	-0.93	-0.48 - 0.17	3.53x10-1	4.47x10-1	82.65	1330	385
**Accumbens (LH)**	-0.12	-0.10	0.12	-1.02	-0.35 - 0.11	3.10x10-1	4.36x10-1	63.58	1336	385
**Caudate (RH)**	-0.08	-0.06	0.13	-0.60	-0.32 - 0.17	5.47x10-1	5.87x10-1	69.80	1353	385
**Amygdala (RH)**	0.27	0.27	0.10	2.66	0.07 - 0.47	7.88x10-3	3.42x10-3	51.70	1321	370
**Right lateral ventricle**	**0.34**	**0.36**	**0.10**	**3.47**	**0.15 - 0.53**	**5.23x10-4**	**8.95x10-5**	**48.61**	**1352**	**386**
**Left lateral ventricle**	**0.45**	**0.47**	**0.10**	**4.61**	**0.26 - 0.64**	**4.00x10-6**	**1.96x10-7**	**48.10**	**1351**	**386**

Abbreviations: Beta, correlation beta; CI, confidence interval; I^2, heterogeneity index. All *p*-values are reported, uncorrected. For ease of comparison, Cohen's *d* estimates and *p*-values extracted using 3T data only are provided alongside Cohen's d estimates and *p*-values extracted from the full sample, combining 1.5T and 3T datasets. *P*-values that passed the Bonferroni-adjusted alpha level (p<1.49x10^{-4}) are highlighted in bold red (see Methods for statistical threshold determination). Brain regions reported as significant in the full sample, but not in the 3T sample, are highlighted in yellow.

Supplementary Table 12. Effect sizes for subcortical volume differences (Cohen's *d*) between healthy controls and the 'MTLE-R' phenotype; 3T field strength only.

Structure	Cohen's *d* (3T only)	Cohen's *d* (full sample)	SE	Z score	95% CI	*P* value (3T only)	*P* value (full sample)	I^2	Number of controls	Number of cases
**Hippocampus (RH)**	-1.90	-1.91	0.16	-11.76	-2.22 - -1.58	**6.10x10-32**	**6.36x10-37**	74.50	1239	312
**Thalamus (RH)**	-0.73	-0.73	0.12	-6.34	-0.96 - -0.51	**2.28x10-10**	**1.60x10-12**	58.38	1238	311
**Pallidum (RH)**	-0.48	-0.45	0.09	-5.40	-0.66 - -0.31	**6.71x10-8**	**3.96x10-7**	31.94	1231	308
**Putamen (RH)**	-0.48	-0.47	0.15	-3.28	-0.77 - -0.19	1.05x10-3	4.94x10-4	75.19	1211	313
**Thalamus (LH)**	-0.46	-0.47	0.23	-2.00	-0.9 - -0.01	4.57x10-2	2.41x10-2	89.88	1208	292
**Pallidum (LH)**	-0.33	-0.30	0.13	-2.63	-0.58 - -0.09	8.48x10-3	1.43x10-2	65.47	1130	295
**Putamen (LH)**	-0.32	-0.30	0.14	-2.29	-0.59 - -0.05	2.20x10-2	1.72x10-2	72.53	1157	306
**Caudate (LH)**	-0.20	-0.20	0.10	-1.96	-0.39 - 0	5.05x10-2	3.14x10-2	47.20	1244	313
**Hippocampus (LH)**	-0.17	-0.17	0.10	-1.71	-0.36 - 0.02	8.80x10-2	5.57x10-2	43.74	1230	313
**Caudate (RH)**	-0.17	-0.19	0.11	-1.57	-0.37 - 0.04	1.16x10-1	5.74x10-2	51.82	1245	313
**Accumbens (RH)**	-0.05	-0.03	0.14	-0.33	-0.33 - 0.23	7.41x10-1	8.45x10-1	74.25	1222	312
**Accumbens (LH)**	0.04	0.05	0.10	0.41	-0.15 - 0.23	6.83x10-1	5.58x10-1	45.48	1228	312
**Amygdala (RH)**	0.05	0.03	0.13	0.42	-0.2 - 0.3	6.77x10-1	8.25x10-1	67.36	1213	307
**Amygdala (LH)**	0.24	0.23	0.09	2.67	0.06 - 0.41	7.63x10-3	4.24x10-3	32.22	1239	310
**Left lateral ventricle**	0.37	0.39	0.09	4.16	0.2 - 0.54	**3.14x10-5**	**1.52x10-6**	32.99	1244	314
**Right lateral ventricle**	0.43	0.44	0.07	6.47	0.3 - 0.56	**9.58x10-11**	**6.57x10-12**	0.01	1245	314

Abbreviations: Beta, correlation beta; CI, confidence interval; I^2, heterogeneity index. All *p*-values are reported, uncorrected. For ease of comparison, Cohen's *d* estimates and *p*-values extracted using 3T data only are provided alongside Cohen's d estimates and *p*-values extracted from the full sample, combining 1.5T and 3T datasets. *P*-values that passed the Bonferroni-adjusted alpha level (p<1.49x10^{-4}) are highlighted in bold red (see Methods for statistical threshold determination). Brain regions reported as significant in the full sample, but not in the 3T sample, are highlighted in yellow.

Supplementary Table 13. Effect sizes for subcortical volume differences (Cohen's *d*) between healthy controls and the 'IGE' phenotype; 3T field strength only.

Structure	Cohen's *d* (3T only)	Cohen's *d* (full sample)	SE	Z score	95% CI	*P* value (3T only)	*P* value (full sample)	I^2	Number of controls	Number of cases
**Thalamus (LH)**	-0.39	-0.40	0.14	-2.88	-0.66 - -0.13	3.97x10-3	2.99x10-3	74.72	1162	359
**Thalamus (RH)**	**-0.39**	**-0.40**	**0.09**	**-4.29**	-0.57 - -0.21	**1.76x10-5**	**3.60x10-6**	**44.13**	**1191**	**362**
**Pallidum (RH)**	**-0.37**	**-0.35**	**0.10**	**-3.85**	-0.56 - -0.18	**1.16x10-4**	**3.37x10-4**	**49.58**	**1185**	**362**
**Hippocampus (LH)**	-0.32	-0.32	0.14	-2.35	-0.59 - -0.05	1.88x10-2	1.90x10-2	74.77	1185	362
**Putamen (LH)**	-0.29	-0.28	0.09	-3.32	-0.47 - -0.12	9.08x10-4	1.32x10-3	39.68	1111	352
**Putamen (RH)**	-0.26	-0.23	0.10	-2.50	-0.46 - -0.06	1.23x10-2	3.93x10-2	55.10	1167	357
**Caudate (LH)**	-0.25	-0.25	0.08	-3.28	-0.4 - -0.1	1.02x10-3	1.09x10-3	24.56	1199	361
**Hippocampus (RH)**	-0.24	-0.25	0.14	-1.75	-0.51 - 0.03	7.94x10-2	6.35x10-2	75.36	1195	360
**Caudate (RH)**	-0.20	-0.20	0.08	-2.42	-0.36 - -0.04	1.55x10-2	1.53x10-2	33.65	1200	362
**Pallidum (LH)**	-0.19	-0.16	0.18	-1.07	-0.53 - 0.16	2.85x10-1	3.85x10-1	84.47	1084	343
**Accumbens (RH)**	-0.16	-0.15	0.09	-1.77	-0.34 - 0.02	7.74x10-2	8.81x10-2	43.65	1180	363
**Accumbens (LH)**	-0.14	-0.13	0.10	-1.37	-0.33 - 0.06	1.72x10-1	1.78x10-1	53.61	1185	363
**Amygdala (RH)**	-0.14	-0.12	0.09	-1.53	-0.31 - 0.04	1.25x10-1	1.78x10-1	41.36	1168	362
**Amygdala (LH)**	-0.05	-0.05	0.13	-0.36	-0.31 - 0.22	7.17x10-1	7.10x10-1	73.86	1195	352
**Left lateral ventricle**	0.23	0.24	0.09	2.62	0.06 - 0.4	8.87x10-3	5.20x10-3	40.50	1197	359
**Right lateral ventricle**	0.26	0.27	0.09	3.04	0.09 - 0.43	2.36x10-3	9.23x10-4	36.71	1197	361

Abbreviations: Beta, correlation beta; CI, confidence interval; I^2, heterogeneity index. All *p*-values are reported, uncorrected. For ease of comparison, Cohen's *d* estimates and *p*-values extracted using 3T data only are provided alongside Cohen's d estimates and *p*-values extracted from the full sample, combining 1.5T and 3T datasets. *P*-values that passed the Bonferroni-adjusted alpha level ($p<1.49x10^{-4}$) are highlighted in bold red (see Methods for statistical threshold determination). Brain regions reported as significant in the 3T sample, but not in the full sample, are highlighted in green.

Supplementary Table 14. Effect sizes for subcortical volume differences (Cohen's *d*) between healthy controls and the 'all-other-epilepsies' phenotype; 3T field strength only.

Structure	Cohen's *d* (3T only)	Cohen's *d* (full sample)	SE	Z score	95% CI	*P* value (3T only)	*P* value (full sample)	I^2	Number of controls	Number of cases
**Thalamus (RH)**	-0.299	-0.305	0.047	-6.334	-0.392 - -0.207	**2.39x10^{-10}**	**7.92x10^{-11}**	4.565	1427	986
**Pallidum (RH)**	-0.225	-0.235	0.058	-3.886	-0.338 - -0.111	**1.02x10-4**	**8.07x10^{-5}**	31.140	1421	964
**Pallidum (LH)**	-0.209	-0.208	0.091	-2.297	-0.388 - -0.031	2.16x10-2	2.13x10-2	70.101	1320	891
**Thalamus (LH)**	-0.204	-0.197	0.081	-2.533	-0.363 - -0.046	1.13x10-2	1.32x10-2	63.636	1398	981
**Putamen (LH)**	-0.184	-0.193	0.080	-2.293	-0.342 - -0.027	2.18x10-2	1.82x10-2	62.129	1347	943
**Putamen (RH)**	-0.163	-0.166	0.083	-1.964	-0.326 - 0	4.96x10-2	4.57x10-2	65.867	1401	981
**Caudate (LH)**	-0.063	-0.070	0.059	-1.053	-0.179 - 0.054	2.93x10-1	2.34x10-1	35.153	1434	983
**Hippocampus (RH)**	-0.052	-0.041	0.048	-1.069	-0.147 - 0.043	2.85x10-1	3.82x10-1	8.116	1429	979
**Hippocampus (LH)**	-0.024	-0.021	0.058	-0.413	-0.137 - 0.09	6.80x10-1	7.08x10-1	31.517	1420	976
**Caudate (RH)**	0.003	-0.009	0.062	0.056	-0.119 - 0.125	9.55x10-1	8.81x10-1	40.460	1435	985
**Accumbens (LH)**	0.010	-0.011	0.092	0.107	-0.17 - 0.189	9.14x10-1	9.12x10-1	71.895	1418	984
**Accumbens (RH)**	0.024	0.016	0.091	0.263	-0.154 - 0.202	7.92x10-1	8.64x10-1	71.420	1412	985
**Left lateral ventricle**	0.196	0.198	0.046	4.258	0.106 - 0.286	**2.06x10-5**	**1.23x10^{-5}**	0.983	1433	985
**Right lateral ventricle**	0.209	0.212	0.047	4.483	0.118 - 0.3	**7.35x10-6**	**4.62x10^{-6}**	2.965	1434	985
**Amygdala (RH)**	0.212	0.218	0.061	3.467	0.092 - 0.331	**5.26x10-4**	**1.46x10^{-4}**	37.216	1403	977
**Amygdala (LH)**	0.322	0.327	0.067	4.810	0.191 - 0.453	**1.51x10-6**	**5.05x10^{-7}**	47.145	1429	986

Abbreviations: Beta, correlation beta; CI, confidence interval; I^2, heterogeneity index. All *p*-values are reported, uncorrected. For ease of comparison, Cohen's *d* estimates and *p*-values extracted using 3T data only are provided alongside Cohen's d estimates and *p*-values extracted from the full sample, combining 1.5T and 3T datasets. *P*-values that passed the Bonferroni-adjusted alpha level (p<1.49x10^{-4}) are highlighted in bold red (see Methods for statistical threshold determination). Brain regions reported as significant in the full sample, but not in the 3T sample, are highlighted in yellow.

Supplementary Table 15. Effect sizes for cortical thickness differences (Cohen's *d*) between healthy controls and the 'all-epilepsies' phenotype; 3T field strength only.

Structure	Cohen's *d* (3T only)	Cohen's *d* (full sample)	SE	Z score	95% CI	*P* value (3T only)	*P* value (full sample)	I^2	Number of controls	Number of cases
R_precentral	-0.41	-0.40	0.04	-9.06	-0.49 - -0.32	**1.34x10-19**	**8.85x10-20**	27.62	1583	1987
L_precentral	-0.40	-0.38	0.04	-9.31	-0.48 - -0.32	**1.31x10-20**	**1.82x10-18**	21.96	1579	1991
L_caudalmiddlefrontal	-0.32	-0.32	0.04	-8.05	-0.4 - -0.25	**8.22x10-16**	**2.11x10-15**	14.37	1584	1994
R_superiorparietal	-0.32	-0.31	0.10	-3.28	-0.51 - -0.13	1.04x10-3	8.13x10-4	85.05	1587	1991
R_caudalmiddlefrontal	-0.31	-0.31	0.05	-5.84	-0.42 - -0.21	**5.09x10-9**	**2.09x10-9**	48.45	1587	1992
L_paracentral	-0.31	-0.31	0.07	-4.52	-0.45 - -0.18	**6.26x10-6**	**2.05x10-6**	69.37	1584	1994
R_paracentral	-0.31	-0.32	0.06	-5.45	-0.42 - -0.2	**5.13x10-8**	**2.19x10-9**	54.17	1588	1992
L_superiorparietal	-0.29	-0.29	0.08	-3.68	-0.45 - -0.14	2.34x10-4	**1.78x10-4**	77.22	1581	1991
R_superiorfrontal	-0.28	-0.27	0.06	-4.67	-0.4 - -0.17	**2.97x10-6**	**4.49x10-6**	60.20	1584	1991
L_superiorfrontal	-0.28	-0.28	0.06	-4.99	-0.4 - -0.17	**6.00x10-7**	**1.51x10-7**	54.46	1583	1992
R_precuneus	-0.28	-0.28	0.07	-4.06	-0.42 - -0.14	**4.90x10-5**	**2.70x10-5**	69.21	1588	1988
L_entorhinal	-0.26	-0.26	0.06	-4.04	-0.38 - -0.13	**5.44x10-5**	**2.04x10-5**	56.86	1341	1663
L_precuneus	-0.25	-0.25	0.09	-2.85	-0.42 - -0.08	4.30x10-3	2.66x10-3	81.09	1583	1992
L_supramarginal	-0.24	-0.23	0.06	-3.98	-0.36 - -0.12	**6.99x10-5**	**9.87x10-5**	59.40	1540	1898
R_supramarginal	-0.23	-0.22	0.06	-4.07	-0.34 - -0.12	**4.65x10-5**	**5.24x10-5**	53.44	1531	1906
R_rostralmiddlefrontal	-0.22	-0.20	0.06	-4.01	-0.33 - -0.11	**6.01x10-5**	5.94x10-4	52.28	1588	1991
L_temporalpole	-0.21	-0.20	0.06	-3.44	-0.34 - -0.09	5.76x10-4	7.49x10-4	61.85	1578	1990
R_cuneus	-0.21	-0.20	0.04	-5.35	-0.29 - -0.13	**8.88x10-8**	**9.68x10-8**	11.43	1586	1990
R_parstriangularis	-0.21	-0.20	0.04	-5.70	-0.28 - -0.14	**1.22x10-8**	**4.25x10-8**	3.05	1586	1991
L_middletemporal	-0.21	-0.20	0.07	-2.91	-0.35 - -0.07	3.65x10-3	4.01x10-3	70.19	1483	1893
L_inferiorparietal	-0.21	-0.20	0.07	-2.87	-0.35 - -0.07	4.05x10-3	5.29x10-3	71.93	1573	1985
L_parstriangularis	-0.20	-0.19	0.05	-3.92	-0.31 - -0.1	**8.89x10-5**	**1.29x10-4**	45.84	1584	1993

**L_lateraloccipital**	-0.19	-0.19	0.09	-2.05	-0.38 - -0.01	4.00x10-2	3.58x10-2	83.97	1584	1993
**R_lateraloccipital**	-0.19	-0.20	0.07	-2.75	-0.33 - -0.06	5.99x10-3	1.77x10-3	70.64	1587	1991
**L_transversetemporal**	-0.19	-0.19	0.04	-4.27	-0.28 - -0.1	**1.97x10-5**	**1.05x10-5**	28.81	1581	1994
**L_fusiform**	-0.19	-0.19	0.06	-2.98	-0.31 - -0.07	2.86x10-3	2.10x10-3	63.91	1581	1991
**R_temporalpole**	-0.18	-0.18	0.05	-3.45	-0.29 - -0.08	5.63x10-4	5.51x10-4	48.72	1584	1986
**R_inferiorparietal**	-0.18	-0.17	0.08	-2.43	-0.33 - -0.04	1.52x10-2	2.15x10-2	74.50	1577	1978
**R_transversetemporal**	-0.18	-0.18	0.05	-3.99	-0.27 - -0.09	**6.67x10-5**	**2.81x10-5**	30.17	1588	1992
**L_lingual**	-0.18	-0.18	0.09	-2.01	-0.35 - 0	4.45x10-2	3.50x10-2	81.92	1583	1994
**R_parsopercularis**	-0.17	-0.18	0.04	-4.52	-0.25 - -0.1	**6.15x10-6**	**6.48x10-7**	8.20	1586	1992
**L_superiortemporal**	-0.17	-0.18	0.08	-2.03	-0.34 - -0.01	4.19x10-2	2.62x10-2	78.87	1469	1875
**L_parsorbitalis**	-0.17	-0.15	0.05	-3.70	-0.26 - -0.08	2.14x10-4	2.37x10-3	31.23	1584	1992
**L_parsopercularis**	-0.16	-0.15	0.06	-2.78	-0.28 - -0.05	5.47x10-3	7.12x10-3	57.82	1582	1992
**L_rostralmiddlefrontal**	-0.16	-0.15	0.06	-2.57	-0.29 - -0.04	1.02x10-2	2.15x10-2	63.69	1583	1990
**R_parsorbitalis**	-0.16	-0.14	0.05	-3.13	-0.26 - -0.06	1.75x10-3	8.27x10-3	45.14	1588	1990
**R_lingual**	-0.16	-0.16	0.05	-3.07	-0.26 - -0.06	2.14x10-3	8.14x10-4	43.24	1587	1989
**R_entorhinal**	-0.15	-0.16	0.06	-2.58	-0.26 - -0.04	9.77x10-3	4.24x10-3	44.89	1319	1636
**L_cuneus**	-0.14	-0.16	0.10	-1.42	-0.33 - 0.05	1.56x10-1	7.27x10-2	85.10	1582	1994
**L_postcentral**	-0.14	-0.13	0.09	-1.48	-0.32 - 0.05	1.39x10-1	1.67x10-1	83.75	1581	1989
**R_lateralorbitofrontal**	-0.13	-0.11	0.05	-2.47	-0.24 - -0.03	1.34x10-2	3.87x10-2	49.86	1585	1990
**R_middletemporal**	-0.13	-0.12	0.04	-3.26	-0.21 - -0.05	1.12x10-3	3.02x10-3	14.01	1577	1983
**R_pericalcarine**	-0.13	-0.13	0.05	-2.38	-0.23 - -0.02	1.72x10-2	9.15x10-3	47.12	1588	1989
**R_frontalpole**	-0.12	-0.11	0.05	-2.46	-0.22 - -0.02	1.39x10-2	2.37x10-2	40.44	1588	1990
**L_pericalcarine**	-0.12	-0.13	0.09	-1.31	-0.3 - 0.06	1.91x10-1	1.43x10-1	83.50	1584	1992
**R_superiortemporal**	-0.12	-0.12	0.06	-1.93	-0.24 - 0	5.32x10-2	3.84x10-2	60.72	1491	1893
**R_postcentral**	-0.12	-0.12	0.11	-1.13	-0.33 - 0.09	2.57x10-1	2.49x10-1	87.35	1582	1990
**L_parahippocampal**	-0.12	-0.10	0.05	-2.49	-0.21 - -0.02	1.28x10-2	1.83x10-2	32.14	1571	1983
**R_fusiform**	-0.11	-0.12	0.05	-2.21	-0.2 - -0.01	2.74x10-2	8.56x10-3	36.43	1588	1990

**R_parahippocampal**	-0.10	-0.09	0.04	-2.87	-0.17 - -0.03	4.17x10-3	6.95x10-3	0.01	1584	1986
**L_posteriorcingulate**	-0.10	-0.08	0.04	-2.20	-0.18 - -0.01	2.78x10-2	6.01x10-2	27.50	1584	1992
**R_posteriorcingulate**	-0.10	-0.07	0.06	-1.61	-0.21 - 0.02	1.06x10-1	2.65x10-1	59.49	1588	1989
**L_bankssts**	-0.10	-0.09	0.08	-1.15	-0.26 - 0.07	2.48x10-1	2.45x10-1	77.56	1521	1865
**L_frontalpole**	-0.09	-0.08	0.05	-1.80	-0.18 - 0.01	7.17x10-2	1.04x10-1	34.88	1582	1991
**L_inferiortemporal**	-0.08	-0.09	0.06	-1.37	-0.2 - 0.04	1.70x10-1	1.37x10-1	58.70	1583	1988
**L_lateralorbitofrontal**	-0.08	-0.07	0.05	-1.64	-0.17 - 0.02	1.01x10-1	1.46x10-1	36.45	1579	1993
**R_medialorbitofrontal**	-0.07	-0.05	0.04	-2.00	-0.14 - 0	4.55x10-2	1.26x10-1	0.00	1570	1958
**R_inferiortemporal**	-0.06	-0.07	0.04	-1.79	-0.13 - 0.01	7.34x10-2	8.36x10-2	0.00	1586	1990
**L_isthmuscingulate**	-0.05	-0.05	0.04	-1.41	-0.13 - 0.02	1.59x10-1	1.72x10-1	6.35	1583	1990
**R_bankssts**	-0.04	-0.06	0.07	-0.65	-0.18 - 0.09	5.17x10-1	3.66x10-1	67.78	1558	1956
**R_insula**	-0.03	-0.02	0.05	-0.63	-0.12 - 0.06	5.27x10-1	6.42x10-1	35.04	1549	1965
**L_rostralanteriorcingulate**	-0.02	-0.01	0.06	-0.31	-0.14 - 0.1	7.55x10-1	8.34x10-1	60.56	1579	1981
**L_medialorbitofrontal**	0.00	0.01	0.04	-0.07	-0.07 - 0.07	9.43x10-1	8.82x10-1	0.00	1566	1963
**R_caudalanteriorcingulate**	0.00	0.01	0.06	0.03	-0.12 - 0.13	9.78x10-1	8.24x10-1	64.73	1586	1989
**R_isthmuscingulate**	0.02	0.01	0.05	0.29	-0.09 - 0.12	7.70x10-1	8.56x10-1	44.84	1585	1990
**L_insula**	0.03	0.01	0.05	0.61	-0.06 - 0.12	5.44x10-1	7.73x10-1	33.62	1544	1959
**L_caudalanteriorcingulate**	0.05	0.04	0.04	1.24	-0.03 - 0.14	2.14x10-1	3.59x10-1	24.59	1579	1978
**R_rostralanteriorcingulate**	0.07	0.09	0.05	1.47	-0.03 - 0.17	1.43x10-1	8.64x10-2	43.23	1586	1988

Abbreviations: Beta, correlation beta; CI, confidence interval; I^2, heterogeneity index. All *p*-values are reported, uncorrected. See Supplementary Table 3 for a list of cortical abbreviations. For ease of comparison, Cohen's *d* estimates and *p*-values extracted using 3T data only are provided alongside Cohen's d estimates and *p*-values extracted from the full sample, combining 1.5T and 3T datasets. *P*-values that passed the Bonferroni-adjusted alpha level (p<1.49x10^{-4}) are highlighted in bold red (see Methods for statistical threshold determination). Brain regions reported as significant in the full sample, but not in the 3T sample, are highlighted in yellow. Brain regions reported as significant in the 3T sample, but not in the full sample, are highlighted in green.

Supplementary Table 16. Effect sizes for cortical thickness differences (Cohen's *d*) between healthy controls and the 'MTLE-L' phenotype; 3T field strength only.

Structure	Cohen's *d* (3T only)	Cohen's *d* (full sample)	SE	Z score	95% CI	*P* value (3T only)	*P* value (full sample)	I^2	Number of controls	Number of cases
**L_precuneus**	-0.60	-0.40	0.13	-4.47	-0.86 - -0.34	**7.90x10-6**	**7.35x10-5**	72.03	1277	384
**R_precentral**	-0.55	-0.49	0.07	-8.01	-0.68 - -0.41	**1.17x10-15**	**2.37x10-10**	7.52	1279	384
**L_superiorparietal**	-0.54	-0.40	0.14	-3.75	-0.81 - -0.26	**1.75x10-4**	9.17x10-4	75.60	1276	384
**R_precuneus**	-0.53	-0.40	0.10	-5.17	-0.73 - -0.33	**2.33x10-7**	**5.16x10-6**	51.38	1282	384
**R_superiorparietal**	-0.53	-0.40	0.16	-3.33	-0.85 - -0.22	8.65x10-4	2.97x10-3	80.80	1281	384
**L_precentral**	-0.50	-0.40	0.08	-6.47	-0.66 - -0.35	**1.01x10-10**	**8.64x10-9**	21.45	1273	384
**R_caudalmiddlefrontal**	-0.48	-0.40	0.09	-5.48	-0.65 - -0.31	**4.28x10-8**	**3.61x10-7**	35.26	1282	384
**L_superiorfrontal**	-0.46	-0.40	0.07	-6.48	-0.59 - -0.32	**9.43x10-11**	**1.02x10-11**	10.13	1277	384
**L_paracentral**	-0.46	-0.40	0.10	-4.54	-0.67 - -0.26	**5.55x10-6**	**1.61x10-5**	51.94	1278	384
**L_entorhinal**	-0.44	-0.40	0.08	-5.77	-0.58 - -0.29	**7.78x10-9**	**7.35x10-10**	0.00	1041	278
**L_caudalmiddlefrontal**	-0.43	-0.40	0.06	-6.69	-0.56 - -0.31	**2.18x10-11**	**7.07x10-9**	2.86	1278	384
**L_superiortemporal**	-0.43	-0.40	0.12	-3.46	-0.67 - -0.18	5.34x10-4	3.11x10-4	65.68	1164	361
**R_superiorfrontal**	-0.41	-0.40	0.06	-6.58	-0.53 - -0.29	**4.79x10-11**	**1.44x10-9**	0.00	1279	384
**L_fusiform**	-0.40	-0.40	0.06	-6.48	-0.53 - -0.28	**9.44x10-11**	**2.19x10-7**	0.00	1276	384
**R_paracentral**	-0.40	-0.40	0.08	-5.17	-0.56 - -0.25	**2.38x10-7**	**5.14x10-7**	22.54	1282	384
**L_lingual**	-0.39	-0.40	0.14	-2.83	-0.66 - -0.12	4.60x10-3	7.89x10-3	74.18	1277	384
**L_postcentral**	-0.38	-0.40	0.14	-2.76	-0.65 - -0.11	5.82x10-3	1.67x10-2	73.88	1275	383
**R_postcentral**	-0.38	-0.40	0.14	-2.70	-0.66 - -0.11	6.92x10-3	1.83x10-2	75.74	1278	384
**L_lateraloccipital**	-0.36	-0.40	0.15	-2.43	-0.66 - -0.07	1.52x10-2	3.88x10-2	78.39	1278	384
**L_temporalpole**	-0.35	-0.40	0.06	-5.66	-0.48 - -0.23	**1.52x10-8**	**3.33x10-6**	0.35	1276	383
**R_supramarginal**	-0.35	-0.40	0.09	-4.11	-0.52 - -0.19	**3.89x10-5**	6.53x10-4	32.23	1226	372

**L_supramarginal**	-0.35	-0.40	0.09	-3.71	-0.53 - -0.16	2.05x10-4	2.95x10-3	42.27	1234	367
**R_inferiorparietal**	-0.35	-0.40	0.11	-3.21	-0.57 - -0.14	1.34x10-3	1.61x10-2	58.84	1271	383
**L_parahippocampal**	-0.34	-0.40	0.06	-5.45	-0.46 - -0.22	**4.93x10-8**	**3.95x10-5**	0.00	1269	382
**L_middletemporal**	-0.32	-0.40	0.10	-3.17	-0.52 - -0.12	1.53x10-3	2.50x10-3	49.49	1178	360
**R_rostralmiddlefrontal**	-0.32	-0.40	0.08	-3.83	-0.48 - -0.16	**1.29x10-4**	1.07x10-2	30.57	1282	384
**R_parstriangularis**	-0.31	-0.40	0.06	-4.97	-0.43 - -0.19	**6.85x10-7**	**2.16x10-6**	0.00	1280	384
**L_parstriangularis**	-0.31	-0.40	0.10	-3.21	-0.51 - -0.12	1.35x10-3	2.30x10-3	48.35	1278	383
**R_lateraloccipital**	-0.31	-0.40	0.11	-2.71	-0.53 - -0.08	6.68x10-3	3.64x10-2	61.07	1281	384
**L_inferiorparietal**	-0.30	-0.40	0.13	-2.31	-0.56 - -0.05	2.09x10-2	6.13x10-2	71.40	1267	382
**L_rostralmiddlefrontal**	-0.27	-0.40	0.10	-2.62	-0.47 - -0.07	8.91x10-3	4.41x10-2	52.05	1278	384
**L_parsopercularis**	-0.25	-0.40	0.11	-2.31	-0.46 - -0.04	2.10x10-2	3.50x10-2	57.43	1276	383
**L_inferiortemporal**	-0.24	-0.40	0.08	-2.92	-0.4 - -0.08	3.45x10-3	5.65x10-3	29.11	1277	384
**R_cuneus**	-0.22	-0.40	0.08	-2.87	-0.37 - -0.07	4.09x10-3	8.02x10-3	21.19	1280	384
**L_parsorbitalis**	-0.21	-0.40	0.09	-2.42	-0.38 - -0.04	1.56x10-2	1.02x10-1	36.48	1278	384
**R_posteriorcingulate**	-0.21	-0.40	0.09	-2.37	-0.38 - -0.04	1.80x10-2	1.23x10-1	36.79	1282	383
**R_fusiform**	-0.20	-0.40	0.06	-3.28	-0.33 - -0.08	1.03x10-3	1.00x10-3	0.00	1282	384
**R_superiortemporal**	-0.19	-0.40	0.06	-2.93	-0.31 - -0.06	3.36x10-3	7.44x10-3	0.00	1185	370
**L_pericalcarine**	-0.19	-0.40	0.13	-1.42	-0.44 - 0.07	1.55x10-1	1.05x10-1	71.70	1278	383
**R_lateralorbitofrontal**	-0.19	-0.40	0.11	-1.71	-0.4 - 0.03	8.74x10-2	2.40x10-1	58.53	1281	384
**R_parsopercularis**	-0.18	-0.40	0.09	-2.00	-0.35 - 0	4.53x10-2	5.21x10-2	38.75	1281	384
**R_parsorbitalis**	-0.18	-0.40	0.08	-2.16	-0.34 - -0.02	3.06x10-2	7.20x10-2	29.91	1282	384
**R_lingual**	-0.18	-0.40	0.11	-1.73	-0.39 - 0.02	8.36x10-2	1.14x10-1	55.88	1282	384
**L_cuneus**	-0.18	-0.40	0.14	-1.28	-0.46 - 0.1	2.00x10-1	1.34x10-1	75.60	1276	384
**R_transversetemporal**	-0.16	-0.40	0.07	-2.19	-0.3 - -0.02	2.87x10-2	1.72x10-2	15.98	1282	384
**L_transversetemporal**	-0.16	-0.40	0.07	-2.40	-0.3 - -0.03	1.65x10-2	3.58x10-2	8.49	1275	384
**R_pericalcarine**	-0.16	-0.40	0.08	-1.94	-0.32 - 0	5.22x10-2	3.73x10-2	27.98	1282	383
**R_parahippocampal**	-0.16	-0.40	0.09	-1.81	-0.34 - 0.01	7.08x10-2	1.46x10-1	39.75	1278	384

**R_frontalpole**	-0.14	-0.40	0.07	-2.18	-0.27 - -0.01	2.95x10-2	9.53x10-2	4.54	1282	383
**L_bankssts**	-0.13	-0.40	0.12	-1.10	-0.37 - 0.1	2.72x10-1	3.72x10-1	64.52	1216	357
**R_medialorbitofrontal**	-0.12	-0.40	0.06	-1.95	-0.24 - 0	5.14x10-2	2.23x10-1	0.00	1282	382
**R_bankssts**	-0.11	-0.40	0.11	-1.03	-0.33 - 0.1	3.04x10-1	2.51x10-1	59.72	1252	378
**L_frontalpole**	-0.10	-0.40	0.06	-1.54	-0.22 - 0.03	1.24x10-1	2.96x10-1	0.00	1277	382
**L_lateralorbitofrontal**	-0.10	-0.40	0.09	-1.14	-0.27 - 0.07	2.54x10-1	4.11x10-1	34.08	1277	384
**R_middletemporal**	-0.10	-0.40	0.08	-1.16	-0.26 - 0.07	2.46x10-1	4.13x10-1	31.24	1271	382
**R_entorhinal**	-0.08	-0.40	0.10	-0.86	-0.28 - 0.11	3.90x10-1	3.10x10-1	33.51	1015	273
**R_caudalanteriorcingulate**	-0.08	-0.40	0.11	-0.72	-0.3 - 0.14	4.71x10-1	5.76x10-1	60.66	1280	383
**R_inferiortemporal**	-0.05	-0.40	0.08	-0.59	-0.2 - 0.11	5.54x10-1	5.22x10-1	21.06	1280	384
**L_isthmuscingulate**	-0.05	-0.40	0.08	-0.56	-0.21 - 0.12	5.74x10-1	7.91x10-1	29.51	1277	383
**L_posteriorcingulate**	-0.05	-0.40	0.06	-0.76	-0.17 - 0.07	4.46x10-1	8.45x10-1	0.00	1278	383
**R_temporalpole**	-0.04	-0.40	0.06	-0.67	-0.16 - 0.08	5.04x10-1	5.86x10-1	0.00	1280	383
**R_isthmuscingulate**	-0.03	-0.40	0.08	-0.41	-0.2 - 0.13	6.80x10-1	9.31x10-1	29.45	1279	383
**L_medialorbitofrontal**	0.00	-0.40	0.08	0.06	-0.16 - 0.17	9.55x10-1	6.13x10-1	28.96	1278	384
**L_rostralanteriorcingulate**	0.03	-0.40	0.08	0.34	-0.12 - 0.17	7.35x10-1	3.88x10-1	18.43	1276	383
**R_insula**	0.04	-0.40	0.06	0.70	-0.08 - 0.17	4.83x10-1	2.54x10-1	0.00	1257	377
**R_rostralanteriorcingulate**	0.16	-0.40	0.06	2.56	0.04 - 0.28	1.03x10-2	8.80x10-4	0.00	1282	383
**L_insula**	0.18	-0.40	0.08	2.27	0.02 - 0.33	2.33x10-2	5.31x10-2	22.78	1254	374
**L_caudalanteriorcingulate**	0.19	-0.40	0.06	3.11	0.07 - 0.32	1.89x10-3	2.55x10-3	0.00	1277	383

Abbreviations: Beta, correlation beta; CI, confidence interval; I^2, heterogeneity index. All *p*-values are reported, uncorrected. See Supplementary Table 3 for a list of cortical abbreviations. For ease of comparison, Cohen's *d* estimates and *p*-values extracted using 3T data only are provided alongside Cohen's d estimates and *p*-values extracted from the full sample, combining 1.5T and 3T datasets. *P*-values that passed the Bonferroni-adjusted alpha level ($p<1.49x10^{-4}$) are highlighted in bold red (see Methods for statistical threshold determination). Brain regions reported as significant in the 3T sample, but not in the full sample, are highlighted in green

Supplementary Table 17. Effect sizes for cortical thickness differences (Cohen's *d*) between healthy controls and the 'MTLE-R' phenotype; 3T field strength only.

Structure	Cohen's *d* (3T only)	Cohen's *d* (full sample)	SE	Z score	95% CI	*P* value (3T only)	*P* value (full sample)	I^2	Number of controls	Number of cases
**R_precentral**	**-0.54**	**-0.52**	**0.09**	**-6.11**	-0.72 - -0.37	**9.73x10-10**	**1.25x10-9**	**33**	**1246**	**313**
**L_paracentral**	**-0.52**	**-0.5**	**0.11**	**-4.65**	-0.73 - -0.3	**3.35x10-6**	**7.67x10-7**	**56.09**	**1245**	**314**
R_superiorparietal	-0.51	-0.49	0.19	-2.73	-0.87 - -0.14	6.35x10-3	5.03x10-3	84.78	1248	314
R_precuneus	-0.48	-0.46	0.13	-3.69	-0.74 - -0.23	2.24x10-4	1.87x10-4	68.78	1249	314
**L_precentral**	**-0.46**	**-0.42**	**0.09**	**-5.33**	-0.63 - -0.29	**1.01x10-7**	**4.31x10-6**	**29.93**	**1240**	**314**
**R_paracentral**	**-0.43**	**-0.42**	**0.07**	**-6.36**	-0.57 - -0.3	**1.99x10-10**	**6.24x10-11**	**2.25**	**1249**	**314**
L_superiorparietal	-0.42	-0.4	0.13	-3.21	-0.68 - -0.16	1.33x10-3	1.27x10-3	69.45	1244	314
L_precuneus	-0.41	-0.38	0.12	-3.47	-0.64 - -0.18	5.19x10-4	6.47x10-4	61.69	1244	314
**R_lateraloccipital**	-0.4	-0.37	0.1	-3.94	-0.59 - -0.2	**8.08x10-5**	**1.79x10-4**	46.98	1248	314
**L_supramarginal**	-0.39	-0.36	0.1	-3.85	-0.68 - -0.1	**1.18x10-4**	2.25x10-4	45.38	1201	297
L_lingual	-0.39	-0.38	0.15	-2.63	-0.59 - -0.19	8.47x10-3	5.22x10-3	75.99	1244	314
L_postcentral	-0.39	-0.33	0.15	-2.61	-0.68 - -0.1	9.02x10-3	2.43x10-2	76.13	1242	314
R_postcentral	-0.38	-0.36	0.16	-2.32	-0.7 - -0.06	2.02x10-2	1.87x10-2	80.16	1245	314
**L_superiorfrontal**	**-0.36**	**-0.35**	**0.11**	**-3.32**	-0.57 - -0.15	**9.00x10-4**	**1.59x10-4**	**54.07**	**1244**	**313**
**R_caudalmiddlefrontal**	-0.34	-0.31	0.08	-3.99	-0.5 - -0.17	6.59x10-5	3.03x10-4	27.16	1249	314
R_superiorfrontal	-0.33	-0.3	0.12	-2.63	-0.57 - -0.08	8.61x10-3	1.14x10-2	65.76	1246	314
**L_transversetemporal**	**-0.31**	**-0.31**	**0.08**	**-3.92**	-0.51 - -0.12	**8.83x10-5**	**2.15x10-5**	**19.87**	**1242**	**314**
L_caudalmiddlefrontal	-0.31	-0.3	0.1	-2.99	-0.52 - -0.11	2.76x10-3	2.15x10-3	51.64	1245	314
R_supramarginal	-0.31	-0.27	0.1	-3.21	-0.52 - -0.1	1.32x10-3	6.33x10-3	43.79	1193	304
R_temporalpole	-0.31	-0.28	0.11	-2.91	-0.46 - -0.15	3.57x10-3	6.41x10-3	53.12	1246	313

L_lateraloccipital	-0.3	-0.25	0.12	-2.37	-0.54 - -0.05	1.77x10-2	4.25x10-2	66.01	1245	314
L_cuneus	-0.29	-0.29	0.15	-1.99	-0.58 - 0	4.62x10-2	3.58x10-2	75.65	1243	314
R_inferiorparietal	-0.29	-0.24	0.15	-1.97	-0.57 - 0	4.83x10-2	8.41x10-2	75.21	1238	314
**R_parsopercularis**	**-0.28**	**-0.27**	**0.08**	**-3.55**	-0.53 - -0.03	3.92x10-4	**1.45x10-4**	18.47	1248	314
L_inferiorparietal	-0.28	-0.24	0.13	-2.22	-0.43 - -0.12	2.66x10-2	6.04x10-2	67.18	1234	312
R_lingual	-0.27	-0.28	0.08	-3.42	-0.43 - -0.12	6.36x10-4	2.35x10-4	21.84	1248	314
**R_transversetemporal**	-0.25	-0.24	0.07	-3.84	-0.38 - -0.12	**1.21x10-4**	1.94x10-4	0	1249	314
R_cuneus	-0.25	-0.25	0.09	-2.74	-0.42 - -0.07	6.06x10-3	3.31x10-3	35.99	1247	314
L_pericalcarine	-0.25	-0.26	0.18	-1.4	-0.59 - 0.1	1.62x10-1	1.14x10-1	83.14	1245	314
L_bankssts	-0.25	-0.22	0.16	-1.55	-0.56 - 0.07	1.22x10-1	1.44x10-1	78.56	1184	301
L_parsorbitalis	-0.24	-0.19	0.09	-2.59	-0.42 - -0.06	9.65x10-3	5.27x10-2	37.37	1245	314
R_superiortemporal	-0.23	-0.23	0.11	-2.1	-0.45 - -0.02	3.54x10-2	2.21x10-2	54.73	1152	300
R_parsorbitalis	-0.23	-0.15	0.14	-1.62	-0.5 - 0.05	1.05x10-1	3.20x10-1	73.71	1249	314
R_pericalcarine	-0.21	-0.23	0.09	-2.38	-0.38 - -0.04	1.72x10-2	4.39x10-3	31.49	1249	314
R_middletemporal	-0.21	-0.16	0.11	-1.94	-0.41 - 0	5.28x10-2	1.41x10-1	52.94	1238	313
R_parahippocampal	-0.2	-0.18	0.07	-3.1	-0.33 - -0.08	1.92x10-3	6.05x10-3	0	1245	314
R_fusiform	-0.2	-0.17	0.07	-2.7	-0.44 - 0.04	6.91x10-3	2.51x10-2	12.01	1249	314
L_rostralmiddlefrontal	-0.2	-0.16	0.12	-1.65	-0.34 - -0.05	9.82x10-2	2.04x10-1	64.51	1245	313
R_parstriangularis	-0.19	-0.17	0.09	-2.23	-0.36 - -0.02	2.59x10-2	5.41x10-2	30.64	1247	313
R_bankssts	-0.19	-0.18	0.1	-1.85	-0.4 - 0.02	6.43x10-2	5.48x10-2	46.66	1219	309
L_parsopercularis	-0.19	-0.15	0.11	-1.76	-0.38 - 0.01	7.92x10-2	1.43x10-1	53.61	1243	314
R_entorhinal	-0.18	-0.18	0.11	-1.63	-0.39 - 0.04	1.02x10-1	7.23x10-2	38.73	982	223
L_fusiform	-0.17	-0.16	0.07	-2.5	-0.29 - -0.04	1.23x10-2	1.18x10-2	0	1243	313
L_temporalpole	-0.16	-0.15	0.08	-1.98	-0.38 - 0.06	4.74x10-2	4.16x10-2	19.92	1241	314
L_parstriangularis	-0.16	-0.15	0.11	-1.45	-0.37 - 0.06	1.47x10-1	1.43x10-1	56.6	1245	314
L_middletemporal	-0.16	-0.11	0.11	-1.45	-0.31 - 0	1.46x10-1	3.13x10-1	54.45	1146	299
R_posteriorcingulate	-0.16	-0.09	0.09	-1.7	-0.34 - 0.02	8.97x10-2	4.07x10-1	38.06	1249	313

R_inferiortemporal	-0.14	-0.11	0.11	-1.34	-0.35 - 0.07	1.80x10-1	2.77x10-1	53.07	1247	314
R_insula	-0.14	-0.11	0.11	-1.28	-0.35 - 0.07	2.01x10-1	2.89x10-1	54.97	1210	309
L_entorhinal	-0.13	-0.11	0.08	-1.62	-0.28 - 0.03	1.05x10-1	1.31x10-1	0	1008	231
L_superiortemporal	-0.13	-0.14	0.13	-0.95	-0.39 - 0.13	3.43x10-1	2.67x10-1	69.12	1132	294
L_frontalpole	-0.11	-0.08	0.09	-1.18	-0.28 - 0.07	2.37x10-1	3.71x10-1	35.53	1243	314
L_inferiortemporal	-0.08	-0.06	0.07	-1.19	-0.21 - 0.05	2.32x10-1	3.59x10-1	0	1244	314
L_posteriorcingulate	-0.08	-0.06	0.07	-1.26	-0.21 - 0.05	2.08x10-1	3.70x10-1	0.01	1245	314
R_frontalpole	-0.06	-0.03	0.1	-0.53	-0.26 - 0.15	5.96x10-1	7.76x10-1	52.12	1249	314
R_rostralmiddlefrontal	-0.06	-0.03	0.11	-0.51	-0.27 - 0.16	6.11x10-1	8.04x10-1	55.15	1249	314
L_lateralorbitofrontal	-0.05	-0.02	0.08	-0.59	-0.2 - 0.11	5.55x10-1	8.20x10-1	16.84	1241	314
L_insula	-0.01	-0.02	0.07	-0.09	-0.19 - 0.17	9.32x10-1	7.17x10-1	0	1207	308
L_parahippocampal	-0.01	-0.02	0.09	-0.1	-0.14 - 0.12	9.21x10-1	8.52x10-1	35.81	1236	313
R_lateralorbitofrontal	0	0.03	0.08	-0.02	-0.15 - 0.15	9.84x10-1	7.36x10-1	16.11	1246	314
L_rostralanteriorcingulate	0.01	0	0.1	0.07	-0.19 - 0.2	9.44x10-1	9.73x10-1	46.8	1240	311
R_caudalanteriorcingulate	0.03	0.06	0.13	0.23	-0.22 - 0.28	8.19x10-1	6.17x10-1	66.87	1247	313
L_caudalanteriorcingulate	0.04	-0.02	0.07	0.51	-0.1 - 0.17	6.07x10-1	7.77x10-1	4.78	1240	313
L_medialorbitofrontal	0.06	0.08	0.07	0.89	-0.07 - 0.19	3.72x10-1	1.98x10-1	0	1227	312
L_isthmuscingulate	0.09	0.08	0.08	1.03	-0.08 - 0.25	3.02x10-1	3.05x10-1	28.3	1244	314
R_medialorbitofrontal	0.11	0.15	0.09	1.26	-0.06 - 0.29	2.07x10-1	1.02x10-1	33.4	1231	312
R_isthmuscingulate	0.18	0.14	0.1	1.73	-0.02 - 0.38	8.36x10-2	1.45x10-1	50.33	1246	314
R_rostralanteriorcingulate	0.22	0.25	0.11	2.06	0.01 - 0.44	3.97x10-2	1.63x10-2	55.64	1249	313

Abbreviations: Beta, correlation beta; CI, confidence interval; I^2, heterogeneity index. All *p*-values are reported, uncorrected. See Supplementary Table 3 for a list of cortical abbreviations. For ease of comparison, Cohen's *d* estimates and *p*-values extracted using 3T data only are provided alongside Cohen's d estimates and *p*-values extracted from the full sample, combining 1.5T and 3T datasets. *P*-values that passed the Bonferroni-adjusted alpha level ($p<1.49x10^{-4}$) are highlighted in bold red (see Methods for statistical threshold determination). Brain regions reported as significant in the full sample, but not in the 3T sample, are highlighted in yellow. Brain regions reported as significant in the 3T sample, but not in the full sample, are highlighted in green.

Supplementary Table 18. Effect sizes for cortical thickness differences (Cohen's *d*) between healthy controls and the 'IGE' phenotype; 3T field strength only.

Structure	Cohen's *d* (3T only)	Cohen's *d* (full sample)	SE	Z score	95% CI	*P* value (3T only)	*P* value (full sample)	I^2	Number of controls	Number of cases
**R_precentral**	**-0.39**	**-0.39**	**0.07**	**-5.35**	**-0.53 - -0.24**	**8.92x10-8**	**5.27x10-8**	**0.56**	**1025**	**294**
**L_precentral**	**-0.34**	**-0.34**	**0.07**	**-4.78**	**-0.48 - -0.2**	**1.77x10-6**	**1.75x10-6**	**0.00**	**1024**	**296**
R_temporalpole	-0.27	-0.28	0.09	-2.95	-0.45 - -0.09	3.13x10-3	2.82x10-3	31.92	1026	295
L_caudalmiddlefrontal	-0.20	-0.20	0.07	-2.77	-0.34 - -0.06	5.60x10-3	5.08x10-3	2.04	1028	296
L_paracentral	-0.18	-0.18	0.07	-2.51	-0.32 - -0.04	1.22x10-2	1.25x10-2	0.00	1028	296
L_entorhinal	-0.18	-0.19	0.08	-2.13	-0.34 - -0.01	3.29x10-2	2.15x10-2	0.00	801	241
R_caudalmiddlefrontal	-0.17	-0.17	0.10	-1.79	-0.36 - 0.02	7.32x10-2	6.66x10-2	37.04	1029	296
L_temporalpole	-0.16	-0.17	0.08	-1.94	-0.33 - 0	5.22x10-2	4.53x10-2	20.49	1022	296
L_supramarginal	-0.14	-0.14	0.08	-1.84	-0.3 - 0.01	6.56x10-2	6.57x10-2	6.16	984	273
R_entorhinal	-0.14	-0.15	0.14	-1.00	-0.41 - 0.14	3.20x10-1	2.54x10-1	58.60	791	239
R_parsorbitalis	-0.12	-0.12	0.07	-1.72	-0.26 - 0.02	8.58x10-2	8.70x10-2	0.00	1030	296
R_superiorparietal	-0.12	-0.13	0.07	-1.72	-0.26 - 0.02	8.62x10-2	5.81x10-2	0.00	1029	296
R_lateralorbitofrontal	-0.12	-0.11	0.07	-1.67	-0.26 - 0.02	9.49x10-2	1.32x10-1	0.00	1027	296
L_middletemporal	-0.12	-0.11	0.07	-1.58	-0.26 - 0.03	1.15x10-1	1.28x10-1	0.00	944	278
R_medialorbitofrontal	-0.11	-0.11	0.07	-1.59	-0.26 - 0.03	1.11x10-1	1.39x10-1	0.00	1012	291
R_paracentral	-0.11	-0.13	0.11	-1.09	-0.32 - 0.09	2.77x10-1	1.86x10-1	47.25	1030	296
L_superiorparietal	-0.11	-0.13	0.09	-1.21	-0.29 - 0.07	2.25x10-1	1.18x10-1	32.65	1026	296
L_inferiorparietal	-0.10	-0.13	0.09	-1.10	-0.29 - 0.08	2.70x10-1	1.52x10-1	35.41	1017	296
L_transversetemporal	-0.10	-0.10	0.07	-1.43	-0.24 - 0.04	1.51x10-1	1.56x10-1	0.00	1025	296
R_superiorfrontal	-0.10	-0.10	0.08	-1.24	-0.26 - 0.06	2.17x10-1	2.27x10-1	19.34	1026	296

**L_fusiform**	-0.10	-0.11	0.09	-1.02	-0.28 - 0.09	3.05x10-1	2.50x10-1	34.87	1025	295
**L_lateraloccipital**	-0.09	-0.13	0.15	-0.60	-0.39 - 0.21	5.51x10-1	4.05x10-1	75.06	1028	295
**L_bankssts**	-0.09	-0.08	0.12	-0.77	-0.32 - 0.14	4.40x10-1	5.03x10-1	53.16	995	271
**R_inferiortemporal**	-0.08	-0.09	0.07	-1.12	-0.22 - 0.06	2.61x10-1	1.84x10-1	0.00	1028	296
**R_precuneus**	-0.07	-0.09	0.09	-0.85	-0.24 - 0.1	3.98x10-1	2.39x10-1	23.58	1030	296
**R_transversetemporal**	-0.07	-0.07	0.10	-0.70	-0.27 - 0.13	4.83x10-1	4.76x10-1	43.96	1030	296
**R_parsopercularis**	-0.07	-0.08	0.07	-1.00	-0.21 - 0.07	3.19x10-1	2.37x10-1	0.00	1028	296
**R_supramarginal**	-0.06	-0.07	0.08	-0.81	-0.21 - 0.09	4.16x10-1	3.51x10-1	0.00	974	269
**R_lingual**	-0.06	-0.06	0.10	-0.58	-0.26 - 0.14	5.63x10-1	5.54x10-1	44.69	1029	295
**R_middletemporal**	-0.06	-0.07	0.07	-0.82	-0.2 - 0.08	4.14x10-1	3.58x10-1	0.00	1023	295
**L_insula**	-0.06	-0.06	0.07	-0.81	-0.2 - 0.08	4.20x10-1	3.78x10-1	0.00	989	292
**L_rostralanteriorcingulate**	-0.06	-0.04	0.11	-0.50	-0.27 - 0.16	6.18x10-1	7.24x10-1	52.44	1023	293
**R_pericalcarine**	-0.05	-0.07	0.12	-0.45	-0.28 - 0.18	6.55x10-1	5.26x10-1	56.02	1030	295
**R_rostralmiddlefrontal**	-0.05	-0.05	0.07	-0.66	-0.2 - 0.1	5.09x10-1	5.35x10-1	6.36	1030	296
**R_insula**	-0.04	-0.03	0.07	-0.52	-0.18 - 0.1	6.04x10-1	6.74x10-1	0.00	991	289
**L_pericalcarine**	-0.04	-0.04	0.10	-0.34	-0.24 - 0.17	7.35x10-1	6.67x10-1	45.91	1028	296
**L_cuneus**	-0.03	-0.09	0.11	-0.30	-0.26 - 0.19	7.65x10-1	3.38x10-1	54.73	1026	296
**L_parsopercularis**	-0.03	-0.02	0.11	-0.28	-0.25 - 0.19	7.77x10-1	8.23x10-1	53.23	1027	296
**R_cuneus**	-0.03	-0.08	0.10	-0.31	-0.23 - 0.17	7.59x10-1	3.25x10-1	43.05	1028	296
**R_rostralanteriorcingulate**	-0.03	-0.03	0.08	-0.37	-0.19 - 0.13	7.09x10-1	7.47x10-1	16.52	1028	296
**L_parstriangularis**	-0.02	-0.01	0.08	-0.30	-0.17 - 0.12	7.63x10-1	8.51x10-1	7.20	1028	296
**L_inferiortemporal**	-0.02	-0.04	0.08	-0.27	-0.19 - 0.14	7.90x10-1	6.45x10-1	20.94	1027	295
**R_parstriangularis**	-0.02	-0.02	0.07	-0.29	-0.16 - 0.12	7.69x10-1	7.63x10-1	0.01	1028	296
**L_precuneus**	-0.02	-0.04	0.09	-0.24	-0.19 - 0.15	8.14x10-1	6.66x10-1	23.86	1027	296
**L_frontalpole**	-0.01	-0.02	0.09	-0.17	-0.18 - 0.16	8.65x10-1	8.40x10-1	25.46	1026	296
**L_superiortemporal**	-0.01	-0.03	0.10	-0.14	-0.21 - 0.19	8.92x10-1	7.99x10-1	37.82	925	265
**L_superiorfrontal**	-0.01	-0.01	0.07	-0.12	-0.15 - 0.14	9.05x10-1	8.53x10-1	5.11	1027	296

**R_inferiorparietal**	-0.01	-0.03	0.09	-0.07	-0.18 - 0.17	9.47x10-1	7.61x10-1	29.81	1019	292
**L_medialorbitofrontal**	0.00	0.00	0.07	-0.01	-0.14 - 0.14	9.89x10-1	9.84x10-1	0.00	1010	292
**L_lingual**	0.00	0.00	0.10	0.03	-0.19 - 0.2	9.75x10-1	9.72x10-1	40.45	1027	296
**R_parahippocampal**	0.00	0.00	0.07	0.05	-0.14 - 0.14	9.61x10-1	9.89x10-1	0.01	1026	292
**L_parsorbitalis**	0.00	0.00	0.07	0.07	-0.14 - 0.14	9.48x10-1	9.77x10-1	0.00	1028	295
**L_isthmuscingulate**	0.01	0.00	0.11	0.05	-0.21 - 0.22	9.63x10-1	9.68x10-1	50.14	1027	295
**R_lateraloccipital**	0.02	-0.05	0.16	0.10	-0.29 - 0.32	9.20x10-1	7.23x10-1	76.38	1029	296
**R_isthmuscingulate**	0.02	0.02	0.10	0.19	-0.18 - 0.22	8.46x10-1	8.37x10-1	44.08	1027	296
**L_rostralmiddlefrontal**	0.02	0.02	0.09	0.26	-0.15 - 0.19	7.91x10-1	8.30x10-1	25.03	1027	294
**L_posteriorcingulate**	0.03	0.03	0.09	0.34	-0.14 - 0.2	7.37x10-1	7.23x10-1	28.08	1028	296
**R_caudalanteriorcingulate**	0.03	0.05	0.08	0.41	-0.13 - 0.19	6.84x10-1	5.44x10-1	18.84	1028	296
**R_frontalpole**	0.04	0.04	0.07	0.51	-0.1 - 0.18	6.10x10-1	5.82x10-1	0.00	1030	296
**L_parahippocampal**	0.04	0.04	0.09	0.44	-0.14 - 0.22	6.61x10-1	6.60x10-1	30.98	1019	293
**R_fusiform**	0.04	0.03	0.10	0.40	-0.16 - 0.24	6.88x10-1	7.96x10-1	43.53	1030	295
**R_superiortemporal**	0.06	0.04	0.10	0.55	-0.14 - 0.26	5.81x10-1	6.71x10-1	36.70	936	264
**L_postcentral**	0.06	0.05	0.07	0.89	-0.08 - 0.2	3.74x10-1	4.43x10-1	0.00	1025	295
**R_postcentral**	0.07	0.06	0.09	0.76	-0.11 - 0.24	4.49x10-1	5.07x10-1	28.63	1024	296
**R_posteriorcingulate**	0.07	0.08	0.11	0.66	-0.14 - 0.29	5.11x10-1	4.75x10-1	51.80	1030	295
**L_caudalanteriorcingulate**	0.08	0.09	0.08	0.93	-0.09 - 0.24	3.53x10-1	3.02x10-1	20.35	1023	295
**L_lateralorbitofrontal**	0.09	0.09	0.07	1.24	-0.05 - 0.23	2.17x10-1	1.94x10-1	0.00	1023	296
**R_bankssts**	0.12	0.10	0.12	0.95	-0.12 - 0.36	3.40x10-1	4.10x10-1	60.11	1010	286

Abbreviations: Beta, correlation beta; CI, confidence interval; I^2, heterogeneity index. All *p*-values are reported, uncorrected. See Supplementary Table 3 for a list of cortical abbreviations. For ease of comparison, Cohen's *d* estimates and *p*-values extracted using 3T data only are provided alongside Cohen's d estimates and *p*-values extracted from the full sample, combining 1.5T and 3T datasets. *P*-values that passed the Bonferroni-adjusted alpha level ($p<1.49x10^{-4}$) are highlighted in bold red (see Methods for statistical threshold determination).

Supplementary Table 19. Effect sizes for cortical thickness differences (Cohen's *d*) between healthy controls and the 'all-other-epilepsies' phenotype; 3T field strength only.

Structure	Cohen's *d* (3T only)	Cohen's *d* (full sample)	SE	Z score	95% CI	*P* value (3T only)	*P* value (full sample)	I^2	Number of controls	Number of cases
L_precentral	-0.375	-0.375	0.046	-8.113	-0.465 - -0.284	4.94x10-16	1.76x10-16	0.658	1423	985
R_precentral	-0.346	-0.348	0.046	-7.554	-0.436 - -0.256	4.20x10-14	1.70x10-14	0.000	1429	984
R_paracentral	-0.345	-0.351	0.047	-7.294	-0.438 - -0.252	3.00x10-13	1.05x10-14	4.439	1432	986
L_caudalmiddlefrontal	-0.286	-0.291	0.046	-6.255	-0.376 - -0.196	3.98x10-10	1.32x10-10	0.000	1428	988
L_paracentral	-0.256	-0.257	0.046	-5.608	-0.346 - -0.167	2.05x10-8	1.34x10-08	0.000	1428	988
L_superiorfrontal	-0.242	-0.243	0.059	-4.079	-0.358 - -0.126	4.51x10-5	3.51x10-05	34.457	1427	987
R_superiorfrontal	-0.239	-0.235	0.054	-4.432	-0.345 - -0.133	9.32x10-6	7.15x10-06	22.626	1429	985
R_cuneus	-0.237	-0.234	0.046	-5.190	-0.326 - -0.147	2.10x10-7	2.15x10-07	0.000	1430	984
R_precuneus	-0.232	-0.238	0.057	-4.109	-0.343 - -0.122	3.97x10-5	7.78x10-06	28.591	1432	982
L_superiorparietal	-0.222	-0.224	0.046	-4.856	-0.312 - -0.132	1.20x10-6	7.27x10-07	0.004	1425	984
R_superiorparietal	-0.217	-0.22	0.046	-4.682	-0.308 - -0.126	2.85x10-6	1.15x10-06	2.322	1431	985
R_parstriangularis	-0.214	-0.21	0.046	-4.697	-0.304 - -0.125	2.64x10-6	3.32x10-06	0.002	1430	986
R_caudalmiddlefrontal	-0.212	-0.212	0.046	-4.653	-0.302 - -0.123	3.27x10-6	2.62x10-06	0.000	1432	986
R_rostralmiddlefrontal	-0.206	-0.198	0.054	-3.826	-0.311 - -0.1	1.30x10-4	1.58x10-04	22.124	1432	985
R_supramarginal	-0.204	-0.206	0.047	-4.324	-0.296 - -0.111	1.53x10-5	9.95x10-06	0.000	1376	949
R_lateraloccipital	-0.198	-0.211	0.046	-4.339	-0.288 - -0.109	1.43x10-5	3.18x10-06	0.003	1431	985
L_transversetemporal	-0.196	-0.2	0.057	-3.409	-0.308 - -0.083	6.53x10-4	6.62x10-04	30.497	1425	988
R_lateralorbitofrontal	-0.181	-0.167	0.057	-3.166	-0.293 - -0.069	1.54x10-3	2.15x10-03	29.917	1429	984
R_entorhinal	-0.180	-0.197	0.053	-3.422	-0.284 - -0.077	6.22x10-4	7.30x10-04	8.046	1164	889
L_precuneus	-0.172	-0.178	0.050	-3.416	-0.271 - -0.073	6.36x10-4	1.34x10-04	13.612	1427	986
R_lingual	-0.171	-0.18	0.046	-3.746	-0.26 - -0.081	1.80x10-4	7.12x10-05	0.001	1431	984
R_transversetemporal	-0.169	-0.18	0.046	-3.712	-0.259 - -0.08	2.06x10-4	6.84x10-05	0.002	1432	986
R_parsorbitalis	-0.161	-0.156	0.053	-3.036	-0.265 - -0.057	2.40x10-3	2.69x10-03	20.495	1432	984
L_pericalcarine	-0.160	-0.16	0.055	-2.902	-0.268 - -0.052	3.70x10-3	3.10x10-03	25.577	1428	987
R_pericalcarine	-0.158	-0.158	0.049	-3.210	-0.255 - -0.062	1.33x10-3	9.68x10-04	10.765	1432	985
L_parstriangularis	-0.146	-0.141	0.046	-3.213	-0.236 - -0.057	1.31x10-3	2.00x10-3	0.002	1428	988

**L_entorhinal**	-0.142	-0.149	0.083	-1.718	-0.304 - 0.02	8.58x10-2	7.70x10-2	59.503	1191	901
**L_lateraloccipital**	-0.137	-0.153	0.055	-2.508	-0.244 - -0.03	1.21x10-2	1.39x10-03	24.445	1428	988
**L_supramarginal**	-0.134	-0.138	0.059	-2.279	-0.25 - -0.019	2.27x10-2	1.90x10-2	30.535	1384	949
**L_parsorbitalis**	-0.131	-0.128	0.046	-2.869	-0.22 - -0.041	4.12x10-3	5.00x10-3	0.000	1428	987
**L_parsopercularis**	-0.123	-0.122	0.048	-2.535	-0.218 - -0.028	1.13x10-2	1.00x10-2	8.606	1426	987
**L_cuneus**	-0.121	-0.142	0.061	-1.995	-0.24 - -0.002	4.60x10-2	4.00x10-3	37.263	1426	988
**R_parsopercularis**	-0.119	-0.125	0.047	-2.514	-0.212 - -0.026	1.19x10-2	8.00x10-3	5.521	1431	986
**R_temporalpole**	-0.119	-0.126	0.047	-2.557	-0.21 - -0.028	1.06x10-2	7.00x10-3	2.738	1429	983
**R_medialorbitofrontal**	-0.113	-0.101	0.056	-1.994	-0.223 - -0.002	4.61x10-2	6.50x10-2	27.535	1414	961
**R_inferiorparietal**	-0.110	-0.118	0.053	-2.100	-0.213 - -0.007	3.58x10-2	1.80x10-2	18.803	1421	977
**R_superiortemporal**	-0.107	-0.11	0.058	-1.823	-0.221 - 0.008	6.83x10-2	5.90x10-2	29.393	1335	947
**R_frontalpole**	-0.097	-0.098	0.046	-2.135	-0.187 - -0.008	3.28x10-2	3.00x10-2	0.004	1432	985
**L_rostralmiddlefrontal**	-0.095	-0.097	0.062	-1.532	-0.216 - 0.026	1.26x10-1	1.16x10-1	39.618	1428	987
**L_lingual**	-0.094	-0.099	0.045	-2.065	-0.183 - -0.005	3.90x10-2	2.80x10-2	0.006	1427	988
**R_middletemporal**	-0.093	-0.102	0.046	-2.041	-0.183 - -0.004	4.13x10-2	2.40x10-2	0.000	1421	981
**L_posteriorcingulate**	-0.091	-0.089	0.062	-1.476	-0.212 - 0.03	1.40x10-1	1.45x10-1	39.249	1428	987
**L_isthmuscingulate**	-0.091	-0.093	0.057	-1.576	-0.203 - 0.022	1.15x10-1	1.03x10-1	30.825	1427	986
**L_fusiform**	-0.088	-0.095	0.046	-1.933	-0.178 - 0.001	5.33x10-2	3.50x10-2	0.001	1426	987
**L_inferiorparietal**	-0.087	-0.096	0.046	-1.899	-0.177 - 0.003	5.76x10-2	3.40x10-2	0.017	1417	983
**L_superiortemporal**	-0.082	-0.092	0.058	-1.414	-0.197 - 0.032	1.57x10-1	1.23x10-1	28.471	1311	940
**L_temporalpole**	-0.081	-0.088	0.068	-1.191	-0.214 - 0.052	2.34x10-1	2.00x10-1	48.995	1423	985
**L_frontalpole**	-0.080	-0.089	0.046	-1.761	-0.169 - 0.009	7.83x10-2	5.10x10-2	0.000	1426	987
**R_parahippocampal**	-0.079	-0.076	0.046	-1.724	-0.168 - 0.011	8.47x10-2	9.30x10-2	0.000	1428	984
**R_bankssts**	-0.076	-0.081	0.064	-1.184	-0.201 - 0.05	2.36x10-1	2.01x10-1	42.001	1402	971
**R_fusiform**	-0.072	-0.086	0.062	-1.162	-0.193 - 0.049	2.45x10-1	1.64x10-1	39.391	1432	985
**L_lateralorbitofrontal**	-0.071	-0.065	0.066	-1.073	-0.201 - 0.059	2.83x10-1	3.15x10-1	47.047	1424	987
**R_insula**	-0.068	-0.069	0.046	-1.477	-0.159 - 0.022	1.40x10-1	1.29x10-1	0.001	1393	978
**L_middletemporal**	-0.066	-0.074	0.047	-1.410	-0.158 - 0.026	1.58x10-1	1.12x10-1	0.000	1326	946
**L_postcentral**	-0.066	-0.065	0.046	-1.437	-0.155 - 0.024	1.51x10-1	1.52x10-1	0.009	1425	985
**R_posteriorcingulate**	-0.065	-0.061	0.062	-1.050	-0.186 - 0.056	2.94x10-1	3.24x10-1	39.657	1432	986
**L_parahippocampal**	-0.063	-0.06	0.046	-1.373	-0.152 - 0.027	1.70x10-1	1.84x10-1	0.000	1419	983
**L_rostralanteriorcingulate**	-0.026	-0.023	0.069	-0.379	-0.161 - 0.109	7.05x10-1	7.31x10-1	50.462	1423	982

**R_postcentral**	-0.025	-0.024	0.046	-0.554	-0.114 - 0.064	5.80x10-1	5.97x10-1	0.003	1428	984
**R_inferiortemporal**	-0.015	-0.035	0.045	-0.335	-0.104 - 0.074	7.38x10-1	4.83x10-1	0.010	1430	984
**L_insula**	-0.014	-0.015	0.053	-0.260	-0.118 - 0.09	7.95x10-1	7.77x10-1	19.334	1390	973
**R_isthmuscingulate**	-0.013	-0.016	0.056	-0.238	-0.123 - 0.096	8.12x10-1	7.74x10-1	27.675	1429	985
**L_medialorbitofrontal**	-0.011	-0.014	0.046	-0.229	-0.101 - 0.08	8.19x10-1	7.54x10-1	0.000	1410	963
**L_bankssts**	0.002	-0.007	0.063	0.028	-0.122 - 0.126	9.77x10-1	9.18x10-1	37.993	1362	925
**R_caudalanteriorcingulate**	0.005	0.007	0.049	0.112	-0.091 - 0.102	9.11x10-1	8.88x10-1	10.586	1430	985
**L_inferiortemporal**	0.008	-0.007	0.049	0.153	-0.089 - 0.104	8.79x10-1	9.03x10-1	11.283	1427	984
**R_rostralanteriorcingulate**	0.029	0.023	0.067	0.431	-0.103 - 0.161	6.66x10-1	7.34x10-1	48.447	1431	984
**L_caudalanteriorcingulate**	0.061	0.05	0.053	1.157	-0.042 - 0.165	2.47x10-1	3.36x10-1	20.009	1423	975

Abbreviations: Beta, correlation beta; CI, confidence interval; I^2, heterogeneity index. All *p*-values are reported, uncorrected. See Supplementary Table 3 for a list of cortical abbreviations. For ease of comparison, Cohen's *d* estimates and *p*-values extracted using 3T data only are provided alongside Cohen's d estimates and *p*-values extracted from the full sample, combining 1.5T and 3T datasets. *P*-values that passed the Bonferroni-adjusted alpha level ($p<1.49x10^{-4}$) are highlighted in bold red (see Methods for statistical threshold determination).

Supplementary Table 20. Average ages at onset of epilepsy per research centre.

Research centre	All-epilepsies	IGE	MTLE-L	MTLE-R
**BERN**	-	-	-	-
**BONN**	16.86	-	16.35	17.84
**BRI**	17.9	1.5	10.77	15.7
**BRUSSELS**	14.45	16.38	12.6	-
**CUBRIC**	13.56	12.9	-	-
**EPICZ**	19	-	19.49	17.11
**EPIGEN**	17	-	19.43	25.7
**EPIGEN_1.5T**	14.7	-	15.1	14.2
**FLORENCE**	12.7	10.4	25	-
**GER_HGW**	28.1	28.1	-	-
**IDIBAPS**	18.1	-	15.4	18.9
**KCL_CNS**	13.2	12.1	20.8	-
**KCL_CRF**	23.1	23.3	-	-
**KUOPIO**	25	17.19	-	23.33
**MNI**	16.5	-	18.3	16.1
**NYU**	16.96	14.2	12	15.6
**RMH**	28.2	32.3	25.8	29.2
**EKUT_A**	17	14.8	26.8	-
**EKUT_B**	17.3	15.9	-	-
**UCSD**	19.31	-	15.69	13.14
**UNAM**	16.25	-	18.5	12.5
**UNICAMP**	12.1	12.82	9.93	13.25
**UNIMORE**	12.57	11.36	-	-
**XMU**	-	18.55	16.12	19.07
**Weighted average**	**17.63**	**8.4**	**15.36**	**16.6**

Supplementary Table 21. Full meta-analytical results of the age-by-diagnosis interaction test, controlling for sex and ICV | 'All epilepsies' phenotype

Structure	Beta	SE	Z value	*P* value
Volume measures – subcortical regions, hippocampi, and lateral ventricles				
Accumbens (LH)	-0.12	0.59	-0.20	0.84
Amygala (LH)	0.19	1.21	0.16	0.88
Caudate (LH)	-4.22	1.58	-2.67	0.01
Hippocampus (LH)	-1.10	2.12	-0.52	0.60
Left lateral ventricle	23.30	18.61	1.25	0.21
Pallidum (LH)	-0.46	0.93	-0.50	0.62
Putamen (LH)	-1.93	2.75	-0.70	0.48
Thalamus (LH)	-3.49	3.39	-1.03	0.30
Accumbens (RH)	-0.36	0.50	-0.72	0.47
Amygdala (RH)	-0.06	0.97	-0.06	0.95
Caudate (RH)	-3.40	1.68	-2.03	0.04
Hippocampus (RH)	-4.96	2.30	-2.16	0.03
Right lateral ventricle	35.76	18.13	1.97	0.05
Pallidum (RH)	-0.60	0.76	-0.79	0.43
Putamen (RH)	-4.07	2.97	-1.37	0.17
Thalamus (RH)	-1.56	4.25	-0.37	0.71
Thickness measures – cortical regions				
L_bankssts	-0.0007	0.0006	-1.0670	0.2860
L_caudalanteriorcingulate	0.0004	0.0009	0.4353	0.6633
L_caudalmiddlefrontal	0.0000	0.0007	-0.0541	0.9568
L_cuneus	-0.0012	0.0008	-1.4519	0.1465
L_entorhinal	-0.0009	0.0014	-0.6202	0.5351
L_frontalpole	-0.0010	0.0011	-0.8380	0.4020
L_fusiform	-0.0004	0.0005	-0.8112	0.4173
L_inferiorparietal	-0.0010	0.0006	-1.5997	0.1097
L_inferiortemporal	-0.0005	0.0006	-0.8015	0.4228
L_insula	0.0006	0.0005	1.0968	0.2727

L_isthmuscingulate	-0.0009	0.0009	-1.0466	0.2953
L_lateraloccipital	-0.0009	0.0005	-1.8602	0.0629
L_lateralorbitofrontal	-0.0005	0.0009	-0.6283	0.5298
L_lingual	-0.0008	0.0004	-1.8816	0.0599
L_medialorbitofrontal	-0.0006	0.0006	-1.0843	0.2782
L_middletemporal	-0.0007	0.0006	-1.1416	0.2536
L_paracentral	-0.0006	0.0006	-1.0058	0.3145
L_parahippocampal	-0.0008	0.0013	-0.5939	0.5526
L_parsopercularis	-0.0005	0.0007	-0.7387	0.4601
L_parsorbitalis	-0.0009	0.0008	-1.1287	0.2590
L_parstriangularis	-0.0002	0.0008	-0.2078	0.8354
L_pericalcarine	-0.0004	0.0005	-0.6798	0.4966
L_postcentral	-0.0004	0.0004	-0.9053	0.3653
L_posteriorcingulate	-0.0007	0.0006	-1.1893	0.2343
L_precentral	-0.0008	0.0005	-1.5999	0.1096
L_precuneus	-0.0010	0.0007	-1.4655	0.1428
L_rostralanteriorcingulate	0.0002	0.0012	0.1559	0.8761
L_rostralmiddlefrontal	-0.0006	0.0008	-0.7028	0.4822
L_superiorfrontal	-0.0011	0.0009	-1.3072	0.1911
L_superiorparietal	-0.0007	0.0005	-1.5084	0.1315
L_superiortemporal	-0.0017	0.0006	-2.8763	0.0040
L_supramarginal	-0.0008	0.0005	-1.4898	0.1363
L_temporalpole	-0.0022	0.0012	-1.9136	0.0557
L_transversetemporal	-0.0009	0.0007	-1.2276	0.2196
R_bankssts	-0.0012	0.0008	-1.4480	0.1476
R_caudalanteriorcingulate	-0.0005	0.0012	-0.3845	0.7006
R_caudalmiddlefrontal	-0.0001	0.0007	-0.0919	0.9268
R_cuneus	-0.0016	0.0009	-1.8425	0.0654
R_entorhinal	-0.0003	0.0016	-0.2011	0.8406
R_frontalpole	0.0003	0.0012	0.2506	0.8021
R_fusiform	-0.0014	0.0005	-2.5991	0.0093
R_inferiorparietal	-0.0013	0.0006	-2.2270	0.0259

R_inferiortemporal	-0.0010	0.0006	-1.6811	0.0927
R_insula	-0.0003	0.0006	-0.5816	0.5608
R_isthmuscingulate	-0.0010	0.0009	-1.0593	0.2895
R_lateraloccipital	-0.0015	0.0005	-2.9762	0.0029
R_lateralorbitofrontal	-0.0005	0.0009	-0.5337	0.5935
R_lingual	-0.0012	0.0007	-1.7948	0.0727
R_medialorbitofrontal	-0.0002	0.0006	-0.2774	0.7815
R_middletemporal	-0.0013	0.0008	-1.7078	0.0877
R_paracentral	0.0003	0.0007	0.3688	0.7122
R_parahippocampal	-0.0008	0.0010	-0.8312	0.4059
R_parsopercularis	-0.0004	0.0008	-0.4805	0.6309
R_parsorbitalis	0.0008	0.0009	0.8364	0.4029
R_parstriangularis	0.0000	0.0007	0.0442	0.9647
R_pericalcarine	-0.0013	0.0005	-2.6732	0.0075
R_postcentral	-0.0005	0.0005	-1.0434	0.2968
R_posteriorcingulate	0.0001	0.0005	0.2492	0.8032
R_precentral	-0.0005	0.0005	-1.1036	0.2698
R_precuneus	-0.0012	0.0008	-1.4186	0.1560
R_rostralanteriorcingulate	-0.0013	0.0008	-1.6635	0.0962
R_rostralmiddlefrontal	-0.0001	0.0007	-0.1245	0.9009
R_superiorfrontal	-0.0001	0.0008	-0.1708	0.8643
R_superiorparietal	-0.0009	0.0005	-1.8607	0.0628
R_superiortemporal	-0.0018	0.0006	-3.1373	0.0017
R_supramarginal	-0.0009	0.0005	-1.6960	0.0899
R_temporalpole	0.0006	0.0016	0.4052	0.6853
R_transversetemporal	-0.0003	0.0008	-0.4334	0.6648

Abbreviations: Beta, correlation beta; LH, left hemisphere; RH, right hemisphere. See Supplementary Table 3 for a list of cortical abbreviations. All uncorrected p-values are reported.

Supplementary Table 22. Full meta-analytical results of the age-by-diagnosis interaction test, controlling for sex and ICV | 'MTLE-L' phenotype

Structure	Beta	SE	Z value	*P* value
Volume measures – subcortical regions, hippocampi, and lateral ventricles				
Accumbens (LH)	-0.003	0.200	-0.014	0.989
Amygala (LH)	-0.473	0.411	-1.152	0.249
Caudate (LH)	-0.002	0.548	-0.004	0.996
Hippocampus (LH)	0.181	0.678	0.267	0.790
Left lateral ventricle	-3.918	5.238	-0.748	0.454
Pallidum (LH)	0.065	0.468	0.138	0.890
Putamen (LH)	0.053	1.236	0.043	0.966
Thalamus (LH)	0.227	1.640	0.138	0.890
Accumbens (RH)	0.101	0.282	0.358	0.721
Amygdala (RH)	-0.981	0.462	-2.122	0.034
Caudate (RH)	-0.007	0.582	-0.012	0.991
Hippocampus (RH)	-3.279	1.015	-3.231	0.001
Right lateral ventricle	1.160	6.230	0.186	0.852
Pallidum (RH)	0.146	0.340	0.431	0.667
Putamen (RH)	-1.057	1.338	-0.790	0.429
Thalamus (RH)	-0.857	1.470	-0.583	0.560
Thickness measures – cortical regions				
L_bankssts	0.0003	0.0003	0.8667	0.3861
L_caudalanteriorcingulate	0.0001	0.0004	0.3317	0.7401
L_caudalmiddlefrontal	0.0002	0.0003	0.5181	0.6044
L_cuneus	-0.0002	0.0002	-0.7885	0.4304
L_entorhinal	-0.0001	0.0006	-0.1163	0.9075
L_frontalpole	-0.0003	0.0004	-0.6369	0.5242
L_fusiform	-0.0002	0.0002	-0.8211	0.4116
L_inferiorparietal	-0.0002	0.0003	-0.6815	0.4955
L_inferiortemporal	-0.0004	0.0003	-1.4295	0.1529
L_insula	0.0003	0.0003	0.9102	0.3627

L_isthmuscingulate	-0.0005	0.0003	-1.6757	0.0938
L_lateraloccipital	-0.0002	0.0002	-0.9501	0.3421
L_lateralorbitofrontal	0.0000	0.0003	0.1875	0.8513
L_lingual	-0.0003	0.0002	-1.2107	0.2260
L_medialorbitofrontal	-0.0001	0.0003	-0.2065	0.8364
L_middletemporal	-0.0001	0.0003	-0.2166	0.8286
L_paracentral	-0.0001	0.0003	-0.2218	0.8245
L_parahippocampal	0.0005	0.0005	0.9967	0.3189
L_parsopercularis	-0.0001	0.0002	-0.2841	0.7764
L_parsorbitalis	0.0002	0.0004	0.4582	0.6468
L_parstriangularis	0.0000	0.0003	0.0287	0.9771
L_pericalcarine	-0.0001	0.0003	-0.2011	0.8406
L_postcentral	-0.0002	0.0002	-1.0703	0.2845
L_posteriorcingulate	-0.0001	0.0005	-0.2835	0.7768
L_precentral	-0.0001	0.0002	-0.5226	0.6013
L_precuneus	0.0000	0.0003	-0.1068	0.9150
L_rostralanteriorcingulate	0.0006	0.0004	1.3229	0.1859
L_rostralmiddlefrontal	0.0000	0.0002	-0.0316	0.9748
L_superiorfrontal	-0.0002	0.0002	-1.0477	0.2948
L_superiorparietal	-0.0001	0.0002	-0.7245	0.4687
L_superiortemporal	-0.0005	0.0003	-1.9950	0.0460
L_supramarginal	-0.0002	0.0002	-1.0924	0.2747
L_temporalpole	-0.0009	0.0006	-1.5518	0.1207
L_transversetemporal	-0.0005	0.0003	-1.4167	0.1566
R_bankssts	0.0000	0.0004	-0.0835	0.9335
R_caudalanteriorcingulate	0.0000	0.0004	-0.0706	0.9437
R_caudalmiddlefrontal	-0.0001	0.0002	-0.5055	0.6132
R_cuneus	-0.0001	0.0002	-0.6384	0.5232
R_entorhinal	-0.0010	0.0006	-1.6935	0.0904
R_frontalpole	-0.0004	0.0005	-0.9201	0.3575
R_fusiform	-0.0005	0.0003	-1.4755	0.1401

R_inferiorparietal	-0.0002	0.0003	-0.7756	0.4380
R_inferiortemporal	-0.0004	0.0003	-1.4009	0.1613
R_insula	-0.0001	0.0003	-0.5158	0.6060
R_isthmuscingulate	0.0000	0.0003	0.0478	0.9618
R_lateraloccipital	-0.0005	0.0002	-2.1323	0.0330
R_lateralorbitofrontal	0.0001	0.0002	0.2913	0.7708
R_lingual	-0.0004	0.0002	-1.6963	0.0898
R_medialorbitofrontal	0.0000	0.0003	0.0407	0.9676
R_middletemporal	-0.0002	0.0003	-0.6998	0.4841
R_paracentral	0.0003	0.0002	1.0670	0.2860
R_parahippocampal	0.0000	0.0004	-0.0809	0.9355
R_parsopercularis	-0.0001	0.0002	-0.5454	0.5855
R_parsorbitalis	0.0007	0.0003	2.0445	0.0409
R_parstriangularis	0.0002	0.0002	0.7253	0.4683
R_pericalcarine	0.0001	0.0003	0.2538	0.7996
R_postcentral	0.0000	0.0002	-0.1008	0.9197
R_posteriorcingulate	0.0000	0.0002	0.0283	0.9774
R_precentral	0.0000	0.0002	0.0013	0.9989
R_precuneus	-0.0001	0.0002	-0.2282	0.8195
R_rostralanteriorcingulate	-0.0004	0.0004	-0.9708	0.3317
R_rostralmiddlefrontal	0.0001	0.0002	0.2877	0.7736
R_superiorfrontal	-0.0001	0.0002	-0.4660	0.6412
R_superiorparietal	-0.0002	0.0002	-0.9406	0.3469
R_superiortemporal	-0.0004	0.0003	-1.7037	0.0884
R_supramarginal	-0.0002	0.0002	-1.0248	0.3055
R_temporalpole	-0.0001	0.0006	-0.2610	0.7941
R_transversetemporal	-0.0001	0.0004	-0.2853	0.7754

Abbreviations: Beta, correlation beta; LH, left hemisphere; RH, right hemisphere. See Supplementary Table 3 for a list of cortical abbreviations. All uncorrected p-values are reported.

Supplementary Table 23. Full meta-analytical results of the age-by-diagnosis interaction test, controlling for sex and ICV | 'MTLE-R' phenotype

**Structure**	**Beta**	**SE**	**Z value**	***P* value**
Volume measures – subcortical regions, hippocampi, and lateral ventricles				
Accumbens (LH)	-0.046	0.0003	0.7633	0.828
Amygala (LH)	-0.380	0.0004	0.6991	0.367
Caudate (LH)	-0.249	0.0003	-0.0197	0.672
Hippocampus (LH)	0.003	0.0002	-0.9810	0.997
Left lateral ventricle	-4.437	0.0006	0.3237	0.405
Pallidum (LH)	-0.368	0.0004	-0.6924	0.416
Putamen (LH)	-0.312	0.0002	-0.8290	0.808
Thalamus (LH)	0.659	0.0003	-0.5496	0.675
Accumbens (RH)	0.079	0.0003	-1.3410	0.794
Amygdala (RH)	-0.864	0.0003	0.6052	0.050
Caudate (RH)	-0.316	0.0003	-1.4816	0.607
Hippocampus (RH)	-3.832	0.0003	-1.3226	0.001
Right lateral ventricle	1.200	0.0002	0.5318	0.856
Pallidum (RH)	0.028	0.0002	-1.7378	0.940
Putamen (RH)	-0.651	0.0003	-0.5819	0.649
Thalamus (RH)	-1.180	0.0003	0.0761	0.438
Thickness measures – cortical regions				
L_bankssts	0.0002	0.0003	0.7633	0.4453
L_caudalanteriorcingulate	0.0003	0.0004	0.6991	0.4845
L_caudalmiddlefrontal	0.0000	0.0003	-0.0197	0.9843
L_cuneus	-0.0002	0.0002	-0.9810	0.3266
L_entorhinal	0.0002	0.0006	0.3237	0.7462
L_frontalpole	-0.0003	0.0004	-0.6924	0.4887
L_fusiform	-0.0002	0.0002	-0.8290	0.4071
L_inferiorparietal	-0.0002	0.0003	-0.5496	0.5826
L_inferiortemporal	-0.0003	0.0003	-1.3410	0.1799

L_insula	0.0002	0.0003	0.6052	0.5450
L_isthmuscingulate	-0.0004	0.0003	-1.4816	0.1384
L_lateraloccipital	-0.0003	0.0003	-1.3226	0.1860
L_lateralorbitofrontal	0.0001	0.0002	0.5318	0.5949
L_lingual	-0.0004	0.0002	-1.7378	0.0822
L_medialorbitofrontal	-0.0002	0.0003	-0.5819	0.5607
L_middletemporal	0.0000	0.0003	0.0761	0.9393
L_paracentral	-0.0004	0.0003	-1.2808	0.2003
L_parahippocampal	0.0000	0.0006	-0.0492	0.9608
L_parsopercularis	0.0000	0.0002	0.1866	0.8519
L_parsorbitalis	0.0002	0.0003	0.5061	0.6128
L_parstriangularis	0.0000	0.0002	-0.0660	0.9474
L_pericalcarine	-0.0001	0.0003	-0.4468	0.6550
L_postcentral	-0.0003	0.0002	-1.5272	0.1267
L_posteriorcingulate	-0.0002	0.0003	-0.7550	0.4502
L_precentral	-0.0003	0.0002	-1.2943	0.1956
L_precuneus	-0.0001	0.0003	-0.4288	0.6680
L_rostralanteriorcingulate	0.0003	0.0003	0.8872	0.3750
L_rostralmiddlefrontal	0.0000	0.0002	0.1929	0.8470
L_superiorfrontal	-0.0003	0.0002	-1.1611	0.2456
L_superiorparietal	-0.0002	0.0003	-0.9564	0.3389
L_superiortemporal	-0.0004	0.0002	-1.6722	0.0945
L_supramarginal	-0.0003	0.0002	-1.5041	0.1326
L_temporalpole	-0.0005	0.0005	-1.0227	0.3064
L_transversetemporal	-0.0007	0.0003	-2.0131	0.0441
R_bankssts	0.0001	0.0005	0.1137	0.9095
R_caudalanteriorcingulate	0.0002	0.0003	0.4753	0.6345
R_caudalmiddlefrontal	-0.0002	0.0002	-0.6924	0.4887
R_cuneus	-0.0003	0.0002	-1.3833	0.1666
R_entorhinal	-0.0005	0.0006	-0.8789	0.3795
R_frontalpole	-0.0003	0.0005	-0.6569	0.5112

R_fusiform	-0.0003	0.0002	-1.4418	0.1494
R_inferiorparietal	-0.0002	0.0003	-0.8202	0.4121
R_inferiortemporal	-0.0004	0.0003	-1.1445	0.2524
R_insula	-0.0001	0.0002	-0.3243	0.7457
R_isthmuscingulate	0.0000	0.0003	-0.1398	0.8888
R_lateraloccipital	-0.0005	0.0002	-2.5310	0.0114
R_lateralorbitofrontal	0.0003	0.0002	1.0486	0.2943
R_lingual	-0.0004	0.0002	-1.7020	0.0887
R_medialorbitofrontal	0.0000	0.0003	0.0192	0.9847
R_middletemporal	-0.0002	0.0003	-0.6148	0.5387
R_paracentral	0.0001	0.0002	0.4298	0.6673
R_parahippocampal	-0.0002	0.0004	-0.3614	0.7178
R_parsopercularis	-0.0002	0.0002	-0.7761	0.4377
R_parsorbitalis	0.0008	0.0003	2.2921	0.0219
R_parstriangularis	0.0002	0.0002	0.9915	0.3214
R_pericalcarine	-0.0002	0.0003	-0.8538	0.3932
R_postcentral	-0.0002	0.0002	-0.9812	0.3265
R_posteriorcingulate	0.0001	0.0002	0.2580	0.7964
R_precentral	-0.0002	0.0002	-0.7935	0.4275
R_precuneus	-0.0002	0.0002	-0.6426	0.5205
R_rostralanteriorcingulate	-0.0002	0.0004	-0.4953	0.6204
R_rostralmiddlefrontal	0.0001	0.0002	0.3287	0.7424
R_superiorfrontal	-0.0002	0.0002	-0.7724	0.4399
R_superiorparietal	-0.0003	0.0002	-1.3505	0.1769
R_superiortemporal	-0.0005	0.0002	-1.9055	0.0567
R_supramarginal	-0.0004	0.0002	-1.7222	0.0850
R_temporalpole	-0.0003	0.0005	-0.5144	0.6070
R_transversetemporal	-0.0001	0.0003	-0.3257	0.7446

Abbreviations: Beta, correlation beta; SE, standard error; LH, left hemisphere; RH, right hemisphere. See Supplementary Table 3 for a list of cortical abbreviations. All uncorrected p-values are reported.

Supplementary Table 24. Full meta-analytical results of the age-by-diagnosis interaction test, controlling for sex and ICV | 'IGE' phenotype

**Structure**	**Beta**	**SE**	**Z value**	***P* value**
Volume measures – subcortical regions, hippocampi, and lateral ventricles				
Accumbens (LH)	0.048	0.200	0.241	0.809
Amygala (LH)	-0.105	0.560	-0.187	0.852
Caudate (LH)	-0.852	0.972	-0.877	0.380
Hippocampus (LH)	0.174	0.972	0.179	0.858
Left lateral ventricle	-1.083	11.764	-0.092	0.927
Pallidum (LH)	0.761	0.584	1.301	0.193
Putamen (LH)	1.412	1.507	0.937	0.349
Thalamus (LH)	1.907	2.044	0.933	0.351
Accumbens (RH)	0.260	0.374	0.695	0.487
Amygdala (RH)	-0.855	0.815	-1.049	0.294
Caudate (RH)	-0.264	1.070	-0.247	0.805
Hippocampus (RH)	-3.250	1.571	-2.068	0.039
Right lateral ventricle	7.740	14.339	0.540	0.589
Pallidum (RH)	0.511	0.311	1.640	0.101
Putamen (RH)	-0.076	1.908	-0.040	0.968
Thalamus (RH)	1.186	1.543	0.769	0.442
Thickness measures – cortical regions				
L_bankssts	0.00072	0.0003	2.2867	0.0222
L_caudalanteriorcingulate	-0.00021	0.0006	-0.3419	0.7324
L_caudalmiddlefrontal	0.00021	0.0004	0.5335	0.5937
L_cuneus	0.00005	0.0002	0.2098	0.8338
L_entorhinal	-0.00115	0.0008	-1.5244	0.1274
L_frontalpole	0.00047	0.0006	0.8272	0.4081
L_fusiform	0.00025	0.0003	0.9777	0.3282
L_inferiorparietal	0.00002	0.0003	0.0469	0.9626
L_inferiortemporal	0.00012	0.0004	0.3419	0.7324

L_insula	0.00057	0.0003	1.7032	0.0885
L_isthmuscingulate	-0.00016	0.0005	-0.3358	0.7370
L_lateraloccipital	0.00000	0.0002	-0.0104	0.9917
L_lateralorbitofrontal	-0.00057	0.0006	-0.9499	0.3422
L_lingual	0.00016	0.0002	0.7707	0.4409
L_medialorbitofrontal	-0.00007	0.0003	-0.2326	0.8160
L_middletemporal	-0.00010	0.0005	-0.2019	0.8400
L_paracentral	-0.00001	0.0004	-0.0147	0.9883
L_parahippocampal	-0.00005	0.0011	-0.0508	0.9595
L_parsopercularis	-0.00014	0.0005	-0.2676	0.7890
L_parsorbitalis	-0.00002	0.0007	-0.0300	0.9761
L_parstriangularis	-0.00050	0.0010	-0.4935	0.6216
L_pericalcarine	0.00040	0.0002	1.8455	0.0650
L_postcentral	0.00004	0.0003	0.1239	0.9014
L_posteriorcingulate	-0.00007	0.0005	-0.1353	0.8924
L_precentral	0.00004	0.0002	0.1661	0.8681
L_precuneus	0.00008	0.0004	0.2013	0.8405
L_rostralanteriorcingulate	0.00003	0.0005	0.0614	0.9511
L_rostralmiddlefrontal	-0.00031	0.0007	-0.4516	0.6516
L_superiorfrontal	-0.00068	0.0007	-0.9755	0.3293
L_superiorparietal	0.00034	0.0002	1.6793	0.0931
L_superiortemporal	-0.00028	0.0003	-0.9662	0.3339
L_supramarginal	0.00011	0.0004	0.2764	0.7822
L_temporalpole	-0.00047	0.0006	-0.8352	0.4036
L_transversetemporal	-0.00037	0.0005	-0.7720	0.4401
R_bankssts	0.00007	0.0006	0.1080	0.9140
R_caudalanteriorcingulate	-0.00021	0.0007	-0.2760	0.7826
R_caudalmiddlefrontal	0.00004	0.0005	0.0649	0.9482
R_cuneus	0.00020	0.0002	0.8398	0.4010
R_entorhinal	-0.00157	0.0009	-1.8424	0.0654
R_frontalpole	0.00042	0.0007	0.6095	0.5422

R_fusiform	-0.00073	0.0004	-1.7767	0.0756
R_inferiorparietal	-0.00015	0.0003	-0.5601	0.5754
R_inferiortemporal	-0.00042	0.0005	-0.7803	0.4352
R_insula	0.00022	0.0003	0.7864	0.4317
R_isthmuscingulate	0.00021	0.0003	0.6040	0.5458
R_lateraloccipital	-0.00039	0.0004	-1.1210	0.2623
R_lateralorbitofrontal	0.00004	0.0004	0.1003	0.9201
R_lingual	-0.00004	0.0002	-0.2011	0.8406
R_medialorbitofrontal	-0.00016	0.0004	-0.4392	0.6605
R_middletemporal	-0.00039	0.0005	-0.8528	0.3937
R_paracentral	0.00040	0.0003	1.5586	0.1191
R_parahippocampal	-0.00055	0.0006	-0.9803	0.3269
R_parsopercularis	-0.00009	0.0003	-0.3072	0.7587
R_parsorbitalis	0.00069	0.0004	1.6965	0.0898
R_parstriangularis	-0.00008	0.0006	-0.1352	0.8925
R_pericalcarine	0.00034	0.0004	0.8546	0.3928
R_postcentral	0.00023	0.0002	1.0178	0.3088
R_posteriorcingulate	0.00024	0.0003	0.8505	0.3951
R_precentral	0.00005	0.0002	0.2252	0.8218
R_precuneus	0.00013	0.0002	0.5829	0.5600
R_rostralanteriorcingulate	-0.00065	0.0005	-1.1865	0.2354
R_rostralmiddlefrontal	-0.00003	0.0004	-0.0742	0.9408
R_superiorfrontal	0.00001	0.0004	0.0373	0.9703
R_superiorparietal	-0.00004	0.0002	-0.1895	0.8497
R_superiortemporal	-0.00068	0.0003	-2.3695	0.0178
R_supramarginal	-0.00023	0.0004	-0.5600	0.5755
R_temporalpole	-0.00054	0.0010	-0.5589	0.5762
R_transversetemporal	-0.00004	0.0005	-0.0877	0.9302

Abbreviations: Beta, correlation beta; SE, standard error; LH, left hemisphere; RH, right hemisphere. See Supplementary Table 3 for a list of cortical abbreviations. All uncorrected p-values are reported.

Supplementary Table 25. Full meta-analytical results of the age-by-diagnosis interaction test, controlling for sex and ICV | 'All other epilepsies'

Structure	Beta	SE	Z value	*P* value
Volume measures – subcortical regions, hippocampi, and lateral ventricles				
Accumbens (LH)	0.07	0.18	0.37	0.71
Amygala (LH)	-0.71	0.44	-1.59	0.11
Caudate (LH)	-0.30	0.74	-0.40	0.69
Hippocampus (LH)	-0.82	0.85	-0.97	0.33
Left lateral ventricle	-6.47	6.34	-1.02	0.31
Pallidum (LH)	0.10	0.47	0.22	0.82
Putamen (LH)	0.25	1.41	0.18	0.86
Thalamus (LH)	-0.43	2.19	-0.20	0.84
Accumbens (RH)	0.14	0.34	0.42	0.68
Amygdala (RH)	-1.27	0.55	-2.32	0.02
Caudate (RH)	0.16	0.64	0.26	0.80
Hippocampus (RH)	-4.00	1.30	-3.07	0.00
Right lateral ventricle	-0.16	7.80	-0.02	0.98
Pallidum (RH)	0.22	0.27	0.80	0.43
Putamen (RH)	-0.66	1.54	-0.43	0.67
Thalamus (RH)	-0.37	1.51	-0.25	0.81
Thickness measures – cortical regions				
L_bankssts	-0.0007	0.0006	-1.0670	0.2860
L_caudalanteriorcingulate	0.0004	0.0009	0.4353	0.6633
L_caudalmiddlefrontal	0.0000	0.0007	-0.0541	0.9568
L_cuneus	-0.0012	0.0008	-1.4519	0.1465
L_entorhinal	-0.0009	0.0014	-0.6202	0.5351
L_frontalpole	-0.0010	0.0011	-0.8380	0.4020
L_fusiform	-0.0004	0.0005	-0.8112	0.4173
L_inferiorparietal	-0.0010	0.0006	-1.5997	0.1097
L_inferiortemporal	-0.0005	0.0006	-0.8015	0.4228
L_insula	0.0006	0.0005	1.0968	0.2727
L_isthmuscingulate	-0.0009	0.0009	-1.0466	0.2953

L_lateraloccipital	-0.0009	0.0005	-1.8602	0.0629
L_lateralorbitofrontal	-0.0005	0.0009	-0.6283	0.5298
L_lingual	-0.0008	0.0004	-1.8816	0.0599
L_medialorbitofrontal	-0.0006	0.0006	-1.0843	0.2782
L_middletemporal	-0.0007	0.0006	-1.1416	0.2536
L_paracentral	-0.0006	0.0006	-1.0058	0.3145
L_parahippocampal	-0.0008	0.0013	-0.5939	0.5526
L_parsopercularis	-0.0005	0.0007	-0.7387	0.4601
L_parsorbitalis	-0.0009	0.0008	-1.1287	0.2590
L_parstriangularis	-0.0002	0.0008	-0.2078	0.8354
L_pericalcarine	-0.0004	0.0005	-0.6798	0.4966
L_postcentral	-0.0004	0.0004	-0.9053	0.3653
L_posteriorcingulate	-0.0007	0.0006	-1.1893	0.2343
L_precentral	-0.0008	0.0005	-1.5999	0.1096
L_precuneus	-0.0010	0.0007	-1.4655	0.1428
L_rostralanteriorcingulate	0.0002	0.0012	0.1559	0.8761
L_rostralmiddlefrontal	-0.0006	0.0008	-0.7028	0.4822
L_superiorfrontal	-0.0011	0.0009	-1.3072	0.1911
L_superiorparietal	-0.0007	0.0005	-1.5084	0.1315
L_superiortemporal	-0.0017	0.0006	-2.8763	0.0040
L_supramarginal	-0.0008	0.0005	-1.4898	0.1363
L_temporalpole	-0.0022	0.0012	-1.9136	0.0557
L_transversetemporal	-0.0009	0.0007	-1.2276	0.2196
R_bankssts	-0.0012	0.0008	-1.4480	0.1476
R_caudalanteriorcingulate	-0.0005	0.0012	-0.3845	0.7006
R_caudalmiddlefrontal	-0.0001	0.0007	-0.0919	0.9268
R_cuneus	-0.0016	0.0009	-1.8425	0.0654
R_entorhinal	-0.0003	0.0016	-0.2011	0.8406
R_frontalpole	0.0003	0.0012	0.2506	0.8021
R_fusiform	-0.0014	0.0005	-2.5991	0.0093
R_inferiorparietal	-0.0013	0.0006	-2.2270	0.0259
R_inferiortemporal	-0.0010	0.0006	-1.6811	0.0927

R_insula	-0.0003	0.0006	-0.5816	0.5608
R_isthmuscingulate	-0.0010	0.0009	-1.0593	0.2895
R_lateraloccipital	-0.0015	0.0005	-2.9762	0.0029
R_lateralorbitofrontal	-0.0005	0.0009	-0.5337	0.5935
R_lingual	-0.0012	0.0007	-1.7948	0.0727
R_medialorbitofrontal	-0.0002	0.0006	-0.2774	0.7815
R_middletemporal	-0.0013	0.0008	-1.7078	0.0877
R_paracentral	0.0003	0.0007	0.3688	0.7122
R_parahippocampal	-0.0008	0.0010	-0.8312	0.4059
R_parsopercularis	-0.0004	0.0008	-0.4805	0.6309
R_parsorbitalis	0.0008	0.0009	0.8364	0.4029
R_parstriangularis	0.0000	0.0007	0.0442	0.9647
R_pericalcarine	-0.0013	0.0005	-2.6732	0.0075
R_postcentral	-0.0005	0.0005	-1.0434	0.2968
R_posteriorcingulate	0.0001	0.0005	0.2492	0.8032
R_precentral	-0.0005	0.0005	-1.1036	0.2698
R_precuneus	-0.0012	0.0008	-1.4186	0.1560
R_rostralanteriorcingulate	-0.0013	0.0008	-1.6635	0.0962
R_rostralmiddlefrontal	-0.0001	0.0007	-0.1245	0.9009
R_superiorfrontal	-0.0001	0.0008	-0.1708	0.8643
R_superiorparietal	-0.0009	0.0005	-1.8607	0.0628
R_superiortemporal	-0.0018	0.0006	-3.1373	0.0017
R_supramarginal	-0.0009	0.0005	-1.6960	0.0899
R_temporalpole	0.0006	0.0016	0.4052	0.6853
R_transversetemporal	-0.0003	0.0008	-0.4334	0.6648

Abbreviations: Beta, correlation beta; LH, left hemisphere; RH, right hemisphere. See Supplementary Table 3 for a list of cortical abbreviations. All uncorrected p-values are reported.

Supplementary Table 26. Full list of case-versus-control effect sizes for subcortical volume differences, including non-significant findings | 'All epilepsies' phenotype

Structure	Cohen's *d*	SE	Z score	95% CI	*P* value	FDR-adjusted *P* value	I^2	Number of controls	Number of cases
**Thalamus (RH)***	**-0.37**	**0.05**	**-7.48**	**-0.46 - -0.27**	**$7.67x10^{-14}$**	**1.29X10-12**	**44.82**	**1716**	**2137**
**Thalamus (LH)***	**-0.36**	**0.07**	**-4.84**	**-0.5 - -0.21**	**$1.31x10^{-6}$**	**6.88X10-6**	**75.65**	**1687**	**2104**
**Hippocampus (LH)***	**-0.35**	**0.07**	**-5.12**	**-0.49 - -0.22**	**$3.04x10^{-7}$**	**1.82X10-6**	**71.84**	**1707**	**2125**
**Hippocampus (RH)***	**-0.34**	**0.05**	**-6.17**	**-0.44 - -0.23**	**$6.63x10^{-10}$**	**7.96X10-9**	**54.80**	**1719**	**2129**
**Pallidum (RH)***	**-0.32**	**0.05**	**-5.76**	**-0.42 - -0.21**	**$8.32x10^{-9}$**	**6.99X10-8**	**55.58**	**1710**	**2112**
**Putamen (LH)***	**-0.25**	**0.06**	**-3.94**	**-0.37 - -0.12**	**$8.30x10^{-5}$**	**2.91X10-4**	**65.34**	**1636**	**2075**
**Putamen (RH)**	-0.24	0.08	-3.17	-0.39 - -0.09	$1.53x10^{-3}$	**3.89X10-3**	76.74	1690	2127
**Pallidum (LH)**	-0.17	0.08	-2.11	-0.33 - -0.01	$3.53x10^{-2}$	5.47X10-2	79.55	1609	1991
**Caudate (LH)**	-0.12	0.05	-2.42	-0.22 - -0.02	$1.56x10^{-2}$	2.79X10-2	47.69	1724	2135
**Caudate (RH)**	-0.08	0.05	-1.49	-0.19 - 0.03	$1.36x10^{-1}$	1.74X10-1	54.70	1725	2137
**Accumbens (RH)**	-0.06	0.09	-0.68	-0.25 - 0.12	$4.94x10^{-1}$	5.39X10-1	85.16	1702	2137
**Accumbens (LH)**	-0.02	0.08	-0.21	-0.17 - 0.14	$8.33x10^{-1}$	8.54X10-1	79.23	1707	2136
**Amygdala (LH)**	0.10	0.07	1.45	-0.04 - 0.24	$1.47x10^{-1}$	1.79X10-1	73.59	1717	2124
**Amygdala (RH)**	0.11	0.06	1.83	-0.01 - 0.23	$6.76x10^{-2}$	9.62X10-2	63.44	1693	2108
**Right lateral ventricle***	**0.27**	**0.03**	**7.86**	**0.2 - 0.33**	**$3.73x10^{-15}$**	**7.83X10-14**	**0.00**	**1722**	**2137**
**Left lateral ventricle***	**0.29**	**0.04**	**7.02**	**0.21 - 0.37**	**$2.14x10^{-12}$**	**3.00X10-11**	**23.34**	**1722**	**2135**

Abbreviations: LH, left hemisphere; RH, right hemisphere; SE, standard error; CI, confidence interval; I^2, heterogeneity index. All *p*-values are reported, uncorrected. *P*-values that passed the Bonferroni-adjusted alpha level ($p<1.49x10^{-4}$) are highlighted in bold red (see Methods for statistical threshold determination). *P*-values that passed adjustment for false discovery rate (FDR) are highlighted in bold.

Supplementary Table 27. Full list of case-versus-control effect sizes for subcortical volume differences, including non-significant findings | 'MTLE-L' phenotype

Structure	Cohen's *d*	SE	Z score	95% CI	*P* value	FDR-adjusted *P* value	I^2	Number of controls	Number of cases
**Hippocampus (LH)***	-1.73	0.19	-9.06	-2.1 - -1.35	**$1.35x10^{-19}$**	**1.13E-17**	85.53	1412	410
**Thalamus (LH)***	-0.84	0.13	-6.69	-1.09 - -0.6	**$2.19x10^{-11}$**	**6.13X10-10**	70.46	1384	408
**Thalamus (RH)***	-0.46	0.12	-3.94	-0.69 - -0.23	**$8.12x10^{-5}$**	**3.25X10-4**	67.38	1412	414
**Pallidum (RH)***	-0.45	0.09	-5.01	-0.63 - -0.28	**$5.48x10^{-7}$**	**3.54X10-6**	43.98	1406	414
**Putamen (LH)***	-0.39	0.08	-4.88	-0.54 - -0.23	**$1.07x10^{-6}$**	**6.42X10-6**	28.47	1352	410
**Putamen (RH)**	-0.28	0.11	-2.53	-0.5 - -0.06	$1.13x10^{-2}$	**2.43X10-2**	64.26	1386	412
**Pallidum (LH)**	-0.27	0.15	-1.80	-0.56 - 0.02	$7.25x10^{-2}$	1.11X10-1	80.31	1332	398
**Amygdala (LH)**	-0.22	0.09	-2.32	-0.4 - -0.03	$2.03x10^{-2}$	**3.88X10-2**	49.18	1413	412
**Hippocampus (RH)**	-0.20	0.14	-1.41	-0.49 - 0.08	$1.59x10^{-1}$	2.09X10-1	79.22	1417	414
**Caudate (LH)**	-0.18	0.12	-1.55	-0.41 - 0.05	$1.22x10^{-1}$	1.69X10-1	68.17	1418	414
**Accumbens (RH)**	-0.12	0.16	-0.76	-0.44 - 0.19	$4.47x10^{-1}$	4.94X10-1	83.08	1396	413
**Accumbens (LH)**	-0.10	0.13	-0.78	-0.36 - 0.15	$4.36x10^{-1}$	4.88X10-1	74.43	1402	413
**Caudate (RH)**	-0.06	0.12	-0.54	-0.29 - 0.16	$5.87x10^{-1}$	6.16X10-1	67.36	1419	413
**Amygdala (RH)**	0.27	0.09	2.93	0.09 - 0.44	$3.42x10^{-3}$	**8.71X10-3**	44.66	1387	398
**Right lateral ventricle***	0.36	0.09	3.92	0.18 - 0.54	**$8.95x10^{-5}$**	**3.42X10-4**	47.23	1418	414
**Left lateral ventricle***	0.47	0.09	5.20	0.29 - 0.64	**$1.96x10^{-7}$**	**1.83X10-6**	43.12	1417	414

Abbreviations: LH, left hemisphere; RH, right hemisphere; SE, standard error; CI, confidence interval; I^2, heterogeneity index. All *p*-values are reported, uncorrected. *P*-values that passed the Bonferroni-adjusted alpha level ($p<1.49x10^{-4}$) are highlighted in bold red (see Methods for statistical threshold determination). *P*-values that passed adjustment for false discovery rate (FDR) are highlighted in bold.

Supplementary Table 28. Full list of case-versus-control effect sizes for subcortical volume differences, including non-significant findings | 'MTLE-R' phenotype

Structure	Cohen's *d*	SE	Z score	95% CI	*P* value	FDR-adjusted *P* value	I^2	Number of controls	Number of cases
**Hippocampus (RH)***	-1.906	0.150	-12.694	-2.2 - -1.61	$6.36x10^{-37}$	**5.34E-35**	72.476	1286	336
**Thalamus (RH)***	-0.727	0.103	-7.066	-0.93 - -0.53	$1.60x10^{-12}$	**6.72X10-11**	51.499	1285	335
**Thalamus (LH)**	-0.472	0.209	-2.256	-0.88 - -0.06	$2.41x10\text{-}^{2}$	5.23X10-2	88.785	1255	316
**Putamen (RH)***	-0.470	0.135	-3.484	-0.73 - -0.21	$4.94x10\text{-}^{4}$	**2.18X10-3**	72.787	1258	337
**Pallidum (RH)***	-0.451	0.089	-5.071	-0.62 - -0.28	$3.96x10\text{-}^{7}$	**5.54X10-6**	36.432	1278	332
**Putamen (LH)**	-0.302	0.127	-2.382	-0.55 - -0.05	$1.72x10\text{-}^{2}$	**4.13X10-2**	69.055	1204	330
**Pallidum (LH)**	-0.298	0.122	-2.450	-0.54 - -0.06	$1.43x10\text{-}^{2}$	**3.64X10-2**	65.376	1177	319
**Caudate (LH)**	-0.196	0.091	-2.152	-0.37 - -0.02	$3.14x10\text{-}^{2}$	6.43X10-2	41.090	1291	337
**Caudate (RH)**	-0.191	0.100	-1.900	-0.39 - 0.01	$5.74x10\text{-}^{2}$	9.84X10-2	51.200	1292	337
**Hippocampus (LH)**	-0.169	0.088	-1.913	-0.34 - 0	$5.57x10\text{-}^{2}$	9.75X10-2	37.104	1277	337
**Accumbens (RH)**	-0.026	0.135	-0.195	-0.29 - 0.24	$8.45x10\text{-}^{1}$	8.62X10-1	73.035	1269	336
**Amygdala (RH)**	0.027	0.121	0.221	-0.21 - 0.26	$8.25x10\text{-}^{1}$	8.56X10-1	66.130	1260	331
**Accumbens (LH)**	0.055	0.093	0.586	-0.13 - 0.24	$5.58x10\text{-}^{1}$	6.42X10-1	43.313	1275	336
**Amygdala (LH)**	0.228	0.080	2.860	0.07 - 0.38	$4.24x10\text{-}^{3}$	**1.48X10-2**	24.820	1286	334
**Left lateral ventricle***	0.390	0.081	4.808	0.23 - 0.55	$1.52x10\text{-}^{6}$	**1.60X10-5**	26.750	1291	338
**Right lateral ventricle***	0.444	0.065	6.867	0.32 - 0.57	$6.57x10^{-12}$	**1.84X10-10**	0.003	1292	338

Abbreviations: LH, left hemisphere; RH, right hemisphere; SE, standard error; CI, confidence interval; I^2, heterogeneity index. All *p*-values are reported, uncorrected. *P*-values that passed the Bonferroni-adjusted alpha level ($p<1.49x10^{-4}$) are highlighted in bold red (see Methods for statistical threshold determination). *P*-values that passed adjustment for false discovery rate (FDR) are highlighted in bold.

Supplementary Table 29. Full list of case-versus-control effect sizes for subcortical volume differences, including non-significant findings | 'IGE' phenotype

Structure	Cohen's *d*	SE	Z score	95% CI	*P* value	FDR-adjusted *P* value	I^2	Number of controls	Number of cases
**Thalamus (RH)***	-0.403	0.087	-4.633	-0.57-0.23	**3.60x10-^{6}**	**1.0X10-4**	39.715	1210	363
**Thalamus (LH)**	-0.401	0.135	-2.969	-0.67-0.14	2.99x10-^{3}	**2.8X10-2**	74.334	1181	360
**Pallidum (RH)***	-0.349	0.097	-3.585	-0.54-0.16	**3.37x10-^{4}**	**7.1X10-3**	51.278	1204	363
**Hippocampus (LH)**	-0.319	0.136	-2.345	-0.59-0.05	1.90x10-^{2}	1.1X10-1	74.899	1204	363
**Putamen (LH)**	-0.285	0.089	-3.212	-0.46-0.11	1.32x10-^{3}	**1.6X10-2**	39.930	1130	353
**Caudate (LH)**	-0.248	0.076	-3.267	-0.4-0.1	1.09x10-^{3}	**1.5X10-2**	23.174	1218	362
**Hippocampus (RH)**	-0.247	0.133	-1.856	-0.510.01	6.35x10-^{2}	2.7X10-1	73.934	1214	361
**Putamen (RH)**	-0.234	0.113	-2.061	-0.46-0.01	3.93x10-^{2}	2.1X10-1	63.221	1186	358
**Caudate (RH)**	-0.198	0.081	-2.425	-0.36-0.04	1.53x10-^{2}	9.9X10-2	32.687	1219	363
**Pallidum (LH)**	-0.159	0.184	-0.868	-0.520.2	3.85x10-^{1}	7.0X10-1	85.876	1103	344
**Accumbens (RH)**	-0.148	0.086	-1.706	-0.320.02	8.81x10-^{2}	3.2X10-1	39.638	1199	364
**Accumbens (LH)**	-0.131	0.098	-1.346	-0.320.06	1.78x10-^{1}	4.7X10-1	51.808	1204	364
**Amygdala (RH)**	-0.122	0.091	-1.347	-0.30.06	1.78x10-^{1}	4.7X10-1	44.927	1187	363
**Amygdala (LH)**	-0.050	0.134	-0.372	-0.310.21	7.10x10-^{1}	8.9X10-1	73.766	1214	353
**Left lateral ventricle**	0.237	0.085	2.795	0.070.4	5.20x10-^{3}	**4.0X10-2**	36.912	1216	360
**Right lateral ventricle**	0.265	0.080	3.313	0.110.42	9.23x10-^{4}	**1.5X10-2**	29.877	1216	362

Abbreviations: LH, left hemisphere; RH, right hemisphere; SE, standard error; CI, confidence interval; I^2, heterogeneity index. All *p*-values are reported, uncorrected. *P*-values that passed the Bonferroni-adjusted alpha level ($p<1.49x10^{-4}$) are highlighted in bold red (see Methods for statistical threshold determination). *P*-values that passed adjustment for false discovery rate (FDR) are highlighted in bold.

Supplementary Table 30. Full list of case-versus-control effect sizes for subcortical volume differences, including non-significant findings | 'All other epilepsies' phenotype

Structure	Cohen's *d*	SE	Z score	95% CI	*P* value	FDR-adjusted *P* value	I^2	Number of controls	Number of cases
**Thalamus (RH)***	-0.305	0.047	-6.502	-0.397 - -0.213	**7.92x10-11**	1.66X10-9	4.985	1446	998
**Pallidum (RH)***	-0.235	0.060	-3.942	-0.352 - -0.118	**8.07x10-05**	5.30X10-6	36.141	1440	976
**Thalamus (LH)**	-0.197	0.079	-2.479	-0.353 - -0.041	0.0132	2.77X10-5	63.279	1417	993
**Putamen (LH)**	-0.193	0.082	-2.361	-0.353 - -0.033	0.0182	5.74X10-5	64.184	1366	955
**Pallidum (LH)**	-0.208	0.090	-2.303	-0.385 - -0.031	0.0213	3.08X10-4	70.311	1339	903
**Putamen (RH)**	-0.166	0.083	-1.998	-0.329 - -0.003	0.0457	5.11X10-4	66.530	1420	993
**Caudate (LH)**	-0.070	0.059	-1.190	-0.185 - 0.045	0.2341	2.84X10-2	34.736	1453	995
**Hippocampus (RH)**	-0.041	0.046	-0.875	-0.131 - 0.05	0.3815	3.73X10-2	3.179	1448	991
**Hippocampus (LH)**	-0.021	0.056	-0.374	-0.132 - 0.09	0.7084	4.16X10-2	29.722	1439	988
**Accumbens (RH)**	0.016	0.091	0.171	-0.163 - 0.194	0.8642	7.83X10-2	72.286	1431	997
**Caudate (RH)**	-0.009	0.062	-0.149	-0.131 - 0.112	0.8813	2.98X10-1	41.439	1454	997
**Accumbens (LH)**	-0.011	0.096	-0.111	-0.199 - 0.178	0.9118	4.58X10-1	75.051	1437	996
**Left lateral ventricle***	0.198	0.045	4.373	0.109 - 0.287	**1.23x10-05**	8.15X10-1	0.218	1452	996
**Right lateral ventricle***	0.212	0.046	4.581	0.122 - 0.303	**4.62x10-06**	9.18X10-1	3.528	1453	996
**Amygdala (RH)***	0.218	0.057	3.799	0.106 - 0.33	**1.46x10-04**	9.18X10-1	31.256	1422	989
**Amygdala (LH)***	0.327	0.065	5.024	0.199 - 0.455	**5.05x10-07**	9.18X10-1	45.470	1448	998

Abbreviations: LH, left hemisphere; RH, right hemisphere; SE, standard error; CI, confidence interval; I^2, heterogeneity index. All *p*-values are reported, uncorrected. *P*-values that passed the Bonferroni-adjusted alpha level ($p<1.49\times10^{-4}$) are highlighted in bold red (see Methods for statistical threshold determination).

Supplementary Table 31. Full list of case-versus-control effect sizes for cortical thickness differences, including nominal and non-significant findings | 'All epilepsies' phenotype

Structure	Cohen's *d*	SE	Z score	95% CI	*P* value	FDR-adjusted *P* value	I^2	Number of controls	Number of cases
R_precentral*	-0.40	0.04	-9.10	-0.49 - -0.31	**8.85x10-20**	**7.43E-18**	27.93	1649	2054
L_precentral*	-0.38	0.04	-8.77	-0.47 - -0.3	**1.82x10-18**	**7.64E-17**	27.65	1645	2058
L_caudalmiddlefrontal*	-0.32	0.04	-7.93	-0.4 - -0.24	**2.11x10-15**	**5.91X10-14**	17.11	1650	2061
R_paracentral*	-0.32	0.05	-5.98	-0.42 - -0.21	**2.19x10-9**	**2.04X10-8**	49.26	1654	2059
R_superiorparietal	-0.31	0.09	-3.35	-0.5 - -0.13	8.13x10-4	**2.14X10-3**	84.46	1653	2058
L_paracentral	-0.31	0.07	-4.75	-0.44 - -0.18	**2.05x10-6**	**1.01X10-5**	67.48	1650	2061
R_caudalmiddlefrontal*	-0.31	0.05	-5.99	-0.41 - -0.21	**2.09x10-9**	**2.04X10-8**	46.44	1653	2059
L_superiorparietal	-0.29	0.08	-3.75	-0.44 - -0.14	**1.78x10-4**	**5.54X10-4**	76.42	1645	2058
L_superiorfrontal*	-0.28	0.05	-5.25	-0.39 - -0.18	**1.51x10-7**	**9.76X10-7**	51.77	1649	2059
R_precuneus*	-0.28	0.07	-4.20	-0.4 - -0.15	**2.70x10-5**	**1.07X10-4**	67.61	1654	2055
R_superiorfrontal*	-0.27	0.06	-4.59	-0.38 - -0.15	**4.49x10-6**	**2.10X10-5**	59.48	1650	2058
L_entorhinal*	-0.26	0.06	-4.26	-0.39 - -0.14	**2.04x10-5**	**8.57X10-5**	56.65	1402	1724
L_precuneus	-0.25	0.08	-3.00	-0.41 - -0.09	2.66x10-3	**6.04X10-3**	79.94	1649	2059
L_supramarginal*	-0.23	0.06	-3.89	-0.35 - -0.12	**9.87x10-5**	**3.32X10-4**	59.39	1606	1965
R_supramarginal*	-0.22	0.06	-4.04	-0.33 - -0.12	**5.24x10-5**	**1.91X10-4**	52.90	1597	1971
L_temporalpole	-0.20	0.06	-3.37	-0.32 - -0.09	7.49x10-4	**2.10X10-3**	62.12	1643	2055
R_cuneus*	-0.20	0.04	-5.33	-0.28 - -0.13	**9.68x10-8**	**6.78X10-7**	11.42	1651	2057
R_lateraloccipital	-0.20	0.06	-3.13	-0.32 - -0.07	1.77x10-3	**4.37X10-3**	65.81	1653	2058
L_middletemporal	-0.20	0.07	-2.88	-0.33 - -0.06	4.01x10-3	**8.64X10-3**	69.58	1546	1955
R_parstriangularis*	-0.20	0.04	-5.48	-0.27 - -0.13	**4.25x10-8**	**3.25X10-7**	4.66	1652	2058
L_inferiorparietal	-0.20	0.07	-2.79	-0.34 - -0.06	5.29x10-3	**1.08X10-2**	72.32	1639	2052
R_rostralmiddlefrontal	-0.20	0.06	-3.43	-0.31 - -0.08	5.94x10-4	**1.72X10-3**	57.60	1654	2058

**L_transversetemporal***	-0.19	0.04	-4.41	-0.28 - -0.11	**$1.05x10^{-5}$**	**4.64X10-5**	28.18	1647	2061
**L_parstriangularis***	-0.19	0.05	-3.83	-0.29 - -0.09	**$1.29x10^{-4}$**	**4.17X10-4**	44.41	1650	2060
L_lateraloccipital	-0.19	0.09	-2.10	-0.37 - -0.01	$3.58x10^{-2}$	5.47X10-2	83.47	1650	2060
**L_fusiform**	-0.19	0.06	-3.08	-0.31 - -0.07	$2.10x10^{-3}$	**5.04X10-3**	62.37	1644	2058
**R_transversetemporal***	**-0.18**	**0.04**	**-4.19**	**-0.27 - -0.1**	**$2.81x10^{-5}$**	**1.07X10-4**	**27.92**	**1654**	**2059**
**R_temporalpole**	**-0.18**	**0.05**	**-3.45**	**-0.28 - -0.08**	**$5.51x10^{-4}$**	**1.65X10-3**	48.99	1648	2048
**L_superiortemporal**	-0.18	0.08	-2.22	-0.34 - -0.02	$2.62x10^{-2}$	**4.23X10-2**	77.96	1533	1937
L_lingual	-0.18	0.08	-2.11	-0.34 - -0.01	$3.50x10^{-2}$	**5.47X10-2**	81.01	1649	2061
**R_parsopercularis***	**-0.18**	**0.04**	**-4.98**	**-0.25 - -0.11**	**$6.48x10^{-7}$**	**3.63X10-6**	**2.62**	**1652**	**2059**
**R_inferiorparietal**	-0.17	0.07	-2.30	-0.32 - -0.03	$2.15x10^{-2}$	**3.61X10-2**	75.03	1643	2045
**R_lingual**	-0.16	0.05	-3.35	-0.26 - -0.07	$8.14x10^{-4}$	**2.14X10-3**	40.47	1653	2056
**R_entorhinal**	-0.16	0.06	-2.86	-0.27 - -0.05	$4.24x10^{-3}$	**8.90X10-3**	45.90	1373	1691
L_cuneus	-0.16	0.09	-1.79	-0.33 - 0.01	$7.27x10^{-2}$	1.02X10-1	82.33	1648	2061
**L_parsopercularis**	-0.15	0.06	-2.69	-0.26 - -0.04	$7.12x10^{-3}$	**1.39X10-2**	56.64	1648	2059
**L_parsorbitalis**	-0.15	0.05	-3.04	-0.25 - -0.05	$2.37x10^{-3}$	**5.53X10-3**	42.30	1650	2059
**L_rostralmiddlefrontal**	-0.15	0.06	-2.30	-0.27 - -0.02	$2.15x10^{-2}$	**3.61X10-2**	65.22	1649	2057
**R_parsorbitalis**	-0.14	0.05	-2.64	-0.25 - -0.04	$8.27x10^{-3}$	**1.58X10-2**	51.31	1654	2057
**R_pericalcarine**	-0.13	0.05	-2.61	-0.23 - -0.03	$9.15x10^{-3}$	**1.67X10-2**	44.34	1654	2056
L_pericalcarine	-0.13	0.09	-1.46	-0.3 - 0.04	$1.43x10^{-1}$	1.79X10-1	82.76	1650	2059
L_postcentral	-0.13	0.09	-1.38	-0.3 - 0.05	$1.67x10^{-1}$	2.00X10-1	83.46	1647	2056
**R_middletemporal**	-0.12	0.04	-2.97	-0.21 - -0.04	$3.02x10^{-3}$	**6.68X10-3**	22.16	1642	2050
R_superiortemporal	-0.12	0.06	-2.07	-0.24 - -0.01	$3.84x10^{-2}$	5.70X10-2	58.96	1555	1958
R_postcentral	-0.12	0.10	-1.15	-0.31 - 0.08	$2.49x10^{-1}$	2.87X10-1	86.70	1648	2057
**R_fusiform**	-0.12	0.04	-2.63	-0.2 - -0.03	$8.56x10^{-3}$	**1.60X10-2**	29.46	1650	2057
R_lateralorbitofrontal	-0.11	0.05	-2.07	-0.21 - -0.01	$3.87x10^{-2}$	5.70X10-2	50.33	1651	2057
**R_frontalpole**	-0.11	0.05	-2.26	-0.2 - -0.01	$2.37x10^{-2}$	**3.90X10-2**	40.18	1654	2057
**L_parahippocampal**	-0.10	0.04	-2.36	-0.19 - -0.02	$1.83x10^{-2}$	**3.20X10-2**	28.98	1637	2050

**R_parahippocampal**	-0.09	0.03	-2.70	-0.16 - -0.03	6.95x10^{-3}	**1.39X10-2**	0.00	1650	2053
L_bankssts	-0.09	0.08	-1.16	-0.25 - 0.06	2.45x10^{-1}	2.86X10-1	76.88	1584	1927
L_inferiortemporal	-0.09	0.06	-1.49	-0.2 - 0.03	1.37x10^{-1}	1.74X10-1	57.73	1648	2055
L_posteriorcingulate	-0.08	0.04	-1.88	-0.17 - 0	6.01x10^{-2}	8.70X10-2	30.06	1650	2059
L_frontalpole	-0.08	0.05	-1.63	-0.17 - 0.02	1.04x10^{-1}	1.39X10-1	39.69	1648	2058
R_inferiortemporal	-0.07	0.04	-1.73	-0.15 - 0.01	8.36x10^{-2}	1.15X10-1	22.58	1652	2057
R_posteriorcingulate	-0.07	0.06	-1.11	-0.19 - 0.05	2.65x10^{-1}	3.01X10-1	64.86	1654	2056
L_lateralorbitofrontal	-0.07	0.05	-1.45	-0.16 - 0.02	1.46x10^{-1}	1.79X10-1	33.61	1645	2060
R_bankssts	-0.06	0.06	-0.90	-0.18 - 0.07	3.66x10^{-1}	4.05X10-1	66.15	1621	2023
R_medialorbitofrontal	-0.05	0.03	-1.53	-0.12 - 0.02	1.26x10^{-1}	1.65X10-1	0.00	1636	2025
L_isthmuscingulate	-0.05	0.04	-1.37	-0.12 - 0.02	1.72x10^{-1}	2.03X10-1	4.69	1649	2057
R_insula	-0.02	0.05	-0.46	-0.11 - 0.07	6.42x10^{-1}	6.91X10-1	35.96	1615	2032
L_rostralanteriorcingulate	-0.01	0.06	-0.21	-0.13 - 0.1	8.34x10^{-1}	8.54X10-1	58.16	1645	2048
L_medialorbitofrontal	0.01	0.03	0.15	-0.06 - 0.07	8.82x10^{-1}	8.82X10-1	0.00	1632	2030
R_isthmuscingulate	0.01	0.05	0.18	-0.09 - 0.11	8.56x10^{-1}	8.66X10-1	43.20	1651	2057
L_insula	0.01	0.05	0.29	-0.08 - 0.11	7.73x10^{-1}	8.22X10-1	37.86	1610	2026
R_caudalanteriorcingulate	0.01	0.06	0.22	-0.11 - 0.14	8.24x10^{-1}	8.54X10-1	64.37	1652	2056
L_caudalanteriorcingulate	0.04	0.04	0.92	-0.04 - 0.12	3.59x10^{-1}	4.02X10-1	25.12	1645	2045
R_rostralanteriorcingulate	0.09	0.05	1.71	-0.01 - 0.2	8.64x10^{-2}	1.17X10-1	52.42	1652	2055

Abbreviations: RH, right hemisphere, LH, left hemisphere, SE, standard error; CI, confidence interval; I^2, heterogeneity index. Abbreviations for all cortical brain regions are provided in Supplementary Table 4. Uncorrected *p*-values are reported. Brain regions are ranked according to effect size (negative to positive). Uncorrected *P*-values are listed in the '*P*-values' column. *P*-values were compared to an adjusted alpha level of $p<1.49 \times 10^{-4}$. Significant brain regions are **highlighted in red with an asterisk (*)**. *P*-values surviving FDR correction are **highlighted in bold.**

Supplementary Table 32. Full list of case-versus-control effect sizes for cortical thickness differences, including non-significant findings | 'MTLE-L' phenotype

Structure	Cohen's *d*	SE	Z score	95% CI	*P* value	FDR-adjusted *P* value	I^2	Number of controls	Number of cases
**L_precuneus***	-0.54	0.14	-3.96	-0.8 - -0.27	**7.35x10^{-5}**	**3.087X10-4**	75.18	1343	412
**R_precentral***	-0.49	0.08	-6.34	-0.64 - -0.34	**2.37x10^{-10}**	**4.980X10-9**	26.33	1345	412
**R_superiorparietal**	-0.47	0.16	-2.97	-0.79 - -0.16	2.97x10^{-3}	**7.796X10-3**	82.40	1347	412
**R_precuneus***	-0.47	0.10	-4.56	-0.68 - -0.27	**5.16x10^{-6}**	**2.550X10-5**	57.50	1348	412
**L_superiorparietal**	-0.47	0.14	-3.31	-0.75 - -0.19	9.17x10^{-4}	**2.963X10-3**	77.79	1340	412
**L_precentral***	-0.47	0.08	-5.76	-0.63 - -0.31	**8.64x10^{-9}**	**9.070X10-8**	31.60	1339	412
**L_entorhinal***	-0.44	0.07	-6.16	-0.59 - -0.3	**7.35x10^{-10}**	**1.230X10-8**	0.00	1102	303
**R_caudalmiddlefrontal***	-0.44	0.09	-5.09	-0.61 - -0.27	**3.61x10^{-7}**	**2.760X10-6**	39.44	1348	412
**L_paracentral***	-0.43	0.10	-4.31	-0.62 - -0.23	**1.61x10^{-5}**	**7.510X10-5**	53.17	1344	412
**L_superiorfrontal***	-0.41	0.06	-6.80	-0.53 - -0.29	**1.02x10^{-11}**	**4.280X10-10**	0.00	1343	412
**L_caudalmiddlefrontal***	-0.40	0.07	-5.79	-0.54 - -0.27	**7.07x10^{-9}**	**8.480X10-8**	13.81	1344	412
**L_superiortemporal**	-0.40	0.11	-3.61	-0.62 - -0.18	3.11x10^{-4}	**1.136X10-3**	61.22	1228	387
**R_paracentral***	-0.38	0.08	-5.02	-0.53 - -0.23	**5.14x10^{-7}**	**3.540X10-6**	23.54	1348	412
**R_superiorfrontal***	-0.36	0.06	-6.05	-0.48 - -0.25	**1.44x10^{-9}**	**2.020X10-8**	0.00	1345	412
**L_fusiform***	-0.36	0.07	-5.18	-0.49 - -0.22	**2.19x10^{-7}**	**1.840X10-6**	13.46	1339	412
**L_lingual**	-0.35	0.13	-2.66	-0.61 - -0.09	7.89x10^{-3}	**1.821X10-2**	74.50	1343	412
**R_postcentral**	-0.33	0.14	-2.36	-0.61 - -0.06	1.83x10^{-2}	**3.575X10-2**	77.54	1344	412
**L_postcentral**	-0.33	0.14	-2.39	-0.6 - -0.06	1.67x10^{-2}	**3.421X10-2**	76.12	1341	411
**L_temporalpole***	-0.31	0.07	-4.65	-0.45 - -0.18	**3.33x10^{-6}**	**1.750X10-5**	10.90	1341	410
**L_lateraloccipital**	-0.31	0.15	-2.07	-0.61 - -0.02	3.88x10^{-2}	**6.651X10-2**	80.53	1344	412
**R_supramarginal**	-0.30	0.09	-3.41	-0.48 - -0.13	6.53x10^{-4}	**2.286X10-3**	41.23	1292	400

L_parahippocampal*	-0.30	0.07	-4.11	-0.44 - -0.16	**$3.95x10^{-5}$**	**1.746X10-4**	19.37	1335	410
L_middletemporal	-0.29	0.09	-3.02	-0.47 - -0.1	$2.50x10^{-3}$	**7.140X10-3**	47.68	1241	386
L_supramarginal	-0.29	0.10	-2.97	-0.47 - -0.1	$2.95x10^{-3}$	**7.796X10-3**	49.78	1300	395
R_parstriangularis*	-0.28	0.06	-4.74	-0.4 - -0.17	**$2.16x10^{-6}$**	**1.210X10-5**	0.00	1346	412
L_parstriangularis	-0.28	0.09	-3.05	-0.46 - -0.1	$2.30x10^{-3}$	**6.900X10-3**	47.12	1344	411
R_inferiorparietal	-0.28	0.12	-2.41	-0.51 - -0.05	$1.61x10^{-2}$	**3.381X10-2**	66.56	1337	411
R_lateraloccipital	-0.25	0.12	-2.09	-0.49 - -0.02	$3.64x10^{-2}$	6.506X10-2	69.72	1347	412
L_inferiorparietal	-0.24	0.13	-1.87	-0.5 - 0.01	$6.13x10^{-2}$	9.715X10-2	73.10	1333	410
R_rostralmiddlefrontal	-0.24	0.09	-2.55	-0.43 - -0.06	$1.07x10^{-2}$	**2.365X10-2**	49.71	1348	412
L_inferiortemporal	-0.21	0.07	-2.77	-0.35 - -0.06	$5.65x10^{-3}$	**1.396X10-2**	22.59	1342	412
L_rostralmiddlefrontal	-0.20	0.10	-2.01	-0.4 - -0.01	$4.41x10^{-2}$	7.409X10-2	55.49	1344	412
L_parsopercularis	-0.20	0.09	-2.11	-0.39 - -0.01	$3.50x10^{-2}$	6.506X10-2	49.80	1342	411
R_cuneus	-0.20	0.07	-2.65	-0.35 - -0.05	$8.02x10^{-3}$	**1.821X10-2**	23.09	1345	412
L_pericalcarine	-0.20	0.12	-1.62	-0.44 - 0.04	$1.05x10^{-1}$	1.521X10-1	70.05	1344	411
R_fusiform	-0.20	0.06	-3.29	-0.31 - -0.08	$1.00x10^{-3}$	**3.111X10-3**	0.00	1344	412
L_cuneus	-0.19	0.13	-1.50	-0.44 - 0.06	$1.34x10^{-1}$	1.815X10-1	72.19	1342	412
R_superiortemporal	-0.16	0.06	-2.68	-0.28 - -0.04	$7.44x10^{-3}$	**1.786X10-2**	0.00	1249	398
R_pericalcarine	-0.16	0.08	-2.08	-0.32 - -0.01	$3.73x10^{-2}$	6.528X10-2	28.72	1348	411
R_transversetemporal	-0.16	0.07	-2.38	-0.29 - -0.03	$1.72x10^{-2}$	**3.440X10-2**	11.12	1348	412
R_parsopercularis	-0.16	0.08	-1.94	-0.31 - 0	$5.21x10^{-2}$	8.578X10-2	31.30	1347	412
R_lingual	-0.16	0.10	-1.58	-0.35 - 0.04	$1.14x10^{-1}$	1.623X10-1	53.53	1348	412
R_posteriorcingulate	-0.15	0.10	-1.54	-0.34 - 0.04	$1.23x10^{-1}$	1.694X10-1	52.26	1348	411
L_parsorbitalis	-0.15	0.09	-1.64	-0.33 - 0.03	$1.02x10^{-1}$	1.503X10-1	46.44	1344	412
L_transversetemporal	-0.14	0.07	-2.10	-0.27 - -0.01	$3.58x10^{-2}$	6.506X10-2	10.53	1341	412
R_parsorbitalis	-0.14	0.08	-1.80	-0.29 - 0.01	$7.20x10^{-2}$	1.107X10-1	26.87	1348	412
R_parahippocampal	-0.13	0.09	-1.45	-0.3 - 0.04	$1.46x10^{-1}$	1.947X10-1	40.53	1344	412
R_lateralorbitofrontal	-0.12	0.10	-1.17	-0.31 - 0.08	$2.40x10^{-1}$	3.055X10-1	54.02	1347	412

**R_bankssts**	-0.11	0.10	-1.15	-0.31 - 0.08	2.51x10^{-1}	3.138X10-1	52.54	1315	406
**R_frontalpole**	-0.10	0.06	-1.67	-0.22 - 0.02	9.53x10^{-2}	1.430X10-1	0.00	1348	411
**L_bankssts**	-0.10	0.11	-0.89	-0.32 - 0.12	3.72x10^{-1}	4.401X10-1	62.31	1279	383
**R_entorhinal**	-0.09	0.09	-1.02	-0.28 - 0.09	3.10x10^{-1}	3.720X10-1	31.74	1069	296
**R_medialorbitofrontal**	-0.07	0.06	-1.22	-0.19 - 0.04	2.23x10^{-1}	2.882X10-1	0.00	1348	410
**R_middletemporal**	-0.07	0.08	-0.82	-0.23 - 0.09	4.13x10^{-1}	4.688X10-1	32.68	1336	410
**L_frontalpole**	-0.06	0.06	-1.05	-0.18 - 0.05	2.96x10^{-1}	3.603X10-1	0.00	1343	410
**L_lateralorbitofrontal**	-0.06	0.07	-0.82	-0.2 - 0.08	4.11x10^{-1}	4.688X10-1	19.52	1343	412
**R_caudalanteriorcingulate**	-0.06	0.11	-0.56	-0.27 - 0.15	5.76x10^{-1}	6.164X10-1	60.23	1346	411
**R_inferiortemporal**	-0.05	0.07	-0.64	-0.19 - 0.1	5.22x10^{-1}	5.695X10-1	21.85	1346	412
**R_temporalpole**	-0.03	0.06	-0.55	-0.15 - 0.08	5.86x10^{-1}	6.164X10-1	0.00	1344	410
**L_isthmuscingulate**	-0.02	0.08	-0.26	-0.18 - 0.13	7.91x10^{-1}	8.103X10-1	29.21	1343	411
**L_posteriorcingulate**	-0.01	0.06	-0.20	-0.13 - 0.11	8.45x10^{-1}	8.552X10-1	0.00	1344	411
**R_isthmuscingulate**	-0.01	0.08	-0.09	-0.17 - 0.15	9.31x10^{-1}	9.310X10-1	33.62	1345	411
**L_medialorbitofrontal**	0.04	0.08	0.51	-0.12 - 0.2	6.13x10^{-1}	6.357X10-1	33.86	1344	412
**L_rostralanteriorcingulate**	0.06	0.07	0.86	-0.08 - 0.2	3.88x10^{-1}	4.527X10-1	15.87	1342	411
**R_insula**	0.07	0.06	1.14	-0.05 - 0.19	2.54x10^{-1}	3.138X10-1	0.00	1323	405
**L_insula**	0.15	0.08	1.93	0 - 0.31	5.31x10^{-2}	8.578X10-2	29.34	1320	402
**L_caudalanteriorcingulate**	0.18	0.06	3.02	0.06 - 0.3	2.55x10^{-3}	**7.140X10-3**	0.00	1343	411
**R_rostralanteriorcingulate**	0.20	0.06	3.33	0.08 - 0.32	8.80x10^{-4}	**2.957X10-3**	0.00	1348	411

Abbreviations: SE, standard error; CI, confidence interval; I^2, heterogeneity index. Abbreviations for all cortical brain regions are provided in Supplementary Table 4. Uncorrected *p*-values are reported. Brain regions are ranked according to effect size (negative to positive). *P*-values were compared to an adjusted alpha level of $p<1.49 \times 10^{-4}$. Significant brain regions are **highlighted in red with an asterisk (*)**.

Supplementary Table 33. Full list of case-versus-control effect sizes for cortical thickness differences, including non-significant findings | 'MTLE-R' phenotype

Structure	Cohen's *d*	SE	Z score	95% CI	*P* value	FDR-adjusted *P* value	I^2	Number of controls	Number of cases
**R_precentral***	-0.52	0.09	-6.07	-0.69 - -0.35	**$1.25x10^{-9}$**	**2.10X10-8**	33.29	1293	337
**L_paracentral***	-0.50	0.10	-4.94	-0.7 - -0.3	**$7.67x10^{-7}$**	**9.20X10-6**	52.28	1292	338
**R_superiorparietal**	-0.49	0.17	-2.80	-0.83 - -0.15	$5.03x10^{-3}$	**1.62X10-2**	83.96	1295	338
**R_precuneus**	-0.46	0.12	-3.74	-0.7 - -0.22	$1.87x10^{-4}$	**1.09X10-3**	67.50	1296	338
**R_paracentral***	-0.42	0.06	-6.54	-0.55 - -0.3	**$6.24x10^{-11}$**	**1.31X10-9**	0.41	1296	338
**L_precentral***	-0.42	0.09	-4.60	-0.59 - -0.24	**$4.31x10^{-6}$**	**4.02X10-5**	40.04	1287	338
**L_superiorparietal**	-0.40	0.12	-3.22	-0.65 - -0.16	$1.27x10^{-3}$	**5.08X10-3**	68.40	1289	338
**L_lingual**	-0.38	0.14	-2.79	-0.65 - -0.11	$5.22x10^{-3}$	**1.62X10-2**	74.27	1291	338
**L_precuneus**	-0.38	0.11	-3.41	-0.6 - -0.16	$6.47x10^{-4}$	**2.72X10-3**	61.08	1291	338
**R_lateraloccipital**	-0.37	0.10	-3.75	-0.56 - -0.17	**$1.79x10^{-4}$**	**1.09X10-3**	48.50	1295	338
**L_supramarginal**	-0.36	0.10	-3.69	-0.55 - -0.17	$2.25x10^{-4}$	**1.16X10-3**	46.34	1248	321
**R_postcentral**	-0.36	0.15	-2.35	-0.66 - -0.06	$1.87x10^{-2}$	**4.36X10-2**	79.09	1292	338
**L_superiorfrontal**	**-0.35**	**0.09**	**-3.78**	**-0.54 - -0.17**	**$1.59x10^{-4}$**	**1.09X10-3**	**43.90**	**1291**	**337**
**L_postcentral**	-0.33	0.15	-2.25	-0.62 - -0.04	$2.43x10^{-2}$	5.23X10-2	77.84	1289	338
**L_transversetemporal***	-0.31	0.07	-4.25	-0.46 - -0.17	**$2.15x10^{-5}$**	**1.81X10-4**	15.61	1289	338
**R_caudalmiddlefrontal**	-0.31	0.08	-3.61	-0.47 - -0.14	$3.03x10^{-4}$	**1.41X10-3**	32.66	1296	338
**L_caudalmiddlefrontal**	-0.30	0.10	-3.07	-0.49 - -0.11	$2.15x10^{-3}$	**8.21X10-3**	48.96	1292	338
**R_superiorfrontal**	-0.30	0.12	-2.53	-0.53 - -0.07	$1.14x10^{-2}$	**3.09X10-2**	64.86	1293	338
**L_cuneus**	-0.29	0.14	-2.10	-0.55 - -0.02	$3.58x10^{-2}$	**7.16X10-2**	74.04	1290	338
**R_temporalpole**	-0.28	0.10	-2.73	-0.48 - -0.08	$6.41x10^{-3}$	**1.79X10-2**	53.29	1291	333
**R_lingual**	-0.28	0.08	-3.68	-0.43 - -0.13	$2.35x10^{-4}$	**1.16X10-3**	19.15	1295	338

**R_supramarginal**	-0.27	0.10	-2.73	-0.47 - -0.08	6.33x10-3	**1.79X10-2**	50.10	1240	326
**R_parsopercularis***	**-0.27**	**0.07**	**-3.80**	**-0.41 - -0.13**	**1.45x10-4**	**1.09X10-3**	**12.11**	**1295**	**338**
**L_pericalcarine**	-0.26	0.16	-1.58	-0.58 - 0.06	1.14x10-1	1.77X10-1	81.95	1292	338
**L_lateraloccipital**	-0.25	0.12	-2.03	-0.49 - -0.01	4.25x10-2	8.11X10-2	68.39	1292	338
**R_cuneus**	-0.25	0.08	-2.94	-0.41 - -0.08	3.31x10-3	**1.21X10-2**	32.45	1293	338
**R_inferiorparietal**	-0.24	0.14	-1.73	-0.52 - 0.03	8.41x10-2	1.36X10-1	75.92	1285	338
**R_transversetemporal**	-0.24	0.06	-3.73	-0.36 - -0.11	1.94x10-4	**1.09X10-3**	0.00	1296	338
**L_inferiorparietal**	-0.24	0.13	-1.88	-0.48 - 0.01	6.04x10-2	1.01X10-1	69.21	1281	336
**R_pericalcarine**	-0.23	0.08	-2.85	-0.39 - -0.07	4.39x10-3	**1.48X10-2**	26.42	1296	338
**R_superiortemporal**	-0.23	0.10	-2.29	-0.42 - -0.03	2.21x10-2	5.02X10-2	48.26	1197	322
**L_bankssts**	-0.22	0.15	-1.46	-0.51 - 0.08	1.44x10-1	2.03X10-1	77.76	1228	322
**L_parsorbitalis**	-0.19	0.10	-1.94	-0.38 - 0	5.27x10-2	9.75X10-2	48.79	1292	338
**R_entorhinal**	-0.18	0.10	-1.80	-0.38 - 0.02	7.23x10-2	1.19X10-1	35.11	1017	240
**R_bankssts**	-0.18	0.09	-1.92	-0.36 - 0	5.48x10-2	9.75X10-2	43.51	1263	333
**R_parahippocampal**	-0.18	0.06	-2.75	-0.3 - -0.05	6.05x10-3	**1.79X10-2**	0.00	1292	338
**R_fusiform**	-0.17	0.08	-2.24	-0.32 - -0.02	2.51x10-2	5.27X10-2	18.62	1292	338
**R_parstriangularis**	-0.17	0.09	-1.93	-0.33 - 0	5.41x10-2	9.75X10-2	34.31	1294	337
**L_fusiform**	-0.16	0.06	-2.52	-0.29 - -0.04	1.18x10-2	**3.10X10-2**	0.00	1287	337
**R_middletemporal**	-0.16	0.11	-1.47	-0.38 - 0.05	1.41x10-1	2.03X10-1	59.15	1284	337
**L_rostralmiddlefrontal**	-0.16	0.12	-1.27	-0.4 - 0.08	2.04x10-1	2.76X10-1	67.51	1292	337
**R_parsorbitalis**	-0.15	0.16	-0.99	-0.46 - 0.15	3.20x10-1	3.95X10-1	80.30	1296	338
**L_parsopercularis**	-0.15	0.10	-1.47	-0.36 - 0.05	1.43x10-1	2.03X10-1	55.57	1290	338
**L_parstriangularis**	-0.15	0.10	-1.46	-0.35 - 0.05	1.43x10-1	2.03X10-1	54.17	1292	338
**L_temporalpole**	-0.15	0.07	-2.04	-0.29 - -0.01	4.16x10-2	8.11X10-2	13.08	1287	337
**L_superiortemporal**	-0.14	0.12	-1.11	-0.38 - 0.11	2.67x10-1	3.56X10-1	66.85	1177	315
**L_entorhinal**	-0.11	0.08	-1.51	-0.26 - 0.03	1.31x10-1	2.00X10-1	0.00	1050	252
**L_middletemporal**	-0.11	0.11	-1.01	-0.33 - 0.11	3.13x10-1	3.92X10-1	59.15	1190	320

**R_inferiortemporal**	-0.11	0.10	-1.09	-0.31 - 0.09	2.77x10^{-1}	3.64X10-1	53.35	1294	338
**R_insula**	-0.11	0.10	-1.06	-0.31 - 0.09	2.89x10^{-1}	3.73X10-1	53.43	1257	333
**R_posteriorcingulate**	-0.09	0.10	-0.83	-0.29 - 0.12	4.07x10^{-1}	4.75X10-1	55.26	1296	337
**L_frontalpole**	-0.08	0.09	-0.89	-0.25 - 0.09	3.71x10^{-1}	4.39X10-1	36.44	1290	338
**L_inferiortemporal**	-0.06	0.06	-0.92	-0.18 - 0.07	3.59x10^{-1}	4.37X10-1	0.01	1290	338
**L_posteriorcingulate**	-0.06	0.06	-0.90	-0.18 - 0.07	3.70x10^{-1}	4.39X10-1	0.00	1292	338
**R_frontalpole**	-0.03	0.10	-0.29	-0.23 - 0.17	7.76x10^{-1}	8.37X10-1	51.78	1296	338
**R_rostralmiddlefrontal**	-0.03	0.10	-0.25	-0.23 - 0.18	8.04x10^{-1}	8.55X10-1	55.61	1296	338
**L_insula**	-0.02	0.06	-0.36	-0.15 - 0.1	7.17x10^{-1}	8.03X10-1	0.00	1254	332
**L_caudalanteriorcingulate**	-0.02	0.08	-0.28	-0.17 - 0.13	7.77x10^{-1}	8.37X10-1	23.29	1287	337
**L_lateralorbitofrontal**	-0.02	0.08	-0.23	-0.17 - 0.13	8.20x10^{-1}	8.56X10-1	21.73	1288	338
**L_parahippocampal**	-0.02	0.08	-0.19	-0.18 - 0.15	8.52x10^{-1}	8.62X10-1	29.23	1283	337
**L_rostralanteriorcingulate**	0.00	0.09	0.03	-0.18 - 0.18	9.73x10^{-1}	9.73X10-1	41.58	1287	335
**R_lateralorbitofrontal**	0.03	0.08	0.34	-0.13 - 0.18	7.36x10^{-1}	8.13X10-1	22.24	1293	338
**R_caudalanteriorcingulate**	0.06	0.12	0.50	-0.18 - 0.3	6.17x10^{-1}	7.00X10-1	66.90	1294	337
**L_isthmuscingulate**	0.08	0.08	1.03	-0.07 - 0.24	3.05x10^{-1}	3.88X10-1	25.30	1291	338
**L_medialorbitofrontal**	0.08	0.06	1.29	-0.04 - 0.21	1.98x10^{-1}	2.73X10-1	0.00	1274	336
**R_isthmuscingulate**	0.14	0.10	1.46	-0.05 - 0.34	1.45x10^{-1}	2.03X10-1	50.11	1293	338
**R_medialorbitofrontal**	0.15	0.09	1.63	-0.03 - 0.32	1.02x10^{-1}	1.62X10-1	40.09	1278	336
**R_rostralanteriorcingulate**	0.25	0.11	2.40	0.05 - 0.46	1.63x10^{-2}	**4.03X10-2**	56.47	1296	337

Abbreviations: SE, standard error; CI, confidence interval; I^2, heterogeneity index. Abbreviations for all cortical brain regions are provided in Supplementary Table 4. Uncorrected *p*-values are reported. Brain regions are ranked according to effect size (negative to positive). *P*-values were compared to an adjusted alpha level of $p<1.49x10^{-4}$. Significant brain regions are **highlighted in red with an asterisk (*)**.

Supplementary Table 34. Full list of case-versus-control effect sizes for cortical thickness differences, including non-significant findings | 'IGE' phenotype

Structure	Cohen's *d*	SE	Z score	95% CI	*P* value	FDR-adjusted *P* value	I^2	Number of controls	Number of cases
**R_precentral***	**-0.39**	**0.07**	**-5.44**	**-0.53 - -0.25**	**5.27x10-8**	**4.4X10-6**	**0.01**	**1044**	**295**
**L_precentral***	**-0.34**	**0.07**	**-4.78**	**-0.48 - -0.2**	**1.75x10-6**	**7.4X10-5**	**0.00**	**1043**	**297**
**R_temporalpole**	-0.28	0.09	-2.99	-0.46 - -0.09	2.82x10-3	**2.8X10-2**	33.03	1045	296
**L_caudalmiddlefrontal**	-0.20	0.07	-2.80	-0.34 - -0.06	5.08x10-3	**4.0X10-2**	1.52	1047	297
**L_entorhinal**	-0.19	0.08	-2.30	-0.35 - -0.03	2.15x10-2	1.2X10-1	0.00	820	242
**L_paracentral**	-0.18	0.07	-2.50	-0.32 - -0.04	1.25x10-2	8.8X10-2	0.00	1047	297
**R_caudalmiddlefrontal**	-0.17	0.09	-1.83	-0.36 - 0.01	6.66x10-2	2.7X10-1	36.20	1048	297
**L_temporalpole**	-0.17	0.08	-2.00	-0.33 - 0	4.53x10-2	2.2X10-1	20.13	1041	297
**R_entorhinal**	-0.15	0.13	-1.14	-0.41 - 0.11	2.54x10-1	5.5X10-1	55.22	810	240
**L_supramarginal**	-0.14	0.08	-1.84	-0.3 - 0.01	6.57x10-2	2.7X10-1	6.04	1003	274
**R_superiorparietal**	-0.13	0.07	-1.89	-0.27 - 0	5.81x10-2	2.7X10-1	0.00	1048	297
**L_superiorparietal**	-0.13	0.09	-1.56	-0.3 - 0.03	1.18x10-1	4.1X10-1	24.25	1045	297
**R_paracentral**	-0.13	0.10	-1.32	-0.33 - 0.06	1.86x10-1	4.7X10-1	43.15	1049	297
**L_inferiorparietal**	-0.13	0.09	-1.43	-0.3 - 0.05	1.52x10-1	4.5X10-1	28.42	1036	297
**L_lateraloccipital**	-0.13	0.15	-0.83	-0.42 - 0.17	4.05x10-1	7.2X10-1	74.54	1047	296
**R_parsorbitalis**	-0.12	0.07	-1.71	-0.26 - 0.02	8.70x10-2	3.2X10-1	0.00	1049	297
**L_middletemporal**	-0.11	0.07	-1.52	-0.26 - 0.03	1.28x10-1	4.3X10-1	0.00	963	279
**L_fusiform**	-0.11	0.09	-1.15	-0.29 - 0.08	2.50x10-1	5.5X10-1	34.99	1044	296
**R_lateralorbitofrontal**	-0.11	0.07	-1.51	-0.25 - 0.03	1.32x10-1	4.3X10-1	0.00	1046	297
**R_medialorbitofrontal**	-0.11	0.07	-1.48	-0.25 - 0.03	1.39x10-1	4.3X10-1	0.00	1031	292
**L_transversetemporal**	-0.10	0.07	-1.42	-0.24 - 0.04	1.56x10-1	4.5X10-1	0.00	1044	297
**R_superiorfrontal**	-0.10	0.08	-1.21	-0.26 - 0.06	2.27x10-1	5.4X10-1	19.57	1045	297

**R_inferiortemporal**	-0.09	0.07	-1.33	-0.23 - 0.04	1.84x10^{-1}	4.7X10-1	0.00	1047	297
**R_precuneus**	-0.09	0.08	-1.18	-0.24 - 0.06	2.39x10^{-1}	5.4X10-1	12.37	1049	297
**L_cuneus**	-0.09	0.10	-0.96	-0.28 - 0.1	3.38x10^{-1}	6.8X10-1	37.23	1045	297
**R_parsopercularis**	-0.08	0.07	-1.18	-0.22 - 0.06	2.37x10^{-1}	5.4X10-1	0.00	1047	297
**R_cuneus**	-0.08	0.08	-0.99	-0.24 - 0.08	3.25x10^{-1}	6.7X10-1	16.96	1047	297
**L_bankssts**	-0.08	0.11	-0.67	-0.3 - 0.15	5.03x10^{-1}	8.0X10-1	52.46	1014	272
**R_pericalcarine**	-0.07	0.12	-0.63	-0.3 - 0.15	5.26x10^{-1}	8.2X10-1	57.30	1049	296
**R_transversetemporal**	-0.07	0.10	-0.71	-0.27 - 0.13	4.76x10^{-1}	7.8X10-1	43.52	1049	297
**R_supramarginal**	-0.07	0.08	-0.93	-0.22 - 0.08	3.51x10^{-1}	6.8X10-1	0.00	993	270
**R_middletemporal**	-0.07	0.07	-0.92	-0.2 - 0.07	3.58x10^{-1}	6.8X10-1	0.00	1042	296
**L_insula**	-0.06	0.07	-0.88	-0.2 - 0.08	3.78x10^{-1}	7.0X10-1	0.01	1008	293
**R_lingual**	-0.06	0.10	-0.59	-0.26 - 0.14	5.54x10^{-1}	8.2X10-1	44.32	1048	296
**R_lateraloccipital**	-0.05	0.13	-0.35	-0.31 - 0.21	7.23x10^{-1}	8.9X10-1	67.05	1048	297
**R_rostralmiddlefrontal**	-0.05	0.07	-0.62	-0.19 - 0.1	5.35x10^{-1}	8.2X10-1	6.69	1049	297
**L_pericalcarine**	-0.04	0.10	-0.43	-0.25 - 0.16	6.67x10^{-1}	8.8X10-1	46.90	1047	297
**L_inferiortemporal**	-0.04	0.08	-0.46	-0.2 - 0.12	6.45x10^{-1}	8.8X10-1	20.41	1046	296
**L_rostralanteriorcingulate**	-0.04	0.11	-0.35	-0.25 - 0.17	7.24x10^{-1}	8.9X10-1	49.26	1042	294
**L_precuneus**	-0.04	0.08	-0.43	-0.2 - 0.13	6.66x10^{-1}	8.8X10-1	19.43	1046	297
**R_insula**	-0.03	0.07	-0.42	-0.17 - 0.11	6.74x10^{-1}	8.8X10-1	0.00	1010	290
**R_inferiorparietal**	-0.03	0.09	-0.30	-0.2 - 0.14	7.61x10^{-1}	9.0X10-1	26.09	1038	293
**R_rostralanteriorcingulate**	-0.03	0.08	-0.32	-0.18 - 0.13	7.47x10^{-1}	9.0X10-1	16.78	1047	297
**L_superiortemporal**	-0.03	0.10	-0.25	-0.22 - 0.17	7.99x10^{-1}	9.1X10-1	36.07	944	266
**L_parsopercularis**	-0.02	0.11	-0.22	-0.24 - 0.19	8.23x10^{-1}	9.1X10-1	50.93	1046	297
**R_parstriangularis**	-0.02	0.07	-0.30	-0.16 - 0.12	7.63x10^{-1}	9.0X10-1	0.00	1047	297
**L_frontalpole**	-0.02	0.09	-0.20	-0.19 - 0.15	8.40x10^{-1}	9.1X10-1	25.35	1045	297
**L_parstriangularis**	-0.01	0.08	-0.19	-0.17 - 0.14	8.51x10^{-1}	9.1X10-1	10.44	1047	297
**L_superiorfrontal**	-0.01	0.08	-0.19	-0.16 - 0.13	8.53x10^{-1}	9.1X10-1	7.27	1046	297

**L_lingual**	0.00	0.10	-0.03	-0.19 - 0.19	9.72x10^{-1}	9.9X10-1	39.38	1046	297
**L_medialorbitofrontal**	0.00	0.07	-0.02	-0.14 - 0.14	9.84x10^{-1}	9.9X10-1	0.01	1029	293
**R_parahippocampal**	0.00	0.07	0.01	-0.14 - 0.14	9.89x10^{-1}	9.9X10-1	0.00	1045	293
**L_parsorbitalis**	0.00	0.07	0.03	-0.14 - 0.14	9.77x10^{-1}	9.9X10-1	0.00	1047	296
**L_isthmuscingulate**	0.00	0.11	0.04	-0.21 - 0.22	9.68x10^{-1}	9.9X10-1	49.72	1046	296
**L_rostralmiddlefrontal**	0.02	0.09	0.22	-0.15 - 0.19	8.30x10^{-1}	9.1X10-1	24.37	1046	295
**R_isthmuscingulate**	0.02	0.10	0.21	-0.18 - 0.22	8.37x10^{-1}	9.1X10-1	44.09	1046	297
**R_fusiform**	0.03	0.10	0.26	-0.17 - 0.22	7.96x10^{-1}	9.1X10-1	39.10	1049	296
**L_posteriorcingulate**	0.03	0.09	0.36	-0.14 - 0.2	7.23x10^{-1}	8.9X10-1	28.25	1047	297
**R_frontalpole**	0.04	0.07	0.55	-0.1 - 0.18	5.82x10^{-1}	8.4X10-1	0.00	1049	297
**L_parahippocampal**	0.04	0.09	0.44	-0.14 - 0.22	6.60x10^{-1}	8.8X10-1	30.33	1038	294
**R_superiortemporal**	0.04	0.10	0.43	-0.15 - 0.24	6.71x10^{-1}	8.8X10-1	34.28	955	265
**R_caudalanteriorcingulate**	0.05	0.09	0.61	-0.12 - 0.22	5.44x10^{-1}	8.2X10-1	25.74	1047	297
**L_postcentral**	0.05	0.07	0.77	-0.08 - 0.19	4.43x10^{-1}	7.6X10-1	0.00	1044	296
**R_postcentral**	0.06	0.09	0.66	-0.11 - 0.23	5.07x10^{-1}	8.0X10-1	25.80	1043	297
**R_posteriorcingulate**	0.08	0.11	0.71	-0.14 - 0.31	4.75x10^{-1}	7.8X10-1	55.40	1049	296
**L_caudalanteriorcingulate**	0.09	0.08	1.03	-0.08 - 0.25	3.02x10^{-1}	6.3X10-1	21.80	1042	296
**L_lateralorbitofrontal**	0.09	0.07	1.30	-0.05 - 0.23	1.94x10^{-1}	4.8X10-1	0.00	1042	297
**R_bankssts**	0.10	0.12	0.82	-0.13 - 0.33	4.10x10^{-1}	7.2X10-1	56.54	1029	287

Abbreviations: SE, standard error; CI, confidence interval; I^2, heterogeneity index. Abbreviations for all cortical brain regions are provided in Supplementary Table 4. All uncorrected *p*-values are reported. Brain regions are ranked according to effect size (negative to positive). *P*-values were compared to an adjusted alpha level of $p<1.49 \times 10^{-4}$. Significant brain regions are **highlighted in red with an asterisk (*)**.

Supplementary Table 35. Full list of case-versus-control effect sizes for cortical thickness differences, including non-significant findings | 'All other epilepsies' phenotype

Structure	Cohen's *d*	SE	Z score	95% CI	*P* value	FDR-adjusted *P* value	I^2	Number of controls	Number of cases
**L_precentral***	-0.375	0.046	-8.237	-0.464 - -0.286	**1.76x10-16**	**1.48X10-14**	0.006	1442	997
**R_paracentral***	-0.351	0.045	-7.733	-0.44 - -0.262	**1.05x10-14**	**4.41X10-13**	0.003	1451	998
**R_precentral***	-0.348	0.045	-7.672	-0.437 - -0.259	**1.70x10-14**	**4.76X10-13**	0.000	1448	996
**L_caudalmiddlefrontal***	-0.291	0.045	-6.425	-0.38 - -0.202	**1.32x10-10**	**2.22X10-9**	0.000	1447	1000
**L_paracentral***	-0.257	0.045	-5.680	-0.346 - -0.168	**1.34x10-08**	**1.88X10-7**	0.000	1447	1000
**L_superiorfrontal***	-0.243	0.059	-4.138	-0.358 - -0.128	**3.51x10-05**	**1.55X10-4**	34.545	1446	999
**R_precuneus***	-0.238	0.053	-4.471	-0.343 - -0.134	**7.78x10-06**	**4.08X10-5**	22.378	1451	994
**R_superiorfrontal***	-0.235	0.052	-4.489	-0.337 - -0.132	**7.15x10-06**	**4.00X10-5**	20.049	1448	997
**R_cuneus***	-0.234	0.045	-5.186	-0.323 - -0.146	**2.15x10-07**	**2.58X10-6**	0.000	1449	996
**L_superiorparietal***	-0.224	0.045	-4.954	-0.313 - -0.136	**7.27x10-07**	**6.79X10-6**	0.001	1444	996
**R_superiorparietal***	-0.220	0.045	-4.864	-0.309 - -0.131	**1.15x10-06**	**9.66X10-6**	0.002	1450	997
**R_caudalmiddlefrontal***	-0.212	0.045	-4.699	-0.301 - -0.124	**2.62x10-06**	**2.00X10-5**	0.000	1451	998
**R_lateraloccipital***	-0.211	0.045	-4.659	-0.299 - -0.122	**3.18x10-06**	**2.15X10-5**	0.003	1450	997
**R_parstriangularis***	-0.210	0.045	-4.650	-0.299 - -0.122	**3.32x10-06**	**2.15X10-5**	0.003	1449	998
**R_supramarginal***	-0.206	0.047	-4.418	-0.297 - -0.115	**9.95x10-06**	**4.92X10-5**	0.000	1395	961
**L_transversetemporal**	-0.200	0.059	-3.405	-0.316 - -0.085	6.62x10-04	**2.14X10-3**	34.852	1444	1000
R_rostralmiddlefrontal*	-0.198	0.052	-3.778	-0.3 - -0.095	1.58x10-04	5.31X10-4	19.944	1451	997
**R_entorhinal***	-0.197	0.058	-3.378	-0.312 - -0.083	7.30x10-04	**2.27X10-3**	23.333	1183	901
**R_transversetemporal***	-0.180	0.045	-3.982	-0.269 - -0.091	**6.84x10-05**	**2.85X10-4**	0.012	1451	998

R_lingual*	-0.180	0.045	-3.972	-0.268 - -0.091	**7.12x10-05**	**2.85X10-4**	0.013	1450	996
L_precuneus*	-0.178	0.047	-3.819	-0.27 - -0.087	**1.34x10-04**	**4.89X10-4**	4.474	1446	998
R_lateralorbitofrontal	-0.167	0.055	-3.069	-0.274 - -0.06	2.15x10-03	**5.83X10-3**	25.473	1448	996
L_pericalcarine	-0.160	0.054	-2.957	-0.266 - -0.054	3.10x10-03	**7.89X10-3**	24.415	1447	999
R_pericalcarine	-0.158	0.048	-3.300	-0.252 - -0.064	9.68x10-04	**2.90X10-3**	8.516	1451	997
R_parsorbitalis	-0.156	0.052	-3.001	-0.258 - -0.054	2.69x10-03	**7.06X10-3**	19.313	1451	996
L_lateraloccipital	-0.153	0.048	-3.197	-0.247 - -0.059	1.39x10-03	**4.03X10-3**	8.160	1447	1000
L_entorhinal	-0.149	0.084	-1.770	-0.314 - 0.016	0.077	1.22X10-1	61.910	1210	913
L_cuneus	-0.142	0.049	-2.893	-0.239 - -0.046	0.004	**9.88X10-3**	11.914	1445	1000
L_parstriangularis	-0.141	0.045	-3.116	-0.229 - -0.052	0.002	**5.60X10-3**	0.002	1447	1000
L_supramarginal	-0.138	0.059	-2.346	-0.254 - -0.023	0.019	**3.80X10-2**	31.971	1403	961
L_parsorbitalis	-0.128	0.045	-2.840	-0.216 - -0.04	0.005	**1.20X10-2**	0.005	1447	999
R_temporalpole	-0.126	0.047	-2.680	-0.218 - -0.034	0.007	**1.63X10-2**	5.439	1448	995
R_parsopercularis	-0.125	0.047	-2.643	-0.217 - -0.032	0.008	**1.82X10-2**	6.373	1450	998
L_parsopercularis	-0.122	0.047	-2.578	-0.215 - -0.029	0.010	**2.21X10-2**	6.861	1445	999
R_inferiorparietal	-0.118	0.050	-2.372	-0.216 - -0.021	0.018	**3.73X10-2**	13.162	1440	989
R_superiortemporal	-0.110	0.059	-1.888	-0.225 - 0.004	0.059	9.72X10-2	30.871	1354	959
R_middletemporal	-0.102	0.045	-2.261	-0.191 - -0.014	0.024	**4.58X10-2**	0.000	1440	993
R_medialorbitofrontal	-0.101	0.054	-1.848	-0.207 - 0.006	0.065	1.05X10-1	24.276	1433	973
L_lingual	-0.099	0.045	-2.201	-0.188 - -0.011	0.028	5.23X10-2	0.001	1446	1000
R_frontalpole	-0.098	0.045	-2.170	-0.186 - -0.009	0.030	5.48X10-2	0.002	1451	997
L_rostralmiddlefrontal	-0.097	0.062	-1.570	-0.218 - 0.024	0.116	1.71X10-1	40.582	1447	999
L_inferiorparietal	-0.096	0.045	-2.122	-0.185 - -0.007	0.034	6.08X10-2	0.011	1436	995
L_fusiform	-0.095	0.045	-2.106	-0.184 - -0.007	0.035	6.13X10-2	0.002	1445	999
L_isthmuscingulate	-0.093	0.057	-1.629	-0.204 - 0.019	0.103	1.57X10-1	31.030	1446	998
L_superiortemporal	-0.092	0.059	-1.543	-0.208 - 0.025	0.123	1.78X10-1	32.164	1330	952
L_frontalpole	-0.089	0.046	-1.954	-0.179 - 0	0.051	8.57X10-2	2.096	1445	999

**L_posteriorcingulate**	-0.089	0.061	-1.456	-0.209 - 0.031	0.145	2.03X10-1	39.803	1447	999
**L_temporalpole**	-0.088	0.069	-1.282	-0.223 - 0.047	0.200	2.60X10-1	51.484	1442	997
**R_fusiform**	-0.086	0.062	-1.393	-0.207 - 0.035	0.164	2.22X10-1	40.231	1451	997
**R_bankssts**	-0.081	0.063	-1.279	-0.205 - 0.043	0.201	2.60X10-1	42.124	1421	983
**R_parahippocampal**	-0.076	0.045	-1.679	-0.164 - 0.013	0.093	1.45X10-1	0.000	1447	996
**L_middletemporal**	-0.074	0.046	-1.590	-0.165 - 0.017	0.112	1.68X10-1	0.014	1345	958
**R_insula**	-0.069	0.046	-1.518	-0.159 - 0.02	0.129	1.84X10-1	0.018	1412	990
**L_postcentral**	-0.065	0.045	-1.434	-0.153 - 0.024	0.152	2.09X10-1	0.005	1444	997
**L_lateralorbitofrontal**	-0.065	0.064	-1.005	-0.191 - 0.061	0.315	3.95X10-1	44.841	1443	999
**R_posteriorcingulate**	-0.061	0.062	-0.986	-0.184 - 0.061	0.324	4.00X10-1	41.687	1451	998
**L_parahippocampal**	-0.060	0.045	-1.328	-0.149 - 0.029	0.184	2.45X10-1	0.000	1438	995
**R_inferiortemporal**	-0.035	0.049	-0.701	-0.131 - 0.062	0.483	5.71X10-1	12.480	1449	996
**R_postcentral**	-0.024	0.045	-0.529	-0.112 - 0.065	0.597	6.97X10-1	0.000	1447	996
**L_rostralanteriorcingulate**	-0.023	0.068	-0.344	-0.156 - 0.109	0.731	8.22X10-1	49.941	1442	994
**R_isthmuscingulate**	-0.016	0.055	-0.287	-0.124 - 0.092	0.774	8.37X10-1	27.792	1448	997
**L_insula**	-0.015	0.053	-0.284	-0.118 - 0.088	0.777	8.37X10-1	20.025	1409	985
**L_medialorbitofrontal**	-0.014	0.046	-0.313	-0.104 - 0.075	0.754	8.33X10-1	0.000	1429	975
**L_bankssts**	-0.007	0.064	-0.103	-0.132 - 0.119	0.918	9.18X10-1	40.639	1381	937
**L_inferiortemporal**	-0.007	0.054	-0.121	-0.112 - 0.099	0.903	9.18X10-1	24.312	1446	996
**R_caudalanteriorcingulate**	0.007	0.048	0.140	-0.088 - 0.102	0.888	9.18X10-1	9.893	1449	997
**R_rostralanteriorcingulate**	0.023	0.067	0.340	-0.108 - 0.154	0.734	8.22X10-1	48.820	1450	996
**L_caudalanteriorcingulate**	0.050	0.052	0.962	-0.052 - 0.153	0.336	4.09X10-1	20.078	1442	987

Abbreviations: SE, standard error; CI, confidence interval; I^2, heterogeneity index. Abbreviations for all cortical brain regions are provided in Supplementary Table 4. All uncorrected *p*-values are reported. Brain regions are ranked according to effect size (negative to positive). *P*-values were compared to an adjusted alpha level of $p<1.49\times10^{-4}$. Significant brain regions are **highlighted in red with an asterisk (*)**.

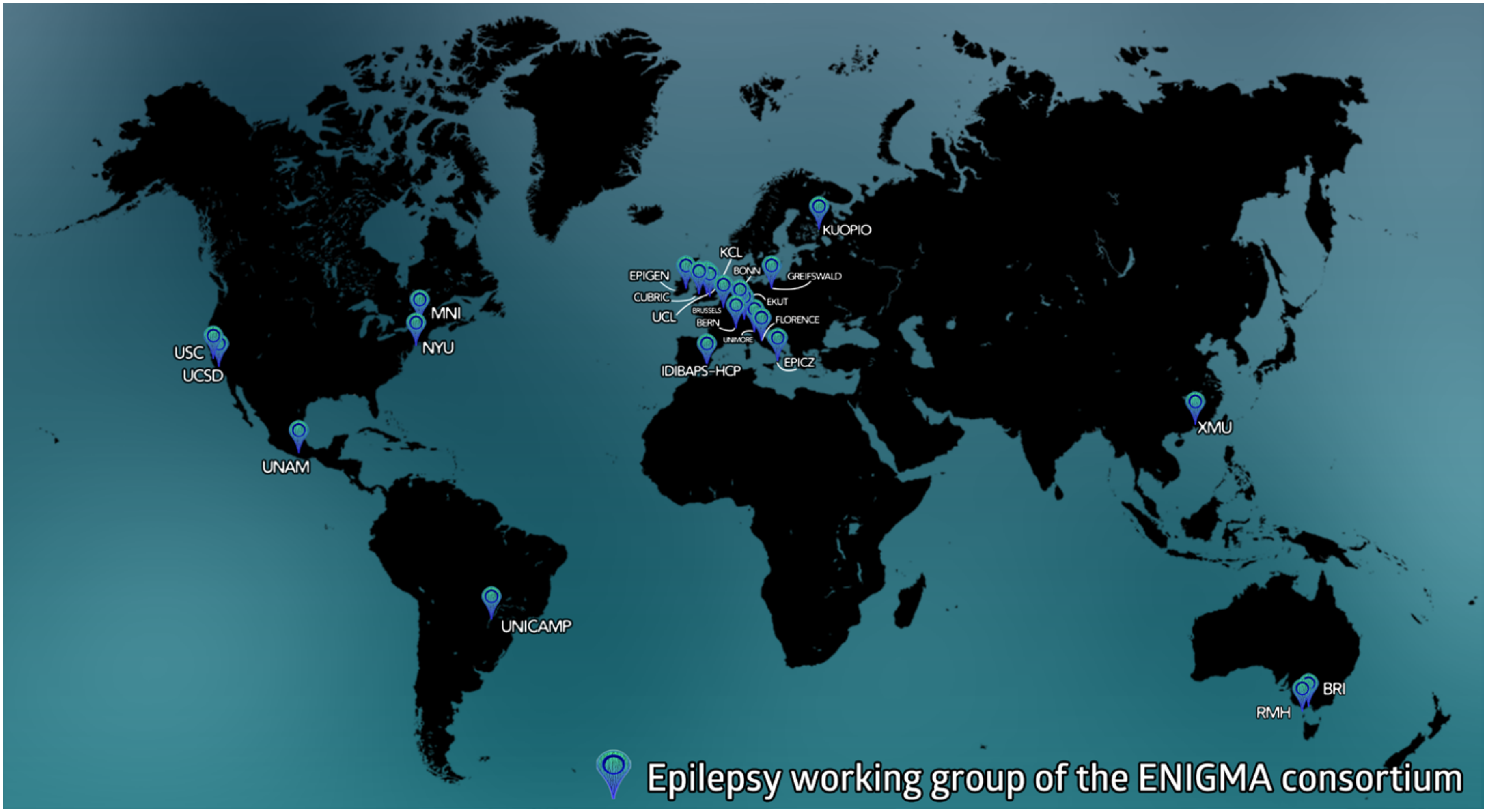

Supplementary Figure 1. ENIGMA-Epilepsy Worldwide Map. The initiative unites **24 research centres** from Europe (Barcelona, IDIBAPS; Bern; Bonn; Brussels; Cardiff, CUBRIC; Cantazaro, EPICZ; Dublin, EPIGEN; King's College London, KCL; Kuopio; Florence; Greifswald; Modena, UNIMORE; Tubingen), Australia (Royal Melbourne Hospital, RMH; Brain Research Institute, BRI), North America (University of California San Diego, UCSD; New York University, NYU), Central America (University Autonomous de Mexico, UNAM), South America (University of Campinas, UNICAMP) and South East Asia (Xiamen, XMU). The effort is coordinated by Christopher Whelan at the University of Southern California (USC) and Sanjay Sisodiya at University College London (UCL).

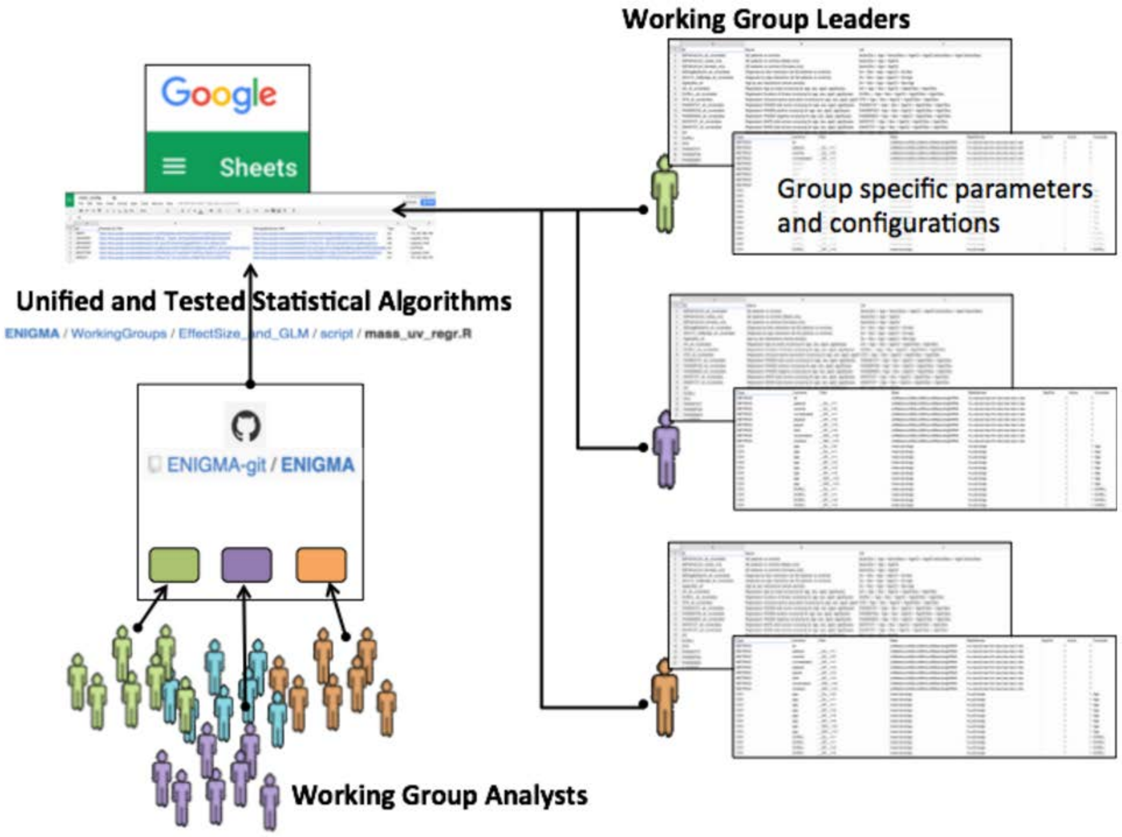

Supplementary Figure 2. Illustration and description of the coordinated ENIGMA meta-analysis framework. A standardized ENIGMA bash script is circulated to all ENIGMA-Epilepsy Working Group Analysts. The ENIGMA-Epilepsy Working Group leader configures any statistical tests or variables specific to their analysis using an online Google Sheet, and sets the tests they wish to run using a binary "ACTIVE" variable (0=do not run this test; 1=run this test). The local Working Group Analysts then execute their ENIGMA bash scripts, receiving statistical outputs for all 'ACTIVE' algorithms. The Google Sheet can be updated to include additional tests, without any requirement for re-circulation or re-configuration of the script by the local Working Group Analysts.

Supplementary Figures 3-12 are available via the ENIGMA-Epilepsy website, at: http://enigma.ini.usc.edu/ongoing/enigma-epilepsy/enigma-epilepsy-gm/
